# Four decades of intensifying Southern Ocean swells along the Pacific coast of the Americas

**DOI:** 10.1038/s41467-026-71813-1

**Published:** 2026-04-29

**Authors:** Hector Lobeto, Melisa Menendez, Alvaro Semedo, Iñigo J. Losada, Gil Lemos

**Affiliations:** 1 IHCantabria - Instituto de Hidráulica Ambiental de la Universidad de Cantabria, Santander, Spain; 2 Department of Coastal and Urban Risk and Resilience, IHE Delft Institute for Water Education, Delft, The Netherlands; 3 Instituto Dom Luiz (IDL), Faculdade de Ciências, Universidade de Lisboa, Lisbon, Portugal

**Keywords:** Natural hazards, Environmental impact

## Abstract

Swells generated in the Southern Ocean regularly impact the Pacific coasts of the Americas, with the most energetic events causing severe infrastructure damage, coastal flooding, and loss of life. This study presents a detailed, comprehensive analysis of these high-energy swells along this coastline, characterizing their primary characteristics and examining their variability across multiple timescales. These swells occur most frequently during austral winter, with a considerable proportion also occurring in austral spring and autumn. The Southern Annular Mode exerts a strong influence, with its positive phase (SAM + ) being associated with more energetic swells. Significant positive trends in wave height, power, and occurrence rate along much of the studied coastline in recent decades highlight the increasing risk posed by these swell events. Our findings offer valuable insights for enhancing coastal resilience and informing effective risk management strategies in response to this coastal hazard.

## Abstract (web summary)

Powerful swells generated in the Southern Ocean regularly reach the Pacific coasts of the Americas. This study shows that these events are becoming stronger and more frequent, increasing the risk of coastal impacts.

## Introduction

A remarkable ocean wave event in early May 2015 impacted a large portion of the Pacific coasts of South and Central America. Its consequences were devastating, not only because of its intrinsic energy content but also due to its coincidence with large spring tidal amplitudes, leading to severe flooding. This combination resulted in extensive erosion; damage to property, infrastructure, and ecosystems; and loss of life, with dozens of fatalities reported^1,2^. This event was not an isolated occurrence. Similarly, in May 2007, a highly energetic wave event generated south of South Africa propagated more than 5000 km across the Indian Ocean, flooding 88 islands across 18 atolls of the Maldives. The episode displaced more than 1600 people, damaged over 500 houses, and disrupted critical infrastructure^3^. Despite occurring in different ocean basins and affecting different coastal settings, these wave events exhibit common characteristics, pointing to a shared underlying nature. Understanding how such events originate, travel across ocean basins, and vary upon reaching the coast is essential to place their impacts in a broader and more systematic context.

At a fundamental level, ocean wind waves (hereinafter referred to as waves) are generated by energy transfer from the wind to the sea surface and can propagate over very long distances. Waves arriving at a coastal location may have been generated locally or remotely. For example, waves arriving at the coast of California can be generated by low-pressure systems in the middle of the North Pacific Ocean, with waves reaching the shore as well-organized waves, or by a land-falling tropical storm, with waves impacting the coast in a convoluted and chaotic manner^4^. Both the distance from the generation area and the duration of the overlying winds contribute to the degree of development of the waves reaching the coast^5^. Waves in a developing stage are known as wind seas, whereas developed waves outside the generation area are known as swells. Swells escape from the generation area, dispersing in frequency and direction, and radiating across all ocean basins as wave trains characterized by different heights, periods, and directions. Swells can travel thousands of kilometers across entire ocean basins, following great circle paths^6,7^, with deviations due to ocean currents and marginal energy dissipation^8–10^. As a result of the dispersion and dissipation of swells, those reaching very distant coasts are characterized by attenuated wave heights and very long periods. Note that the power (energy flux) of waves is a function of the wave height and the wave period to the first and second orders, respectively^5^. Hence, despite wave heights being moderated at long distances, the combination with large periods allows a significant amount of energy to reach the coastline. Additionally, waves with longer periods experience more shoaling than shorter waves transporting a similar amount of energy, resulting in a higher wave height at breaking and a larger release of energy upon dissipation. Moreover, distant long mature swells usually exhibit narrower spectra, which enhances the generation of infragravity waves. The height of these infragravity waves, released through the bound-wave mechanism, and for which a correlation with the product of the wave height and the squared period of the generating waves has been found^11,12^, may also be relevant and capable of damaging coastal zones^13^.

**Fig. 1:**
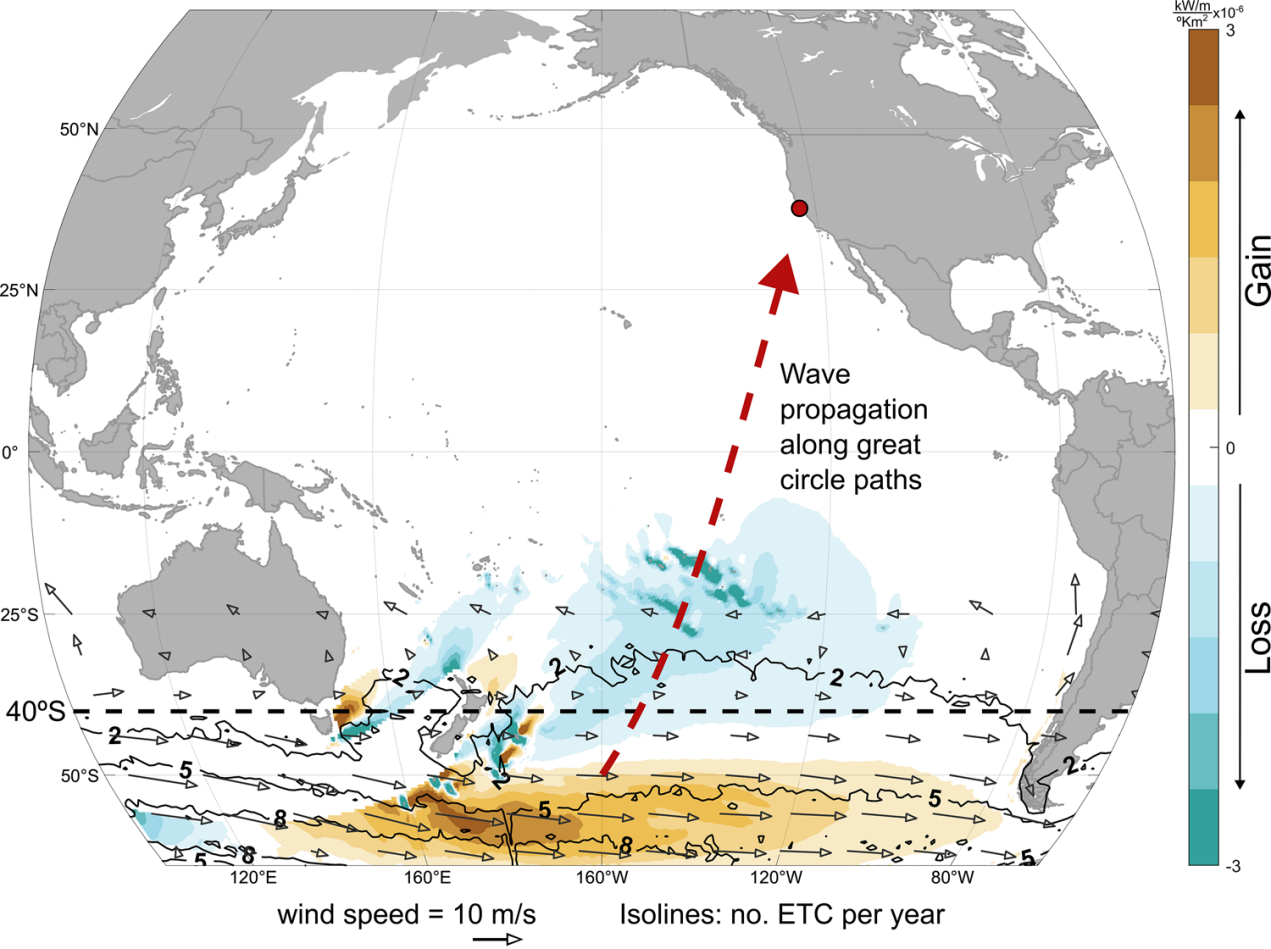
Generation and propagation of Southern Ocean swells. The isolines indicate the average number of extratropical cyclones (ETCs) per year. The arrows represent the average surface wind speed and direction. The colors depict the mean wave energy gains and losses. The red dot indicates the location toward which wave energy propagates. The red arrow illustrates the wave propagation directions from the Southern Ocean to the target point.

These damaging ocean wave events are generated thousands of kilometers away in the Southern Ocean (SO). The SO is the southernmost body of water; it surrounds Antarctica and connects the three main ocean basins. For practical reasons, in the present study, we consider the southernmost parts of the Atlantic, Pacific, and Indian Oceans as part of the SO, defining its limit at 40°S latitude^14^. This ocean region is, from a wave climate perspective, the most energetic region^14–16^. The strong westerly winds blowing all year round^16,17^, together with the intensity and number of storms moving eastward^18–20^, make it one of the main swell-generating regions^21^. Additionally, the fact that the SO is mostly uninterrupted around the globe favors the existence of vast swell generation areas with almost unlimited fetch. After generation, these swells radiate northward to all ocean basins, reaching coastal locations far north of the equator^22–26^. The fingerprints of these swells can be most clearly observed along the western boundaries of the continents because of their predominant eastward propagation direction. Figure 1 shows the generation area of the SO swells reaching a point located on the coast of California (USA). The colors in the figure represent the mean gain (brown) and loss (blue) of the wave energy propagating away from any point in the Southern Hemisphere (SH) to the target location from 1979 to 2023, calculated as the difference between the incoming and outgoing energy flux along the great circle^22^. The results show that SO swells are generated mainly south of 40°S, where the density of extratropical storms is the highest (isolines in Fig. 1). Additionally, this region is also affected by persistent winds with high wind speeds (arrows in Fig. 1), with annual mean values exceeding 10 m/s.

Southern Ocean swells can affect both insular and continental coastlines across all ocean basins. They propagate thousands of kilometers to reach distant shores in the Pacific, Indian, and Atlantic Oceans, with the capacity to generate impacts in locations as diverse as low-lying atolls or continental margins. Owing to the considerable amount of energy carried by these swell events, when they reach land, they can cause extensive damage to the coast (e.g., erosion^27–30^) or substantial water level elevations induced by wave setup^3,31,32^, which can lead to coastal flooding. Different total water level components, such as those induced by astronomical tides^33^, interannual sea-level oscillations (e.g., due to ENSO^34,35^), storm surges^36,37^, and projected mean sea-level rise^38,39^, can occur in combination with large wave setup^40–43^, thereby amplifying coastal water levels and the hazard potential. The impacts arising from SO swells differ markedly depending on the coastal setting. In island nations, a single event can produce widespread flooding^3,44^, lagoon submersions^45,46^, ecosystem damage^45,47^, and severe economic losses^3,48^, sometimes accompanied by community displacement and major disruptions to livelihoods^44,49^. The existential nature of these impacts is expected to worsen with rising sea levels, threatening the long-term habitability of atoll islands^48^. In contrast, in continental margins, impacts are generally not existential but translate into recurrent coastal erosion^2,29,30^, flooding of communities^31,32^, casualties, damage to infrastructure, and disruption of maritime activities^1^. In particular, the Pacific coast of the Americas is one of the continental coastal regions in the world most affected by these events^1,50^. The western coasts of the American continents are fully exposed to energetic swells from the SO^16,22^, which can be generated either in the Pacific or eastern Indian regions of the SO (Fig. 1). These coasts will be the study area for this research.

Over the years, numerous SO swell events have caused some form of damage upon reaching the aforementioned coasts. Table 1 lists numerous such events, along with a brief description of the damage caused. The events listed here were found in previous studies, reports, digital newspaper archives, or disaster databases, as referenced in the last column of Table 1. While the origin was explicitly specified in the reports for many of the listed events, for the remaining ones it was corroborated by analyzing the available wave data (buoys and hindcasts), particularly the direction and period of the sea state at the time of swell arrival. Although this information does not necessarily reflect all the countries affected and/or all the damage caused, it provides a clear indication of the severity and relevance of these events. Additionally, the recorded arrival date may vary slightly across different countries. In most cases, the energetic swells from the SO led to coastal flooding, resulting in damage to critical public infrastructure and private assets, as well as to the disruption or interruption of activities and consequent economic losses. Furthermore, these strong swells occasionally cause the capsizing of vessels and the drowning of individuals. Exceptionally energetic events can reach very high latitudes and still cause severe impacts, such as those in 2011 on the coasts of North America (see Event no. 8 in Table 1). However, beyond these impacts, on some coasts, these swells can also be appreciated for their recreational value, such as the optimal conditions they create for surfing^51^.

**Table 1:** Harmful events generated by swells from the Southern Ocean

Ev	Approx. date	Country	Damage	Point	Source
1	22/05/1981	Costa Rica	Infrastructure damage, Economic losses	P12	DesInventar Disaster Information Management System (https://www.desinventar.net/)
2	27/05/1985	Chile	Infrastructure damage, Property damage, Economic losses, Coastal erosion	P20	Atlas de oleaje de Chile^102^
3	21/06/1994	Chile	Economic losses, Coastal erosion	P20	Atlas de oleaje de Chile^102^
4	03/06/1999	Guatemala	Property damage, Human displacement	P11	DesInventar Disaster Information Management System (https://www.desinventar.net/)
5	19/06/2006	Peru, Guatemala, El Salvador, Nicaragua, Honduras	Infrastructure damage, Property damage, Economic losses, Human displacement	P11	Hispagua Sistema Español de Información sobre el Agua (https://hispagua.cedex.es/documentacion/noticia/45381), Sardon et al.^103^
6	08/04/2007	Peru	Property damage	P18	Sardon et al.^103^
7	20/05/2011	Mexico	Local business	P10	DesInventar Disaster Information Management System (https://www.desinventar.net/)
8	01/09/2011	California	Fatalities and Injuries	P5	Patch.com (link)
9	04/07/2013	Chile	Property damage	P20	Atlas de oleaje de Chile^102^
10	06/06/2013	Peru	Infrastructure damage, Property damage, Economic losses	P17	El Correo (link)
11	02/07/2014	Chile	Infrastructure damage, Economic losses	P20	Atlas de oleaje de Chile^102^
12	02/05/2015	Chile, Peru, Ecuador, Colombia, Panama, Costa Rica, El Salvador, Guatemala, Mexico	Infrastructure damage, Property damage, Economic losses, Human displacement, Fatalities, and Injuries	P11	Government of Mexico (link), DW (link), EFEVerde (link), Diairo Co Latino (link)
13	23/06/2016	Mexico, Nicaragua, El Salvador	Infrastructure damage, Economic losses	P11	Government of Mexico (link), El 19 Digital (link), La Prensa Gráfica (link)
14	16/08/2017	Mexico	Infrastructure damage, Economic losses	P9	El Sur Acapulco (link)
15	23/07/2018	Mexico, El Salvador	Infrastructure damage, Economic losses	P10	El Sol de Acapulco (link), La Prensa Gráfica (link)
16	16/08/2018	Mexico, El Salvador	Infrastructure damage, Economic losses, Fatalities, and Injuries	P10	El Sol de Mexico (link), La prensa Gráfica (link)
17	21/05/2019	Mexico	Infrastructure damage, Economic losses	P10	Infobae (link)
18	27/05/2023	Peru, Guatemala	Infrastructure damage, Economic losses	P11	Prensa Libre (link)
19	08/06/2023	Guatemala	Infrastructure damage, Property damage, Economic losses, Human displacement	P11	Emisoras Unidas (link)
20	02/09/2023	Panamá, El Salvador	Infrastructure damage, Property damage, Economic losses	P13	Televisora Nacional (link)

The table includes: approximate date, affected country/countries, damages caused, nearest analysis point, and source.

Despite the demonstrated relevance of these events to the Pacific coast of the Americas and being a well-known phenomenon, few studies, to our knowledge, have focused on these events. The primary reason is likely the lack of necessary data and analytical methods to conduct such a study with the required level of detail and complexity. Over the years, research on wave dynamics and propagation has advanced significantly, building upon the pioneering studies of Snodgrass et al.^7^ in the Pacific. However, traditional approaches relying on integrated data fail to properly identify these SO swell events, as they are often masked by other, more frequent waves of different origins along the Pacific American coastline, an effect that becomes increasingly evident farther north.

Here, we analyze these swell events in detail using a methodology based on spectral data that enables their individual identification and characterization along the entire coast of the Americas. We examine their main properties, including wave height, period, and direction, and provide a comprehensive assessment of their climate variability across different time scales, with particular emphasis on long-term trends.

## Results

### Swell event identification

Southern Ocean swells were analyzed using a 45-year (1979–2023) wave hindcast with an hourly temporal resolution. This product provides integrated wave parameters and wave spectral information approximately every 25 km along the study coast (more details are provided in the “Methods” section). In particular, this work used spectral partition data to conduct the analysis. A spectral partition provides information on each individual wave train at an ocean point at each instant, which is essential for isolating a specific type of event (i.e., a wave system^52^). For this analysis, partitions representing the wind-sea (if present) and up to five swells were considered. Each partition was characterized by its wave height, period, and direction, represented through the following parameters: significant wave height (${H}_{s}$), peak period (${T}_{p}$), and peak direction (${\theta }_{p}$), respectively (a complete definition of the spectral partition and integrated parameters is provided in the “Methods” section). The study was conducted at twenty-two coastal target points selected along the Pacific coast of the Americas (Supplementary Fig. 1, Supplementary Table 1). These points are representative of the entire coastline while ensuring sufficient distance from wave-breaking shallow-water zones. At the same time, the points are located at depths sufficient to minimize, even for long-period waves (${T}_{p}$ longer than 14 s), any pronounced shoaling processes.

The method for isolating SO swell events using spectral partitions relies on analyzing the time series of the ${T}_{p}$ parameter^53–55^, which represents the period of the most energetic waves. Swell events can be detected through a continuous decrease in this parameter (see Supplementary Fig. 2). On the basis of this principle, a search algorithm was developed to isolate SO swells at any ocean point. Specifically, using data from spectral partitions, the algorithm identifies events by detecting a smooth and continuous temporal decline in ${T}_{p}$. Then, it verifies the origin of the identified events using linear propagation theory. Only swells originating in the SO, below 40°S, were selected (see the “Methods” section for a detailed description of the algorithm).

Using this approach, the 20 most powerful events reaching each coastal target point annually were identified (see “Methods” section), resulting in data for a total of 20 × 45 = 900 swell events at each location. As illustrated by the example event outlined in Supplementary Fig. 2, SO swell events typically reach coastal target points with very long ${T}_{p}$ values, often exceeding 20 s, before continuously decreasing. This pattern arises from the wave dispersion characteristic, where longer-period waves travel faster than shorter-period waves do, with the former arriving at the target point earlier than the latter. Furthermore, the ${H}_{s}$ of the event increases with time, peaking sometime after the arrival of the swell and not coinciding with the maximum ${T}_{p}$ values. The wave direction remains stable during swell events, with any observed variations most likely reflecting the spatial evolution of the generating atmospheric systems in the SO^55^. Owing to predominantly eastward storm tracks, the swell direction at the coast often shifts counterclockwise upon arrival. Despite this temporal evolution, to assess the maximum impact potential of these events, each SO swell was characterized by the ${H}_{s}$, ${T}_{p}$, and ${\theta }_{p}$ values corresponding to the peak spectral energy density of the event, i.e., when ${H}_{s}$ is at its maximum.

Importantly, the selection of the most powerful swells was based on the premise that these events have the potential to inflict damage. To validate this assumption, all events listed in Table 1 were cross-referenced with those identified by the algorithm. Every listed event corresponded to a swell included in the generated dataset, reinforcing confidence that the selected events, which constitute the basis of the analysis, were indeed capable of causing damage to the study coast. Nevertheless, these events are referred to as potentially damaging events, as their occurrence alone does not necessarily result in damage. Additional factors, such as interactions with other dynamics (e.g., tides, large-scale climate variability modes such as ENSO) and existing local coastal exposure, also play crucial roles.

The ability of the model to capture the incoming wave energy from the SO was evaluated through a comparison with data recorded by nine wave buoys (see “Methods” section), which provided wave spectral information and had a sufficiently long recorded period. The results show strong correlations (mean value > 0.85), moderate root mean square errors (RMSEs; mean value < 0.13 m) and slight positive biases (mean value < 0.05 m) for all buoys when daily incoming wave energy throughout the study period is compared (gray dots in Supplementary Fig. 3). Model output performance is similar when focusing specifically on the daily energy recorded during the selected SO swell events at the buoy locations (red dots in Supplementary Fig. 3).

### Main swell characteristics

**Fig. 2:**
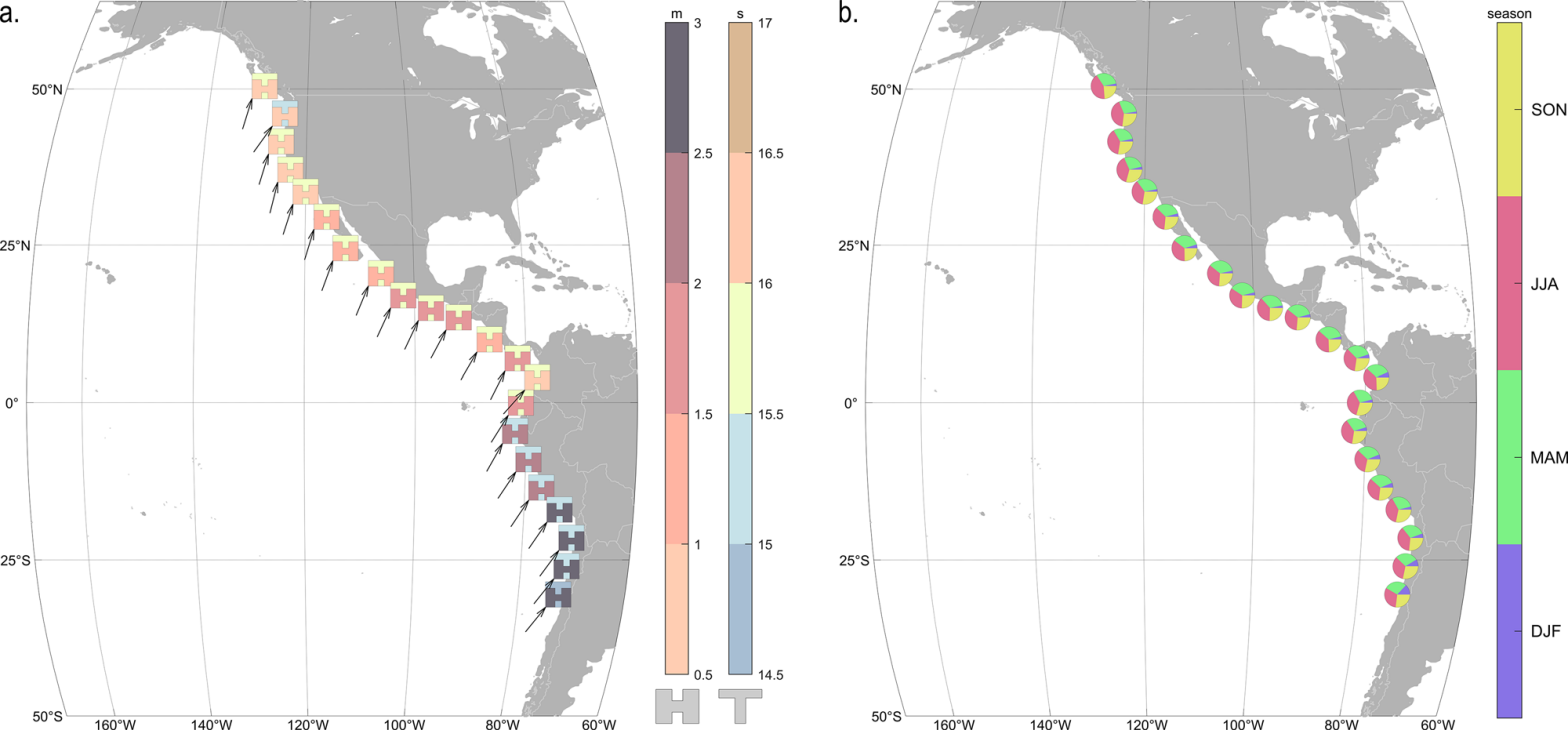
Mean characteristics and seasonal distribution of Southern Ocean swells. **a** Mean significant wave height (H within the square), mean peak period (T within the square), and mean peak direction (arrows) of the 900 events recorded at each target point. **b** Proportion of events recorded during March–April–May (MAM), June–July–August (JJA), September–October–November (SON), and December–January–February (DJF).

The average characteristics of the most powerful swell events reaching the coast of the study area change progressively as the distance from their generation area in the SO increases. Figure 2 displays the mean wave height, period, and direction of the 900 selected SO swell events at each coastal point. The wave height decreases northward because of the dispersion and dissipation experienced by swells during propagation. Despite traveling thousands of kilometers from their generation, the average ${H}_{s}$ of these events still exceeds 1.5 m along the coast of Central America and 0.5 m along the coast of North America. Importantly, Fig. 2 shows only the properties of swells originating from the SO. In most cases, these waves arrive at the target locations alongside waves generated in other regions, thereby increasing the total incident wave height. In fact, in the Northern Hemisphere (NH), SO swells are typically not the dominant wave system, and their characteristics are therefore often masked by the simultaneous presence of NH-generated waves^25,26,56,57^. The difference between the total wave height and the SO swell wave height at the moment these events reach the coast can be as high as 2 m at the northernmost locations (Supplementary Fig. 4). However, in areas closer to the SO, where these events often dominate the wave climate, this difference is considerably lower (<0.25 m). Note that the differences shown in Supplementary Fig. 4 are computed relative to the total wave conditions at the time of SO swell arrival and do not necessarily represent the contrast with local maximum wave conditions.

Unlike wave height, the period of these swells increases northward, as only the longest-period swells can travel the greatest distances. Along the Pacific coasts of the Americas, characteristic ${T}_{p}$ values generally exceed 15 s, with minor variations along the coastline, primarily following a south-to-north increasing gradient. Similar to wave height, the differences between the total wave periods and the SO swell periods at the moment of SO swell arrival are more pronounced along the coast of North America. In this region, the total ${T}_{p}$ tends to be significantly shorter (by more than 3 s) than that associated with SO swells.

Although the swell incoming direction is predominantly from the southwest at all locations, the southern component becomes increasingly prominent relative to the western component with distance northward. The directional offset of SO swells relative to the total incoming wave direction at the time of SO swell arrival becomes more pronounced as the relative energetic contribution of these swells decreases. This offset exceeds 45° along the coast of California, whereas it is negligible along the coast of Chile.

For completeness, the individual swell characteristics associated with the events listed in Table 1 are provided in Supplementary Table 2.

### Seasonal variability

The most energetic SO swells do not impact the coast at the same time each year. Figure 2 illustrates the remarkable seasonal variability in the percentage of events affecting the study area. The highest proportion of events is observed during the austral winter (June to August: JJA) at most locations, the only exception being the southern coast of Mexico and Guatemala, where the proportion during the austral autumn (from March to May: MAM) is comparable. Across all the study locations, the proportion of events during the austral winter consistently exceeds one-third of the total, which is likely related to the peak density of extratropical cyclones in the SO during this season^19,20^.

Following JJA, MAM and austral spring (from September to November: SON) present the next highest proportions of events, in that order, with both seasons consistently accounting for more than 20% of the total number of events. Finally, the austral summer (December to February: DJF) features the lowest proportion of events reaching the Pacific coast of the Americas. The number of events during DJF increases closer to the generation area, surpassing 10% at only the southernmost analyzed location.

### Interannual variability in relation to climate modes

Interannual variability is highly pronounced in energetic SO swells. The annual time series of ${H}_{s}$ at the target coastal points clearly reflects this variability (Supplementary Fig. 5). To understand the patterns governing these variations, we analyzed their correlation with two large-scale climate modes characterized by climate indices (see “Methods” section). First, the correlation with the Southern Annular Mode (SAM) was examined via the observation-based Marshall index^58^, which is associated with atmospheric circulation in the SO. Second, the correlation with the ENSO phenomenon was assessed via the multivariate ENSO index (MEI), which influences climate conditions in the Pacific, particularly in the intertropical region.

**Fig. 3:**
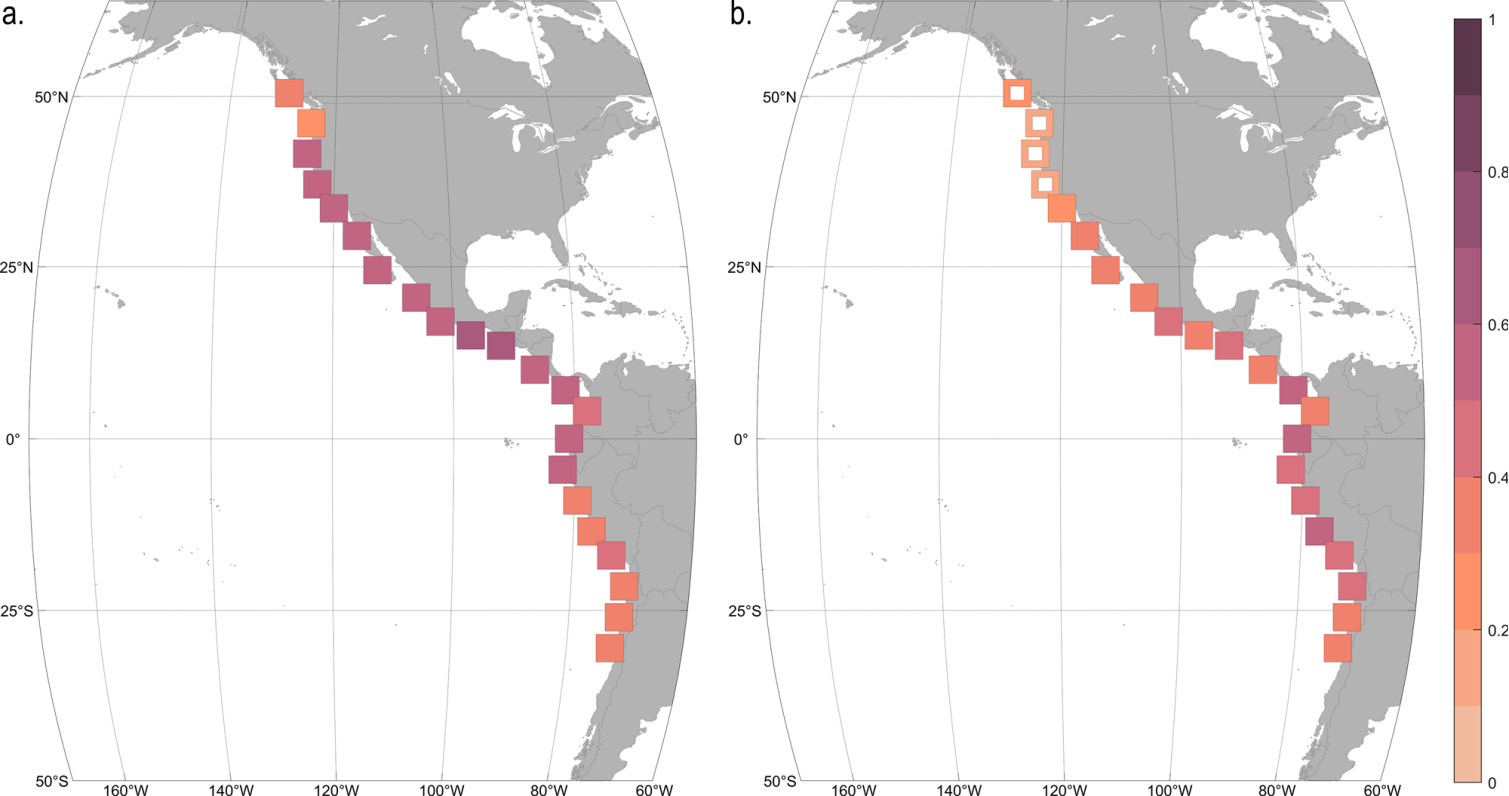
Southern Ocean swells and large-scale climate modes. **a** Linear correlation between the annual mean wave power and the positive phase of SAM (Marshall Index). **b** Linear correlation between the annual mean wave power and the negative phase of ENSO (MEI). Filled squares indicate statistical significance at the 90% confidence level.

The results indicate a strong correlation between the annual mean wave power of the selected events and the positive phase of the SAM, with all analyzed locations showing significant correlations at the 90% confidence level (Fig. 3). During this phase, westerly winds in the SH intensify and shift poleward, along with extratropical cyclones generated in this region^19,59^, which likely contribute to increased wave power in events generated south of 40°S. These findings align with previous studies showing a strong correlation between the positive phase of this index and both mean and extreme wave heights in the SO and eastern Pacific Ocean^60–63^. The observed correlations along the Pacific coast of the Americas are particularly relevant north of the equator, consistently exceeding 0.5. The maximum values are found along the coast of Guatemala and the southernmost coast of Mexico, ranging from 0.6 to 0.7. South of 5°S, the correlation values fall below 0.4 at most locations.

Correlations with the negative phase of ENSO (the Niña phase) were also identified. Correlation values remain below 0.6 across all analyzed locations (Fig. 3), with 82% of them being statistically significant at the 90% confidence level. The most robust results can be observed along the coasts from Peru to Mexico, with values ranging between 0.35 and 0.60. These findings align with previous research^61,62^, indicating a correlation between the negative phase of ENSO and the positive phase of the SAM, leading to increased wave heights in the southeastern Pacific during negative ENSO phases.

Combined models, including ENSO and SAM, were also tested. The additive formulation produced only a minimal improvement over the SAM-only model, while the interaction term was not significant and did not enhance model performance (not shown).

### Historical trends

**Fig. 4:**
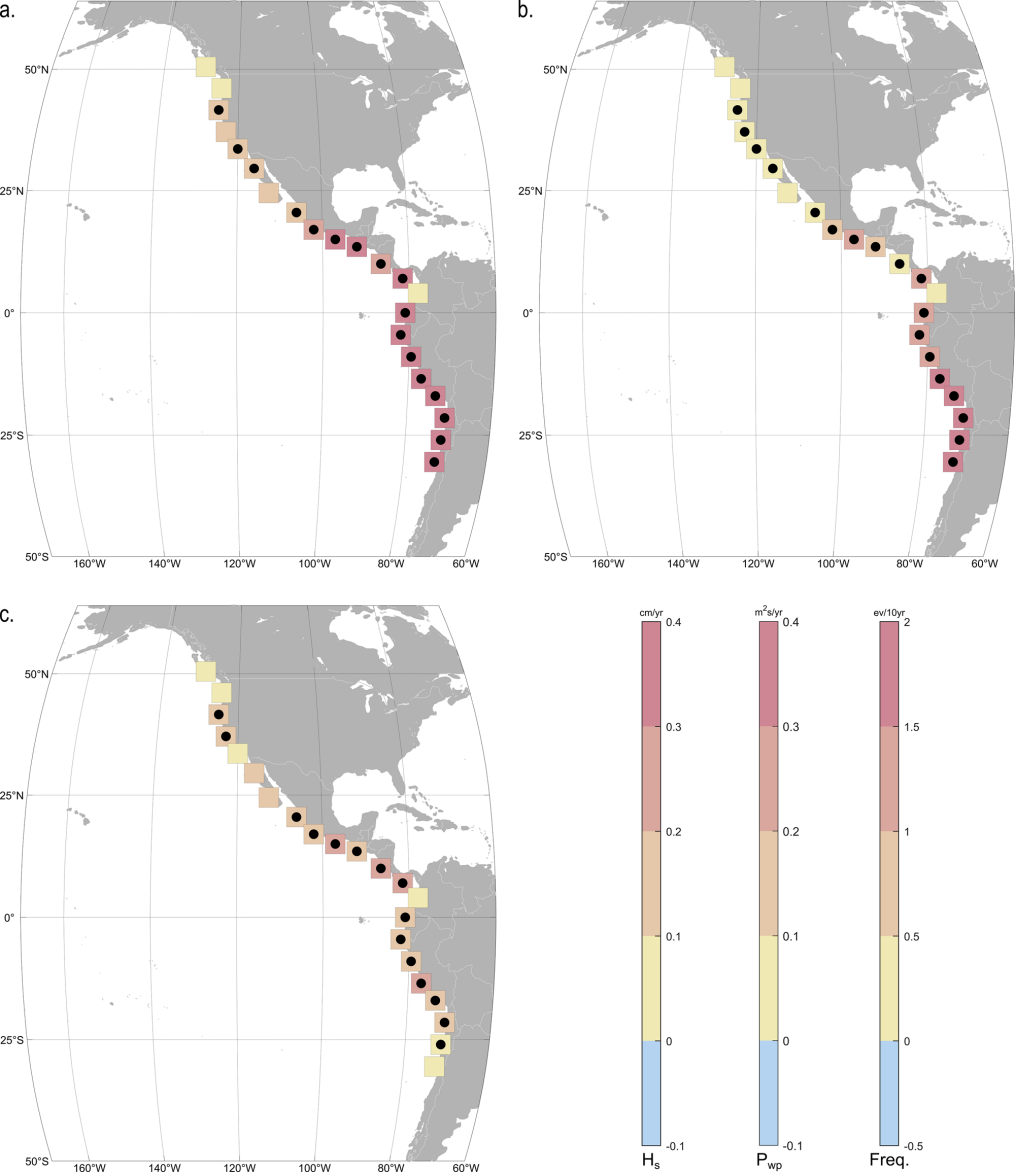
Long-term trends in Southern Ocean swells. Annual trends in significant wave height (**a**) and wave power (**b**). Annual trend in occurrence frequency expressed per decade (**c**). The black dots indicate statistical significance at the 90% confidence level.

Analyzing the long-term evolution of these events provides insight into their potential for future impacts and damage. Over the past 45 years, there has been a significant and general increase in wave height, period, and power, as well as an increase in the occurrence rate of these events (definitions of these indicators are included in the “Methods” section). Figure 4 presents the linear trends obtained, highlighting locations where they are statistically significant at the 90% confidence level (see “Methods” section). A positive trend in wave height for potentially damaging events is observed at all analyzed points, with statistical significance at 78% of the locations. The most pronounced trends occur in South America, where wave heights have increased by more than 0.3 cm/year. Farther north, trends range between 0.1 and 0.4 cm/year in Central America and remain below 0.2 cm/year in North America. In contrast, trends in wave period, although always positive, are smaller and statistically significant at fewer locations, with statistical significance at the 90% level reached at 64% of them (Supplementary Fig. 6).

However, trends in wave power—a combined indicator of wave height and period—exhibit positive values with a statistical significance comparable to that of wave height. This result indicates a sustained increase in wave power over the last decades for the most energetic SO events affecting these coasts throughout the year.

Finally, the occurrence rates of these extreme events were also evaluated. Since the study focuses on extremes based on a fixed number of annual events (20 per year, 900 in total), we assess the trends in event frequency by counting the number of events per year that meet or exceed a specific threshold (see “Methods” section). Although trends were calculated on an annual basis, the results are discussed in decadal terms for clarity.

The key finding is a general increase in  event frequency along the western American coastlines, suggesting that events currently considered potentially hazardous have become more frequent in recent decades. However, regional differences are evident. Along the coasts of South and Central America, trends indicate an increase of 0.5–1.5 events per decade, whereas in North America, the increase remains below 1 event per decade.

### Relative importance of variability scales

The analysis reveals that the characteristics of SO swells vary across different time scales: within a year, between years, and over the long term. Understanding the relative contribution of each time scale to this variability is crucial for designing effective coastal protection measures. To quantify these variations, the variability in wave power across the different time scales was analyzed (see “Methods” section).

Seasonal variability is the dominant time scale influencing wave power variations across the study coast. The magnitude of seasonal variations (normalized by the mean) varies significantly along the Pacific coast of the Americas, ranging from <15% at the northernmost locations analyzed to a maximum of 25–35% along the coasts of South and Central America. Interannual variability ranks second in importance, with magnitudes generally between 10% and 20%, reaching their highest values along the coasts of North America. However, it is important to note that interannual variability itself exhibits a wide range, with the normalized standard deviation consistently exceeding 10% along both the Central American and North American coasts (Supplementary Fig. 7). Finally, the least pronounced variations correspond to long-term trends, which show results comparable in magnitude to interannual variability but remaining below 10% in the northernmost coastal sector.

## Discussion

Swells generated in the SO may pose an important coastal hazard for many regions worldwide. This article provides a detailed analysis of these swell events, with a focus on the Pacific coast of the Americas, based on hindcast wave data spanning 45 years (1979–2023). Using spectral wave data from this database, a methodology is proposed to isolate SO swells from other wave systems reaching the target coasts. Focusing on the most powerful and therefore potentially hazardous events, an analysis of their characteristics and their variability across different time scales is conducted.

Large SO swells are generated at extratropical latitudes in the SH and propagate across ocean basins, reaching as far as the coastal regions of the extratropical Pacific in the NH. The direction of their impact along the coast is determined by their generation area. While they predominantly approach from the southwest, the southern component becomes more pronounced as they travel farther north. These swells reach coastal areas with substantial wave heights and very long periods. The mean ${H}_{s}$ of the 20 most powerful annual events exceeds 2 m along the Pacific coast of South America, ranges between 1 m and 2 m in Central America, and remains above 0.5 m in North America. With respect to the wave period, the ${T}_{p}$ values are generally around 15 s at the time of maximum ${H}_{s}$ along the coast of the study area, with only slight variations between coastal regions. The combination of relatively high wave heights and long periods can make these swells hazardous, as their long periods enhance shoaling and run-up while their typically narrow spectra promote the generation of infragravity waves. Therefore, these events can drive significant water level increases and deliver a substantial amount of energy at the coast, leading to coastal flooding and severe erosion (Table 1). For example, along the Pacific coast of countries such as Mexico and Colombia, authorities issue early warnings to the public when large SO swells are expected, because of their well-documented potential to cause significant damage. Moreover, while this study does not delve into the interactions of these events with other dynamics, it is likely that wave-induced surges may combine with other phenomena, such as sea-level anomalies driven by atmospheric conditions, ENSO-related sea-level variations, and astronomical tides, to trigger extreme flood and erosion events.

While SO swells dominate the wave climate in the SH^16^, their relative importance is greatly reduced in the NH, where North Pacific-generated waves are the dominant wave systems^64,65^. This decrease is evident even when comparing total wave conditions with SO swell characteristics at the time of arrival (Supplementary Fig. 4). The spatial contrast in relative importance influences the regional perception of SO swells and their potential hazards, increasing the likelihood of being overlooked, particularly when they arrive in seasons that do not coincide with those associated with the dominant wave systems. Typically, the wave climate at a given location consists of waves generated in different ocean regions^52,66^. In most cases, one or two primary wave systems are well known and studied, and coastal risk management strategies are typically developed accordingly. However, overlooking potentially hazardous non-dominant systems, such as SO swells, even when they occur outside the climatologically dominant wave season, could have severe consequences. Although appearing as secondary components of the wave climate in certain regions, particularly in the NH, their disregard could lead to an underestimation of coastal risk, primarily due to increased and insufficiently accounted-for exposure. These swells possess substantial energy or may exhibit notable directional deviations from dominant conditions, increasing the potential for significant impacts when their occurrence is not anticipated. This highlights the need for a comprehensive characterization of the wave climate that goes beyond traditional approaches based on integrated parameters, which often mask the presence of remote swells^67^.

This study provides a deeper understanding of when these events are most likely to occur and how their characteristics vary across different time scales. After analyzing the seasonal, interannual, and long-term variations in event power, the seasonal variability is identified as the time scale driving the most intense variations over the 45-year period analyzed. Seasonal changes in event power show normalized mean values reaching 25–35% along the South and Central American coasts. Not only do the characteristics of the events vary throughout the year, but their frequency of occurrence also differs markedly by season. Potentially hazardous SO swells are more likely to occur during austral winter, which coincides with boreal summer, the peak season for coastal recreational use in the NH, increasing exposure of coastal populations and infrastructure to wave impacts. The probability of occurrence also remains high in the austral spring and autumn, with the lowest likelihood during the boreal winter. This seasonal pattern is consistent with previous studies showing that SO swell arrivals in the NH are associated with daily circulation patterns that exhibit a similar seasonality, with a peak during the austral winter, as documented for the California coast^68^.

**Fig. 5:**
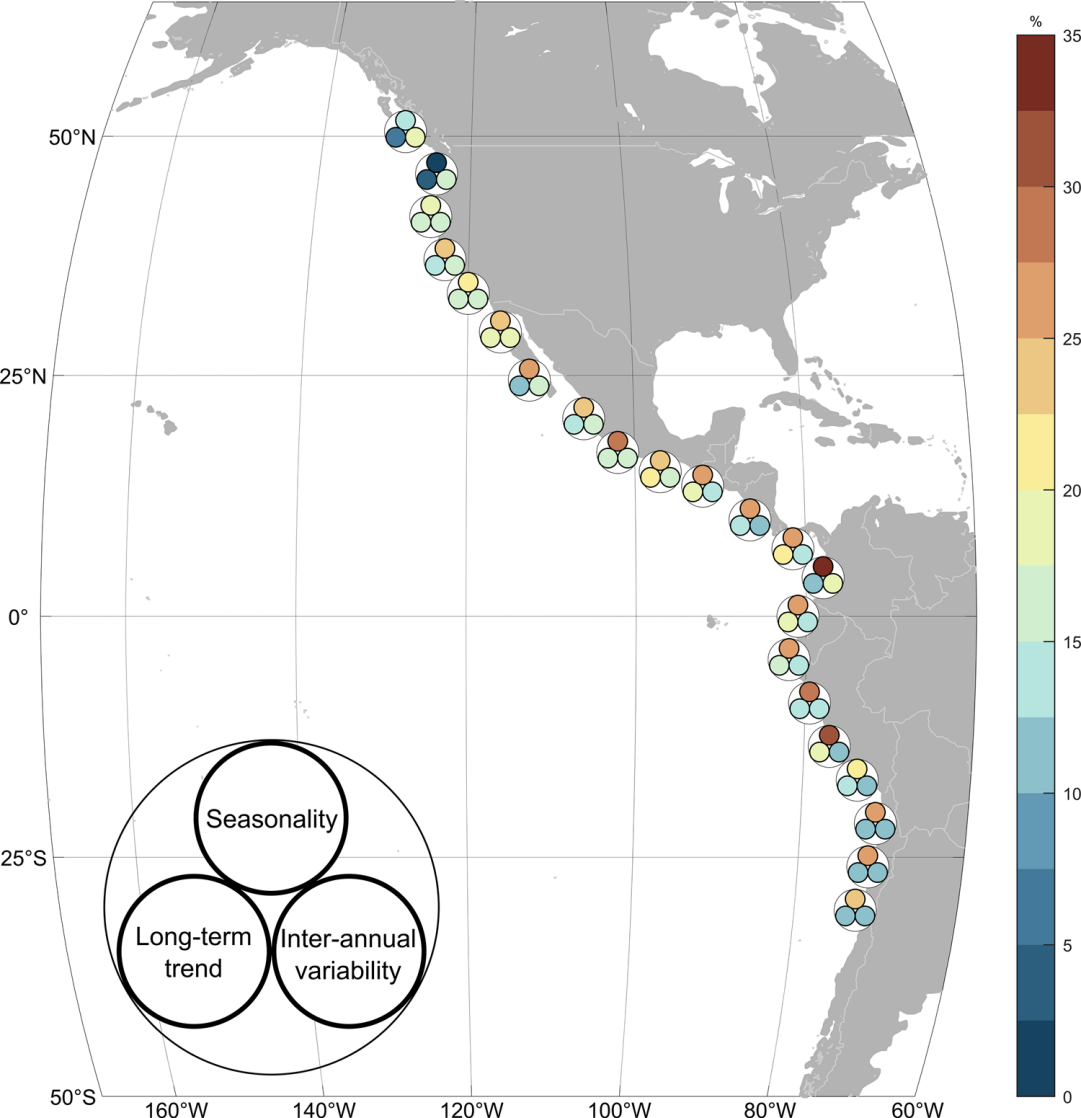
Temporal variability of Southern Ocean swells. Variation in wave power relative to the mean value across seasonal, interannual, and long-term time scales.

Interannual variations also exert a great influence on wave power variations. These interannual variations are closely linked to the influence of the SAM climate mode, with more intense events expected to occur during its positive phase (SAM+). The analysis has revealed that within different years during the study period, the mean relative differences in the annual mean power of these events reached 10–20% in South and Central America and exceeded 15% in North America (Fig. 5), with standard deviations of the same order of magnitude, underscoring the high dispersion of interannual fluctuations in these regions. Therefore, the modulation of these events by large-scale climate modes such as the SAM suggests that even in areas where SO swells are not the dominant system, climate variability can enhance their hazard potential.

Another reason these events require considerable attention is their long-term evolution. Previous studies have assessed the historical and projected trends in wave height in the SO. However, outside the generation regions, studies relying solely on integrated parameters may mask the signals of SO swells^67^. This occurs because the change signal in the integrated parameter combines the contributions of all individual systems. Consequently, along much of the studied coastline, where these swells are often not the dominant system, their change signal is not captured by such studies, making it difficult to assess their past and future behavior. This study takes a step further by examining the historical trend of these swells outside their generation zones. A statistically robust positive historical trend along nearly the entire coast of the Americas has been observed, affecting several indicators, including wave height, period, and power. Considering the origin of these swells, our results are consistent with previous studies based on altimetry^69,70^ and hindcast^71,72^ data, which have reported increases in mean and high-percentile ${H}_{s}$ in the SO. Moreover, the agreement in the sign of change with projected changes in ${H}_{s}$ reported in earlier works^73–76^ in this ocean region suggests that a continued increase in the future is likely. Most importantly, these findings are also in line with prior conclusions drawn from wave spectra in climate projections^23,67^, which showed a robust increase in SO swell energy along all points where these swells propagate, including the west coast of the Americas by the end of the century. These observed increases in SO swell characteristics are consistent with increases in wind speed in the SO over recent decades^70,77^, suggested increases in storm activity^72^, and a positive trend in the SAM in the latter half of the twentieth century^78^. Additionally, while the period of the events also exhibits a general positive trend, its statistical robustness is lower (64% of the analyzed locations). Despite the lack of robustness at some points, likely due to challenges in consistently identifying the peak period of each event and the relatively weak trends in this parameter, the results suggest that wave periods are also increasing along the study coast. Our findings also show that the frequency of energetic SO swells has significantly increased in several regions, which underlines their relevance as a hazard. Future work could extend this analysis to explore how different thresholds of event frequency, from more common to rarer extremes, alter the detected trends in occurrence, thereby providing a more complete picture of evolving coastal hazards.

Although long-term trends represent, in relative terms, the time scale that has contributed the smallest variations in wave power compared with the mean value during the study period, their implications for coastal impacts remain important. The presence of robust long-term trends indicates a growing severity of SO events over recent decades, which is likely to intensify further over time. Moreover, the increasing severity of these swells is compounded by rising mean sea levels^38,39^. As a result, extreme coastal water levels associated with these swells are likely to exhibit an accelerating positive trend due to the interaction of these two dynamics, thereby increasing the hazard for coastal communities and infrastructure.

Notably, all the described variations coexist over time. The fact that long-term variability is lower than interannual variability, for example, does not diminish its importance. This implies that substantial interannual variability, which leads to significant changes from year to year, has occurred against the backdrop of robust long-term increases in SO swells observed over the past 45 years. Consequently, while the magnitude of these events fluctuates markedly annually, these fluctuations occur around increasingly high baseline values. Nonetheless, the design of protective measures to address such events must account for the relative importance of each time scale. The findings of this study can assist in addressing critical aspects, such as identifying the time of year when potentially hazardous SO swell events are more frequent, anticipating interannual variations that may lead to more powerful events, and understanding the intensity of future events likely to occur over long time scales.

This study has several limitations that must be acknowledged. These limitations primarily stem from the methodology used to isolate swells and the reliance on linear theory assumptions in the tracking algorithm, which directly affects the development of the SO swell dataset. This process is influenced by the inherent noise in the spectral partition data. The methods used to obtain these data may struggle to distinguish between wave trains with very similar characteristics, making the subsequent isolation of SO swells more challenging. This issue is particularly notable along coasts closer to the generation areas, where multiple swells with similar characteristics can overlap (e.g., the Chilean coast), leading the partitioning algorithm to interpret it as a single event. In fact, this potential noise may partly explain the low correlation with the SAM index found along the South American coast. Additionally, the identification of event origins is highly sensitive to the linearity of the ${T}_{p}$ fitting, particularly for long travel distances. Several measures were implemented to address these challenges. First, we designed an SO swell-isolation algorithm that accounts for potential noise and seeks the most plausible solution while minimizing the empirical component of its operation. Second, a manual check was incorporated into the algorithm outputs to eliminate potential outliers. Third, the study focuses on the 20 largest events per year, ensuring a sufficient dataset to reduce the influence of unresolved noise and to robustly characterize variability. Finally, the identified events were cross-referenced with events from different sources to ensure that the detected events corresponded to those potentially damaging the coast. Through this rigorous process, a robust dataset was developed, providing a solid foundation for the presented results.

Additionally, it must be acknowledged that the hindcast used in this study, forced with ERA5, has been carefully calibrated and validated against both in situ (buoys) and altimetry data. Altimetry-based validation indicates that the hindcast exhibits small overall errors, with absolute and normalized global metrics comparable to those reported in recent ERA5-based global wave hindcast studies, which typically show global errors on the order of a few tenths of a meter or normalized errors ranging between 10 and 15%, with small biases^79–81^. In addition, a dedicated validation of the SO swell energy reaching the Pacific coast has been conducted using buoy spectral data. Nevertheless, despite the overall satisfactory performance, the use of this atmospheric forcing may still lead to some underestimation of wave conditions associated with extreme winds^80,81^. While this could affect the absolute magnitudes of the events, it should not compromise the main conclusions of this study. The applied methodology, which uses a variable threshold to identify events until a fixed number is reached, ensures that events are consistently detected even if their intensities are slightly underestimated. Together with the consideration of a sufficiently large sample of events each year, it provides robustness against systematic biases, ensuring that the detection of trends, interannual variability, and seasonality remains reliable.

This study demonstrates that the use of spectral data and the isolation of specific wave systems represent crucial next steps in future impact assessments. Spectral wave information, which is becoming increasingly available, allows for a more precise description of the waves impacting particular coastal areas. The use of spectral data helps prevent unnecessary masking of secondary system behaviors, often overlooked in integrated assessments, such as long-period wave energy, which contributes to significant infragravity wave heights, and long-term trends in key wave properties. These data have enabled a detailed and individualized study of large SO swells along the entire coast of the Americas, which would have been challenging to achieve using more traditional approaches on the basis of total integrated parameters.

The findings of this study underscore the current and future importance of SO swell events along the entire Pacific coast of the Americas, as well as their variability. Furthermore, it anticipates that this hazard also poses similar risks in other coastal regions worldwide, affecting both continental margins and low-lying islands, where exposure to SO swells can be especially critical when combined with sea-level rise^48^. This broader perspective highlights that the conclusions drawn here are not limited to the Americas but may be relevant to other regions where SO swells contribute significantly to coastal hazards.

Building on this broader outlook, this study represents an initial step toward a deeper understanding of these events, addressing the analysis from a regional perspective. From this standpoint, there is a significant path forward in two key directions: first, expanding the analysis to a larger scale to identify the global extent of the affected coast; and second, conducting studies at the local scale to investigate the compound effects of these swells combined with other dynamics that pose the highest risk to coastal areas, as well as modeling their resulting impacts.

## Methods

### Hindcast data

This study was conducted using a wave hindcast that covers the 45-year period from 1979 to 2023. The database was generated using the third-generation wave propagation numerical model WaveWatch III v7.00^82^. The model took as input the spatial fields of surface wind and sea ice cover from the ERA5 reanalysis^83^. The wave model was run on an IRI grid system (irregular–regular–irregular, Supplementary Fig. 8). The global grid, with a spatial resolution of 0.5 degrees, covers the global ocean from 67°S to 67°N. Additionally, two irregular grids, with an approximate resolution of 18 km, cover the polar regions. Finally, a global coastal grid with a 0.25° resolution covers the global coastline. Further technical details on the model configuration, numerical settings, and parameterizations are provided in the Supplementary Material.

The global wave hindcast used in this study has been validated with satellite altimeter observations and in situ buoy measurements at the global scale. Details of the validation, including metrics and figures, are provided in the Supplementary Material.

Wave conditions at a given ocean point in time and space can consist of waves generated in various regions under different forcing conditions (i.e., various wave trains coexist). These waves are characterized by different wave directions, heights, and periods. The (total) wave conditions are fully represented by the energy spectrum^5^, which provides a straightforward representation of the wave energy distribution across different period–direction ranges (e.g., between 10 and 12 s and between 180° and 195°; example spectrum in Supplementary Fig. 9). Traditional wave climate studies typically rely on integrated wave parameters^16^. These parameters summarize the average wave conditions of the total wave spectrum into a single value. The following integrated wave parameters were used in this study:

- Significant wave height (${H}_{s}$): This represents the average of the highest one-third of wave heights measured in a one-hour sea state and is equivalent, for practical purposes, to the spectral significant wave height ${H}_{m0}\cong 4\sqrt{{m}_{0}}$, where ${m}_{0}$ is the zeroth moment of the wave spectrum.
- Peak period (${T}_{p}$): This represents the period associated with the maximum spectral energy density within the spectrum.
- Peak direction (${\theta }_{p}$): This represents the direction associated with the maximum spectral energy density within the spectrum.
- Wave energy flux (${P}_{w}$): This represents the energy-weighted average power of the waves.

However, to analyze specific wave systems, i.e., waves with distinct characteristics that consistently recur over time, more detailed information on the wave climate is needed. This study employed spectral partitions to achieve this goal. A spectral partition represents each of the wave trains reaching a specific location at a given time and is identified as one of the spectral peaks within the full wave spectrum^84^. The isolation of each spectral partition and its treatment as an independent spectrum allows for the calculation of integrated wave parameters specific to each partition. For example, if waves arrive from two different generation areas at a given ocean point, two partitions exist, each characterized by its own integrated parameters (e.g., ${H}_{s1}$, ${H}_{s2}$). Throughout the article, total integrated wave parameters refer to values derived from the full wave spectrum, whereas partitioned integrated wave parameters refer to those obtained from individual partitions. All analyses that specifically concern SO swells were based on partitioned integrated wave parameters, whereas total integrated parameters were used only when explicitly stated to assess the relative importance of SO swells within the overall wave climate.

The spectral partitioning scheme used in the employed numerical model is based on an analogy between the surface-elevation spectrum and a topographic surface^85^. In this context, subpeaks and associated surfaces are identified using a digital image watershed partitioning algorithm^86^. The spectral partitions associated with swells are ranked by ${H}_{s}$, with partition 1 having the highest ${H}_{s}$, followed by partition 2, and so forth.

The number of spectral partitions varies across time and space and is influenced by climate variability at different scales (e.g., seasonal, interannual) and by the exposure of the target location to the open ocean (i.e., the possibility of being affected by multiple wave systems propagating from different directions), among other factors. In general, the local wave climate is dominated by one or two swell systems, although additional systems may also exist^52,66^.

Here, hourly time series of the total integrated wave parameters ${H}_{s}$, ${T}_{p}$, and ${\theta }_{p}$ were used alongside hourly time series of spectral partitions. In particular, six spectral partitions were considered: five corresponding to swell systems (partitions 1 to 5), whereas the remaining partition (partition 0) was related to wind-sea waves. For each spectral partition, the partitioned integrated wave parameters ${H}_{s}$, ${T}_{p}$, and ${\theta }_{p}$ were stored.

For validation against instrumental data, directional spectra with three-hourly temporal resolution were utilized. These spectra were discretized into 32 frequency bins, exponentially distributed from 0.0373 to 0.7159 Hz (i.e., 1.4–26.8 s) and divided into 24 directional sectors of 15° each.

Wave parameters were extracted at 22 coastal target points (Supplementary Fig. 1). Most locations are situated at depths sufficient to avoid substantial shoaling effects for the dominant wave periods considered (Table SM1). Two points (P2 and P17), however, are located at shallower depths where some limited effects may occur, particularly for longer-period waves. Importantly, the inclusion of these nodes does not affect the regional-scale patterns discussed in this study.

### Isolation of swells

The approach for isolating swells is based on previous works^53–55^. Unlike space–time tracking methods developed to reconstruct basin-scale swell fields^87^, it is applied here to identify and characterize swell events directly from time series at individual coastal locations. According to linear wave theory, the travel time and distance of swells from their generation area can be calculated by analyzing the variation in the ${T}_{p}$ parameter over time at the target location. Thus, swells were identified through an analysis of the ${T}_{p}$ time series by detecting a constant descending slope. This descending slope was then fitted to a straight line to determine the point of generation. The distance traveled by the swell since its generation can be calculated as:

1 $$d=\,\frac{g}{4\pi }\frac{1}{m}$$

where *m* is the slope of the peak frequency and *g* is the gravitational acceleration.

This tracking algorithm has been previously applied using total integrated wave parameters. However, in this study, it was adapted for use with partitioned integrated parameters. Owing to the method used to extract spectral partitions from the model, a single swell event may not always be represented by the same partition (i.e., Partition 1, Partition 2), as the rank of its associated ${H}_{s}$ may vary between consecutive time steps. Therefore, all available partitions were used to identify independent swell events accurately. Supplementary Fig. 2 presents an example for point P10, which shows the partitioned ${T}_{p}$ time series for the six spectral partitions, i.e., ${T}_{p0}$, ${T}_{p1}$…, ${T}_{p5}$. In this figure, the selected swell event is highlighted in orange to distinguish it from overlapping swells occurring before and after. As shown in Supplementary Fig. 2, the use of spectral partitions allows for the identification of overlapping events.

The algorithm follows these steps:

1. Directional filtering: Partitioned data for the first analyzed year are filtered to ensure that swell events originate south of 40°S, selecting only those following great circle paths to the target coastal location.
2. Construction of a single time series: A single ${T}_{p}$ time series is generated by selecting the maximum partitioned ${T}_{p}$ at each time step from all partitions that meet the directional criterion from *step 1*.
3. Peak Over Threshold (POT) method: The POT method^88^ is applied to the ${T}_{p}$ time series from *step 2*, using a threshold that results in 100 exceedance peaks. The choice of 100 events was made to ensure a sufficiently large sample and to avoid potential issues of the algorithm in reliably tracking swells, especially during the early stages of a swell, when they may not be continuously defined due to their low energy. This criterion ensures that the 20 most energetic events per year are consistently captured.
4. Event identification: Starting from each peak, the algorithm iteratively selects the most likely next points on the basis of a weighted Euclidean distance in ${T}_{p}$-${H}_{s}$-${time}$, with constraints on changes in ${T}_{p}$, ${H}_{s}$, and ${\theta }_{p}$ to ensure physical continuity. For clarity, steps 1–4 are illustrated in Supplementary Fig. 14.
5. Parameter evolution and anomaly checks: Events are refined by evaluating the evolution of the parameters and anomalies, truncating events with sustained increases or excessive slopes in ${T}_{p}$, or with overlapping peaks or abrupt changes in ${H}_{s}$.
6. Event origin calculation: The origin coordinates are estimated by fitting a line from the event’s start to either the maximum ${H}_{s}$, minimum ${T}_{p}$, or the event’s end, whichever occurs first, provided that the selected event period lasts at least 48 h to ensure a robust fit. The origin uncertainty is assessed through confidence intervals of the fitted slope. If the range of calculated origins falls south of 40°S, the event is saved along with all its characteristics.
7. Removal of selected events: The data selected for the event, regardless of whether it meets the latitudinal criterion, are removed from the partitioned ${T}_{p}$ time series to prevent reuse in subsequent steps.
8. Discarding invalid points or events: If, in *step 3*, a point becomes isolated and no valid event can be selected from it, the initial point is discarded from the partitioned ${T}_{p}$ time series to prevent reselection, and the algorithm moves on to the next event. Similarly, if an event found does not meet the minimum duration required according to *step 6*, its initial point is discarded.
9. Threshold readjustment: After the 100 possible events are analyzed, a number equal to or less than 100 will meet the selection criteria. If necessary, the algorithm revisits *step 2*, adjusting the threshold until the number of exceedances matches the remaining number of events.
10. Steps 3–9 are repeated until 100 events meeting the conditions are found or until no further events remain for evaluation.
11. The process then moves to the next year, where the procedure is repeated.

The full details of the algorithm steps can be found in the Supplementary Material.

The designed algorithm incorporates a significant empirical component. Therefore, the results for the 22 analyzed coastal points were manually reviewed, discarding any outliers.

Each selected swell event was characterized by its wave characteristics (${H}_{s}$, ${T}_{p}$, and ${\theta }_{p}$) at the moment of maximum spectral energy density, i.e., corresponding to the maximum ${H}_{s}$.

### Validation of Southern Ocean swell energy

To validate the incoming wave energy propagating from the SO in the modeled dataset, observations from five spectral buoys managed by the Coastal Data Information Program (CDIP) and four spectral buoys managed by the National Data Buoy Center (NDBC) were used. For each buoy, the nearest hindcast grid point providing directional spectra was selected for comparison. All the selected buoys are located at depths sufficiently large to avoid wave-breaking related shallow-water processes. Supplementary Table 3 summarizes the key characteristics of the buoys.

For comparison, wave energy propagating in a direction within the directional range connecting the buoy locations to the SO via great circle paths was selected. Additionally, only energy with periods longer than 15 s was considered. Comparisons were made on the basis of the equivalent$\,{H}_{s}$ (${H}_{s}^{e}$), which was calculated as follows:

2 $$\,{H}_{s}^{e}\cong \,4\sqrt{{m}_{0\alpha f}}$$

where ${m}_{0\alpha f}$ is the zeroth-order moment of the wave energy spectrum within a specified directional range $\alpha$ and frequency range *f*.

This procedure was applied identically to both the modeled and observed data, yielding two consistent datasets with daily ${H}_{s}^{e}$.

Furthermore, the previously described swell-isolation algorithm was applied to buoy locations to identify the most energetic events, enabling a more precise comparison of modeled vs. observed key events.

### Swell indicators

To analyze swell events from the SO, a series of related indicators was used, all of which were computed independently at each coastal target point, both to study the properties of the events and to assess their variability at different time scales.

Although swell events have a specific duration during which their properties change, they were characterized here by their properties at the moment the event ${H}_{s}$ reaches its peak. At this instant, the values of ${H}_{s}$, ${T}_{p}$, and ${\theta }_{p}$ of the events were extracted. This information was then used to calculate the following indicators:

- Wave height: The mean ${H}_{s}$ of the events that reach the target point with the greatest power was calculated. This indicator was computed both on an annual basis and for the entire study period.
  For the annual calculation, the mean of the 20 most powerful events in each year was considered. For the total calculation, the mean was derived from the 900 recorded events, which were based on the 20 most powerful events per year over a 45-year period. The sensitivity was analyzed using sample sizes of 10 and 50 events, and consistent results were obtained across all cases, supporting the robustness of the selected 20 events.
  Although wave power is typically formulated using the mean wave energy period^15^, this parameter was not available in the dataset used. Therefore, a proxy for wave power was calculated using the peak period as follows:
  3 $${P}_{{wp}}={{H}_{s}}^{2}{T}_{p}$$
  Importantly, in the case of wave partitions, particularly for the study of mature swells, the difference between the mean period and the peak period is relatively small, as these swells concentrate most of their spectral energy density within a narrow range of periods.
- Wave period: Same as for wave height but the ${T}_{p}$ parameter is used instead.
- Wave direction: Same as for wave height but the ${\theta }_{p}$ parameter is used instead.
- Wave power: Same as for wave height but the ${P}_{{wp}}$ parameter is used instead.

- Frequency of occurrence: This indicator represents the number of events – out of the 20 events selected per year at each point – that meet or exceed a certain threshold. To ensure that the threshold was adaptable to varying wave conditions across the study locations, it was defined as the highest value of the 20^th^-ranked annual event by power over the 45-year period analyzed. This definition (i) anchors the indicator to a fixed, high-severity level that remains comparable across years and locations, and (ii) guarantees the full 0–20 dynamic range in annual exceedance counts, avoiding both saturation (too many exceedances if the threshold were set lower) and emptiness (too few exceedances if set higher). By construction, the year that sets the threshold attains 20 exceedances, whereas all other years are ≤20.

### Interannual variability

The correlation between changes in wave power and different climate modes was calculated using linear regression models (Eq. 4), which in the univariate case are mathematically equivalent to Pearson’s linear correlation^89^. Annual indices were used to explore interannual variability through aggregated annual metrics. For SAM, we used the annual observation-based Marshall index^58^. For ENSO, we obtained annual values by averaging the monthly MEI index using an ENSO-year definition (May–April)^90–93^, ensuring that the full evolution of individual ENSO episodes (El Niño/La Niña) is contained within a single year, while keeping the main wave season (JJA) intact and not split across two different years.

The regression models were then fitted between these indices and the annual mean ${P}_{{wp}}$ of the 20 most powerful events at each target point.

4 $${P}_{y}=\alpha+\beta {X}_{y}+{\varepsilon }_{y}$$

where ${P}_{y}$ is the annual mean ${P}_{{wp}}$ of the 20 most powerful events, ${X}_{y}$ is the annual index, $\alpha$ is the intercept, $\beta$ is the slope, and ${\varepsilon }_{y}$ is the residual.

### Trend calculation

Trends in the different indicators were estimated using the non-parametric Sen’s slope estimator to minimize the influence of outliers on the results. The statistical significance of the trend was assessed with the Mann–Kendall trend test (MK)^94,95^, which evaluates the presence of a monotonic upward or downward trend over time by testing if the slope of the estimated regression line differs from zero. The Sen–MK combination has been widely applied in the climate science literature, as it provides a robust, distribution-free framework for detecting and quantifying monotonic trends in environmental and climatic time series^71,96,97^ where the assumptions of parametric methods are often not met. A trend was considered statistically robust when MK indicated significance at  the 0.1 level, a threshold commonly used in climate studies^96,98–100^.

### Quantification of climate variability

Climate variability was assessed by analyzing the maximum relative variation in ${P}_{{wp}}$ at different temporal scales.

Seasonal variability was computed as the mean of the relative difference between the maximum and minimum seasonal means ${P}_{{wp}}$ for the study period across the four seasons, normalized by the study period’s mean value. Seasonal means were calculated by grouping all identified events according to the season in which they occurred and averaging ${P}_{{wp}}$ across events within each season.

Interannual variability was calculated as the Gini mean difference (GMD)^101^ of annual mean ${P}_{{wp}}$ values over the 45-year detrended period, normalized by the study period’s mean value. The GMD corresponds to the average of all pairwise absolute differences among annual means, providing a representative measure of typical interannual fluctuations. Additionally, the normalized standard deviation of all pairwise absolute differences was computed as an indicator of the variability that may be introduced by interannual fluctuations.

Trend-associated variability was determined as the difference between the trend-fitted values of annual mean ${P}_{{wp}}$ in the final year and the first year of the 45-year period, normalized by the study period’s mean value.

## Supplementary information

Supplementary Information

Transparent Peer Review file

## Data Availability

The source data underlying the figures generated in this study have been deposited in the Zenodo repository 10.5281/zenodo.18954278. The hourly integrated sea state parameters and wave spectra from the wave hindcast dataset used in this study are available under restricted access due to institutional data management policies. Access to these data for research purposes can be requested by contacting ihdata@ihcantabria.com. The buoy observations used to validate the Southern Ocean swell signals were obtained from the Coastal Data Information Program (CDIP; https://cdip.ucsd.edu/m/) and the National Data Buoy Center (NDBC, NOAA; https://www.ndbc.noaa.gov/).

## Code availability

All analyses were conducted using MATLAB R2023b (academic license). The scripts developed for the analyses are available from the corresponding author upon reasonable request.
