# Supplementary material for: Four decades of intensifying Southern Ocean swells along the Pacific coast of the Americas
Source: Nat Commun. 2026 Apr 29;17:5860. doi: 10.1038/s41467-026-71813-1 (PMC13333821; doi:10.1038/s41467-026-71813-1)
Supplement: Supplementary file 1 — Supplementary Information [file 41467_2026_71813_MOESM1_ESM.pdf]

Supplementary Material: Four decades of intensifying Southern Ocean swells along the Pacific coast of the Americas

Hector Lobeto[1*], Melisa Menendez[1], Alvaro Semedo[2], Iñigo J. Losada[1], Gil Lemos[3]

[1]*IHCantabria - Instituto de Hidráulica Ambiental de la Universidad de Cantabria, Santander, Spain* (lobetoh@unican.es)[*]
[2]*Department of Coastal and Urban Risk and Resilience, IHE Delft Institute for Water Education, Delft, The Netherlands*
[3]*Instituto Dom Luiz (IDL), Faculdade de Ciências, Universidade de Lisboa, Lisbon, Portugal*

Methodology

Swell identification algorithm

The algorithm follows these steps:

1. Directional filtering: Partitioned data are filtered ensuring their origin is in the SO south of 40º. To that end, the minimum and maximum directions a swell event can have are calculated, ensuring the swell follow great circle paths from a source location in the SO south of 40ºS to the target coastal location.
2. Construction of a single time series: A single T_p time series is generated from all partitioned T_p values that meet the directional criterion from *step 1*. The maximum available partitioned T_p value for all partitions is selected at each time step.
3. Peak Over Threshold (POT) method: The POT method[1] is applied to the single T_p time series obtained in *step 2* for the first year using a threshold that results in 100 exceedance peaks.
4. Event identification: Each selected peak is used as the starting point for the algorithm. The algorithm identifies the next most probable data point from all available options, step by step, defining the descending T_p line that characterizes the swell event. The next most likely data point is sought within the next 8 hours, as discontinuities may occur. The next point is selected using a weighted Euclidean distance approach as the closest one in a three-dimensional T_p-H_s-$time$ space. Distances are calculated using normalized H_s and T_p variables and time in hours. The weight assigned to the T_p variable is 1 in all cases, while the weight for the H_s variable is always 0.1 (its purpose is simply to distinguish the closest cases). Lastly, the weight associated with the time variable is determined as the linear slope formed by the previously selected points (with a minimum weight value of 0.01). The main goal is to assign equal weight to the time and T_p variables. This means that if the slope is steeper, the weight assigned to time will be greater, and vice versa.

   $$d = F_1 \cdot T_p + F_2 \cdot H_s + F_3 \cdot time \qquad \text{Eq. SM1}$$

   where F_1 = 1, F_2 = 0.1 and F_3= [0.01, Inf].

   Additional restrictions imposed are:
   i. T_p difference between two consecutive data points cannot exceed 2 seconds per hour or 12 seconds in total if the next point has a lower T_p, or 0.25 seconds per hour if the next point has a higher T_p.
   ii. H_s difference between two consecutive data points cannot exceed 0.8 meters per hour.
   iii. θ_p difference between two consecutive data points cannot exceed 15 degrees per hour.

   To ensure continuity, once the next point is found, the algorithm is applied in reverse, ensuring continuity only if the reverse-selected point matches the original point or a point already selected.
5. Parameter evolution and anomaly checks: Once the event is isolated, the algorithm evaluates the continuity of the T_p and H_s time series, thus avoiding abrupt jumps or anomalous behaviors. The

objective is to ensure the restrictions remain sufficiently flexible, addressing only clearly identifiable anomalous cases. First, if trends in T_p show an increasing trend for more than 12 hours, the event is truncated at the start of this increase. Second, if as a result of the merging of events, an event nearing its end may overlap with another, resulting in two wave height peaks, the first peak will be selected. Third, if abrupt jumps in H_s are detected, the event is truncated when hourly relative slopes exceed 100%. Finally, we analyze the slope of the peak period, and if the slope exceeds 60 s/h and is greater than the mean slope plus 10 standard deviations, the event is truncated.

6. Event origin calculation: The coordinates of the event's origin are calculated using Eq. 1. The fitted process involves drawing a line from the event's start to either the instant of maximum H_s, the instant of minimum T_p or the event's end, whichever occurs first. To ensure a reliable fit, the event must last for at least 48 hours. Since the linear fit may not always be perfect, some uncertainty in the slope estimation is expected. To address this, 95% confidence intervals for the slope have been calculated, and the corresponding event origins associated with the extremes values of this interval have been determined. If the range of calculated origins falls south of 40ºS, the event is saved along with all its characteristics.
7. Removal of selected events: The data selected for the event, regardless of whether it meets the latitudinal criterion, are removed from the partitioned T_p time series to prevent reuse in subsequent steps.
8. Discarding invalid points or events: If, in *step 3*, a point becomes isolated and the next point is not found, the initial point of that event is discarded from the partitioned T_p time series to prevent re-selection. The algorithm moves on to the next event. Similarly, if an event found does not meet the minimum duration required according to *step 6*, its initial point is discarded.
9. Threshold readjustment: After analyzing the 100 possible events, a number equal to or less than 100 will meet the selection criteria. If necessary, the algorithm revisits *step 2*, adjusting the threshold until the number of exceedances matches the remaining number of events.
10. Steps 3–9 are repeated until 100 events meeting the conditions are found or until no further events remain for evaluation.
11. The process then moves to the next year, where the procedure is repeated.

Hindcast data

Model configuration

The wave hindcast was developed with the third-generation spectral wave model WaveWatch III (WW3)[2], version 7.00. The spectral discretization includes 24 directions and 32 nonlinearly spaced frequencies between 0.0373 Hz and 0.7159 Hz, with a frequency increment factor of 1.1. Bathymetry, land–sea masks, and obstruction grids were generated with the WW3 preprocessing tools[3]. The model was forced with spatially varying surface winds and sea-ice concentration from the ERA5 reanalysis[4].

A three-domain multi-grid configuration was implemented following the Irregular–Regular–Irregular (IRI) scheme[5], designed to enhance model skill at high latitudes while maintaining global coverage (Supplementary Table 3, Supplementary Figure 8). It consists of (i) a global regular grid at 0.5° resolution, (ii) an Arctic curvilinear polar stereographic grid at 18 km resolution, and (iii) an Antarctic curvilinear polar stereographic grid at 18 km resolution.

The physical source terms and numerical schemes were configured as follows:

- Wind input and dissipation: ST4 physics[6] with parameterization T471. Default parameter values were retained, except for the nondimensional wind-wave growth parameter (βmax), which was tuned during calibration.
- Nonlinear interactions: Discrete Interaction Approximation (DIA)[7].
- Bottom friction: SHOWEX formulation[8].
- Depth-induced breaking: Shallow water depth breaking [9] with the Miche-style limiter.
- Reflection: shoreline and sub-grid reflection coefficients set to 0.05 and 0.1, respectively.

- Propagation: Ultimate Quickest third-order scheme with the garden sprinkler effect reduction[2].
- Sea-ice effects: wave–ice dissipation and scattering terms (IC2 and IS2)[10], with a constant ice thickness of 1 m[11].

Model calibration

The calibration focused on the nondimensional wind–wave growth parameter (βmax), which strongly influences the energy input from winds into waves. All other source-term parameters were kept at their default values. βmax was tuned to account for biases in ERA5 winds, following the methodology of Stopa (2018)[12], which uses de-noised satellite altimeter observations to optimize wave model performance. Specifically, βmax was adjusted to minimize the global bias in H_s by matching the 10th, 25th, 50th, 75th, 90th, and 99th percentiles of the observed and simulated H_s distributions.

The calibration was conducted with a 1-year hindcast to avoid overfitting to a specific season or month[11,12]. The year 2010 was selected because of the large amount of altimeter data available (Ardhuin et al., 2019). During this year, five altimeter platforms were operational: ERS-2, Jason-1, Jason-2, Envisat, and CryoSat-2.

The model was run six times using βmax values of 1.33, 1.43, 1.46, 1.50, 1.55, and 1.65. For each simulation, modelled H_s fields were co-located with the altimeter tracks by applying linear interpolation in time and bilinear interpolation in space. The resulting data pairs were aggregated into 0.5° grid cells, from which several error metrics were computed, including bias, root mean square error (RMSE), scatter index (SI), and Pearson correlation (R). Altimeter measurements within 20 km of the coast were excluded due to known retrieval errors caused by coastal backscatter[13–15].

To determine the optimum βmax, percentile-based residuals between observed and simulated H_s were calculated. The optimal value corresponded to the simulation in which these residuals were minimized across percentiles. Results indicate that βmax = 1.46 provides the best overall performance, with residuals within ±0.1 m across percentiles. The optimum run (βmax = 1.46) shows a bias of 0.015 m, RMSE of 0.323 m, SI of 0.105, and correlation of 0.971.

Model validation

The wave hindcast was validated against both in situ buoy records and satellite altimeter observations.

Buoy Validation. Modelled sea-state parameters (H_s; mean period, T_{m02}; peak period, T_p; mean direction, Dir_m) were compared with buoy measurements using standard error metrics (bias, RMSE and R). Validation was carried out using buoys. Buoys were selected based on a minimum distance of 25 km from the coast, a minimum temporal coverage of 10 years, and a maximum time gap of 40%. No buoy in the Southern Hemisphere satisfied these criteria; therefore, the requirements were relaxed in order to identify buoys in the South Pacific region. Specifically, the temporal coverage threshold was reduced to 8 years and the maximum gap increased to 50%, which allowed the inclusion of two buoys in the Southern Hemisphere. Directional records (Dir_m) were available for only 10 of the 29 buoys considered.

Results are summarized in Supplementary Table 4 and illustrated in Supplementary Figure 10 (Bias), Supplementary Figure 11 (RMSE), and Supplementary Figure 12 (Correlation).

Altimeter validation. Model H_s was also evaluated against an inter-calibrated multi-mission altimeter dataset spanning 1991–2018. This provided a 27-year record of de-noised, recalibrated observations (see Section 2.2.1.2). Globally, the hindcast shows negligible bias, with an RMSE of 0.294 m, SI of 0.098, and correlation of 0.976. Supplementary Figure 13 presents the validation results for the Pacific Ocean, where correlations generally exceed 0.90 (and >0.95 in the North Pacific and Southern Ocean), while slightly lower values (~0.8) occur in equatorial regions. Biases are negligible (<0.05 m) over most of the Pacific, especially along the eastern basin. RMSEs range from ~0.20 m in the equatorial zone to 0.4–0.5 m at high latitudes near Antarctica.

Tables

Point	Longitude [º]	Latitude [º]	Depth [m]
P1	-129.0	50.5	1541.1
P2	-124.5	46.0	207.9
P3	-125.0	41.5	1786.6
P4	-123.0	37.0	1906.0
P5	-120.0	33.5	692.2
P6	-116.0	29.5	1717.0
P7	-112.5	24.5	630.1
P8	-106.0	20.5	2074.9
P9	-102.0	17.0	3863.3
P10	-97.0	15.0	3929.4
P11	-92.0	13.5	2752.8
P12	-86.5	10.0	3846.0
P13	-81.5	7.0	1047.6
P14	-78.0	4.0	1748.6
P15	-81.0	0.0	2308.1
P16	-82.0	-4.5	4511.2
P17	-79.5	-9.0	135.8
P18	-77.0	-13.5	1023.4
P19	-73.5	-17.0	3600.9
P20	-71.0	-21.5	5308.9
P21	-71.5	-26.0	6040.2
P22	-72.5	-30.5	5516.2

Supplementary Table 1 | Characteristics of the study locations. Columns from left to right: identifier, longitude, latitude, and depth.

Ev	H_s [m]	T_p [s]	θ_p [º]	Point
1	1.77	17.90	191.90	P12
2	3.89	16.47	220.50	P20
3	3.87	17.57	217.90	P20
4	2.45	16.99	207.50	P11
5	2.60	17.90	198.30	P11
6	2.78	17.88	217.20	P18
7	2.23	16.26	202.10	P10
8	1.34	18.02	202.60	P5
9	4.09	17.71	227.20	P20
10	2.23	17.96	226.80	P17
11	3.76	17.94	215.80	P20
12	2.74	18.02	204.50	P11
13	2.37	16.68	207.70	P11
14	2.11	18.10	180.20	P9
15	1.86	17.87	209.30	P10
16	1.98	15.44	195.90	P10
17	1.94	15.14	196.20	P10
18	2.28	16.47	194.10	P11
19	2.09	15.46	204.10	P11
20	1.83	16.04	206.20	P13

Supplementary Table 2 | Southern Ocean swell conditions during selected events. Events correspond to those listed in Table 1 of the main text, evaluated at the locations indicated therein. The table reports H_s, T_p and θ_p at the time of maximum H_s.

ID	Longitude [º]	Latitude [º]	Depth [m]	Period
CDIP 067	-119.892	33.226	315	Sept 1991 – Dec 2022
CDIP 222	-121.497	34.767	650	Mar 2016 – Dec 2022
CDIP 029	-123.465	37.940	600	Dec 2016 – Dec 2022
CDIP 157	-122.104	36.335	369	Sep 2017 – Dec 2022
CDIP 071	-120.780	34.452	550	Oct 1991 – Dec 2022
NDBC 32012	-85.078	19.425	4534	Dec 2007 – Jan 2018
NDBC 43010	-125.032	10.051	4769	Sep 2016 – Nov 2017
NDBC 51004	-152.186	17.496	5297	Sep 2009 – Dec 2022
NDBC 51028	-153.913	0.000	4747	Jan 2005 – Apr 2008

Supplementary Table 3 | Main characteristics of the spectral buoys used to validate Southern Ocean wave energy.

Domain	Grid type	Spatial resolution	Geographical limits		Overlapping latitudes with the parent grid
			longitude	latitude	
Global	Regular	0.5ºx0.5º	180ºW-180ºE	67ºS-67ºN	-
Arctic	Irregular	18 km x 18 km	180ºW-180ºE	63ºN-90ºN	63ºN-67ºN
Antarctic	Irregular	18 km x 18 km	180ºW-180ºE	63ºS-90ºS	63ºS-67ºS

Supplementary Table 4 | Simulation domains characteristics.

H_s				T_{m02}			T_p			Dir_m
Bias [m]	RMSE [m]	R	P95 Bias [m]	Bias [s]	RMSE [s]	R	Bias [s]	RMSE [s]	R	Bias [°]
**-0.10**	0.37	0.94	-0.39	-0.64	0.91	0.89	0.31	2.54	0.61	-1.49

Supplementary Table 5 | Validation metrics of the wave hindcast against in situ wave buoy observations. Metrics are reported for H_s, T_{m02} and Dir_m for both global and regional domains. 'P95 Bias' refers to the bias in the 95th percentile of the variable.

Figures

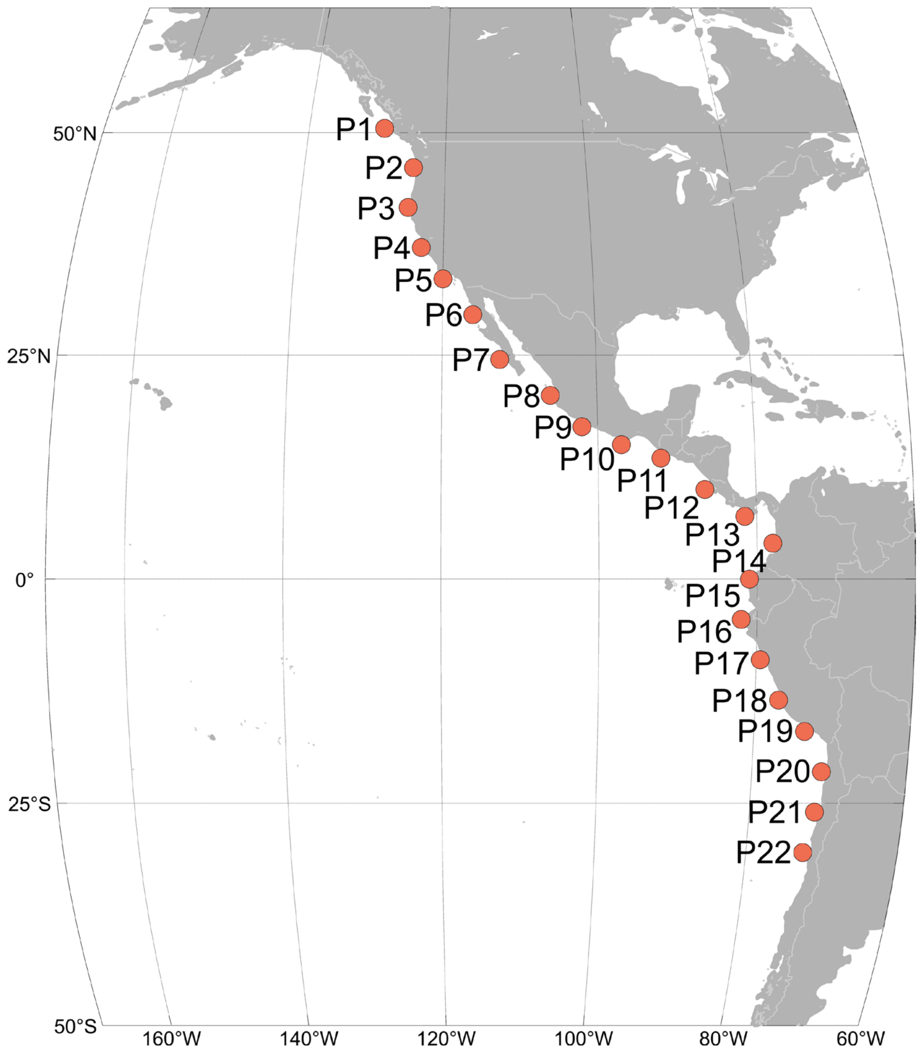

Supplementary Figure 1 | Location of the target coastal points.

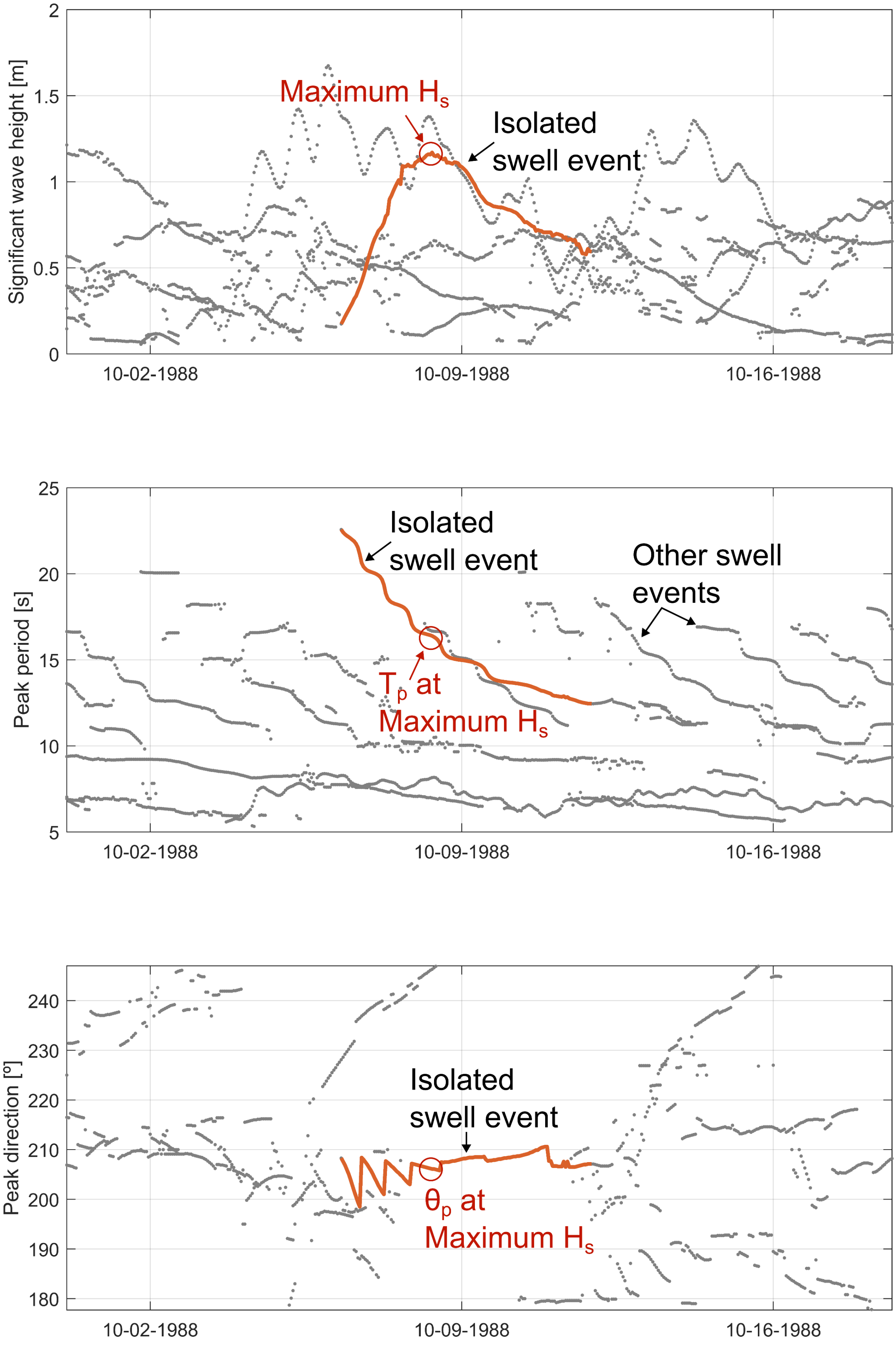

Supplementary Figure 2 | Example of an isolated swell event within the spectral partition time series. Gray dots represent the combined partitioned data from the six partitions considered in the study. The event is highlighted in orange. The top panel shows the significant wave height, the middle panel shows the peak period, and the bottom panel displays the peak direction. The maximum significant wave height of the event is highlighted in red, along with the associated peak period and peak direction.

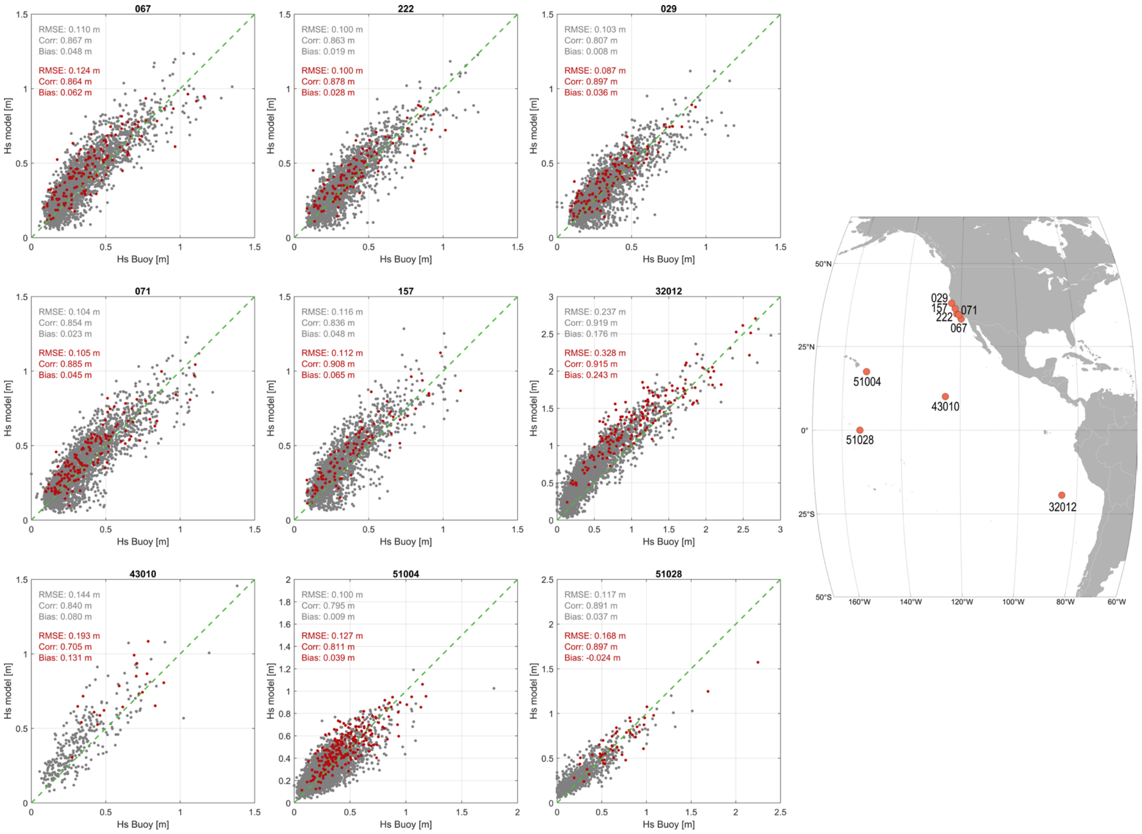

Supplementary Figure 3 | Validation of Southern Ocean swell energy against buoy observations. Gray dots show the daily equivalent significant wave height (H_s^e; Eq. 2, main text) associated with Southern Ocean energy. Red dots show the daily equivalent significant wave height recorded during the selected Southern Ocean swell events at the buoy locations. Buoy locations are shown in the map.

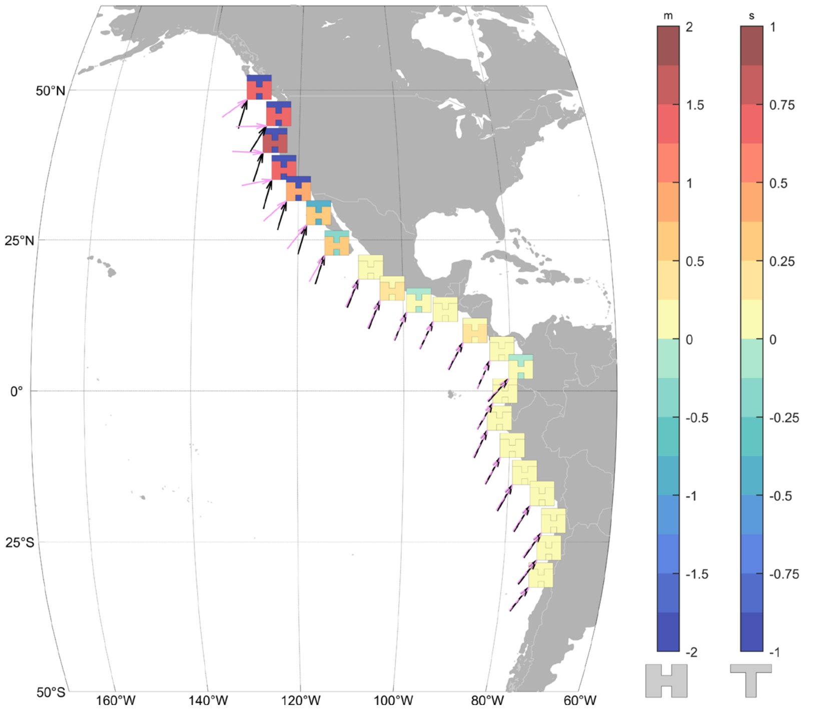

Supplementary Figure 4 | Differences between Southern Ocean swell events and total wave conditions at arrival. Differences in the mean significant wave height (H within the square), peak period (T within the square), and peak direction (arrows) of the SO swell events compared to the overall conditions at the time of the arrival of the events, computed as the total wave parameters minus the SO swell–associated parameters. Pink arrows indicate the directions associated with total wave conditions; black arrows indicate the directions associated with SO swells.

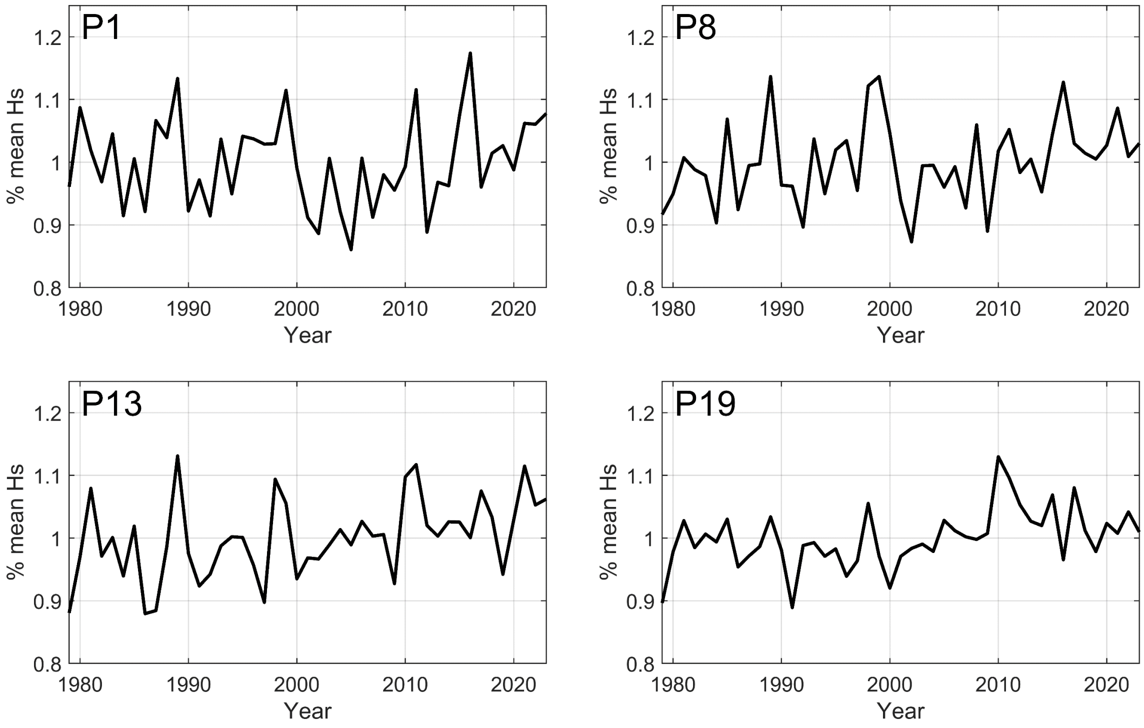

Supplementary Figure 5 | Interannual variation of the annual mean significant wave height. The wave height is normalized with respect to the mean wave height.

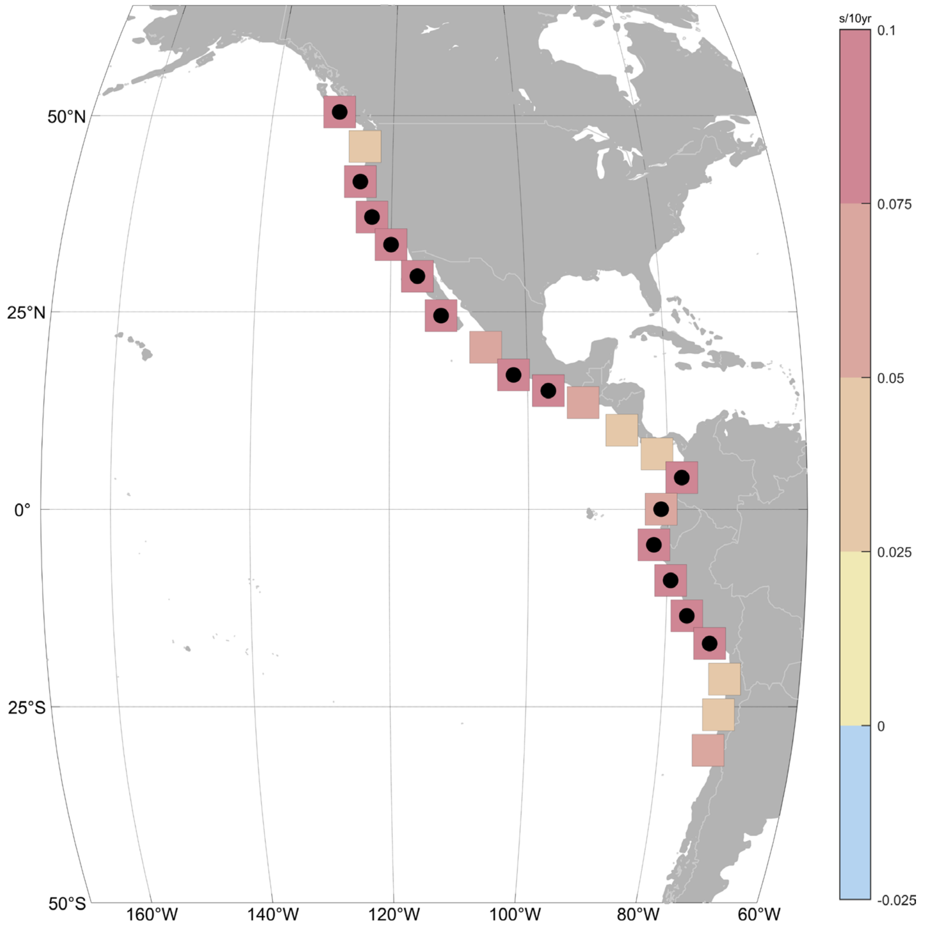

Supplementary Figure 6 | Annual trend in Southern Ocean swells peak period. Trends are expressed in seconds per decade. The black dots indicate statistical significance at the 90% confidence level.

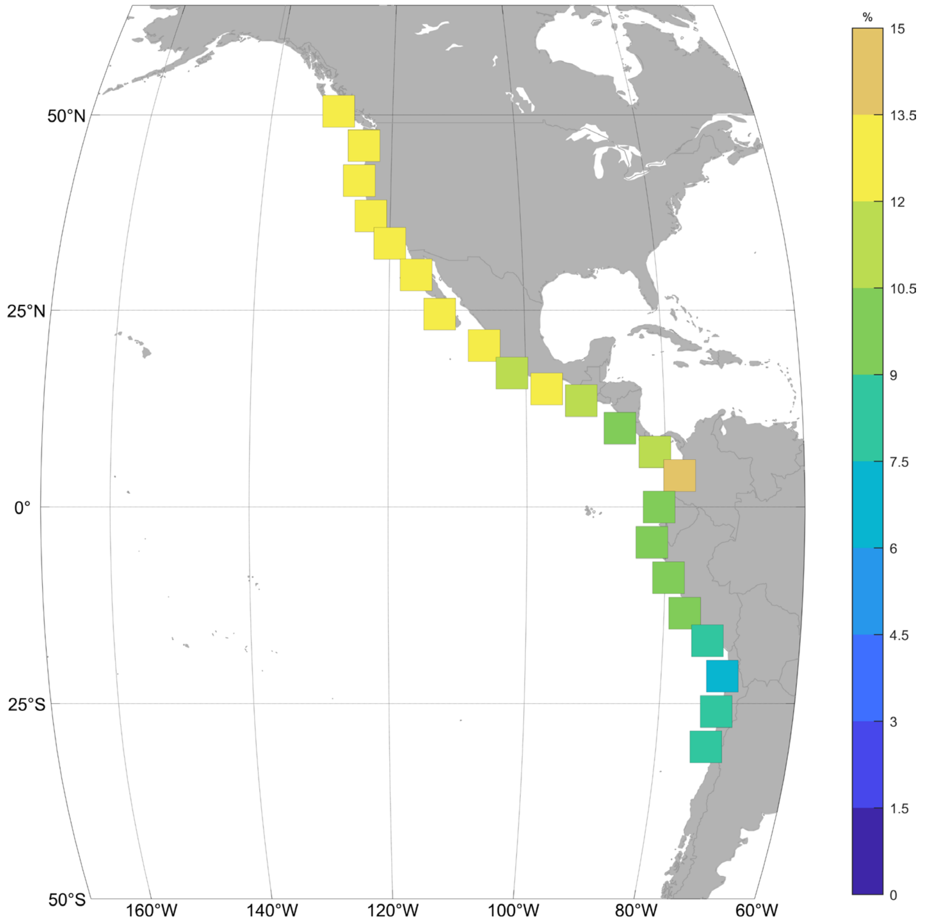

Supplementary Figure 7 | Standard deviation of year-to-year differences in the mean annual power of Southern Ocean swell events.

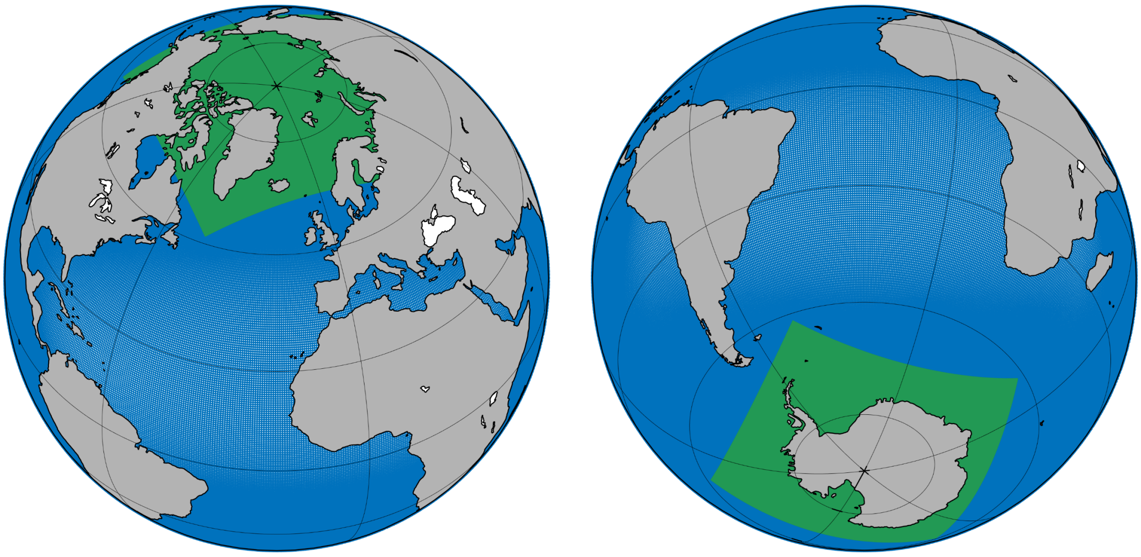

Supplementary Figure 8 | Computational grids of the wave hindcast. Global grid (in blue) and polar grids (in green).

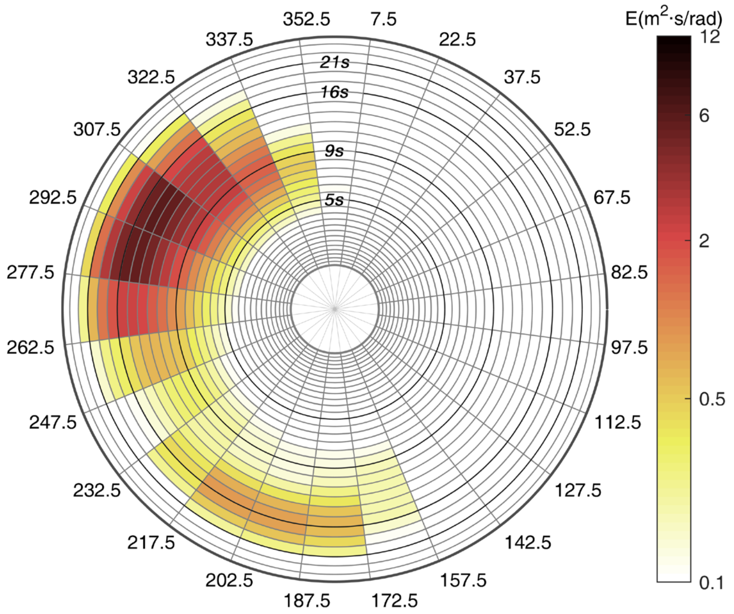

Supplementary Figure 9 | Example wave energy spectrum. Wave energy spectrum at the California coast. Mean energy during the period 1986–2005.

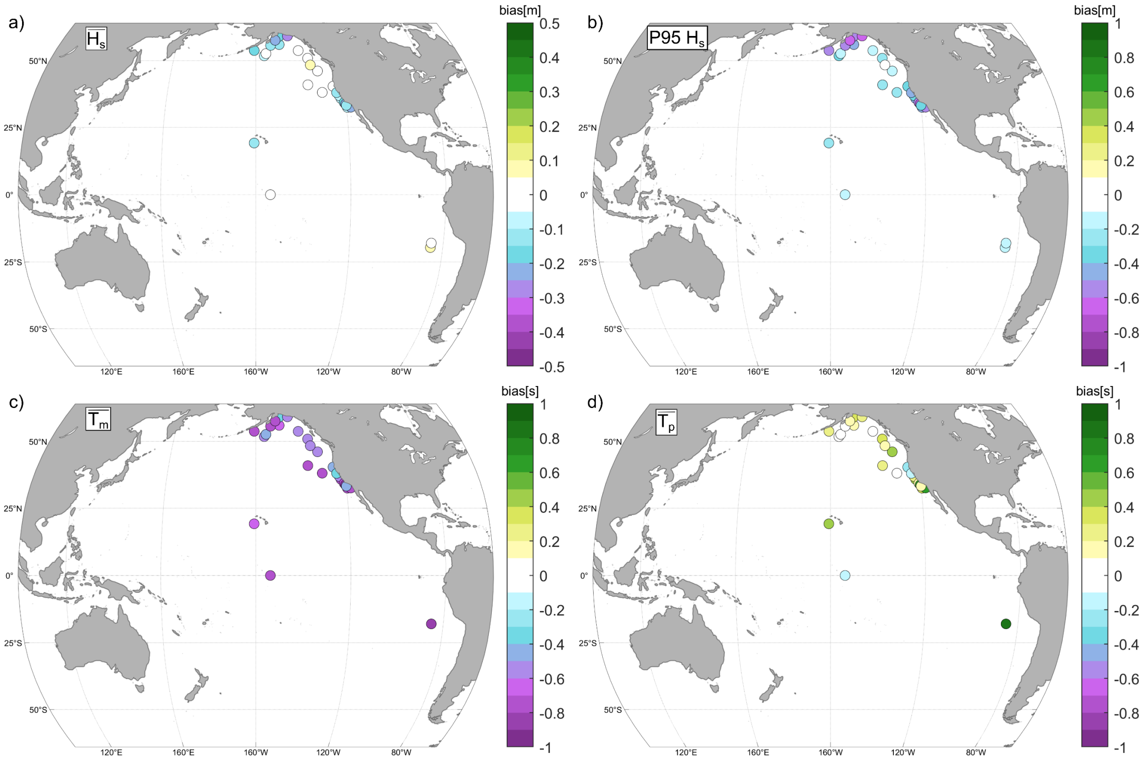

Supplementary Figure 10 | Bias between hindcast and buoy data. a) Mean H_s, b) 95th percentile H_s, c) mean T_{m02} and d) mean T_p.

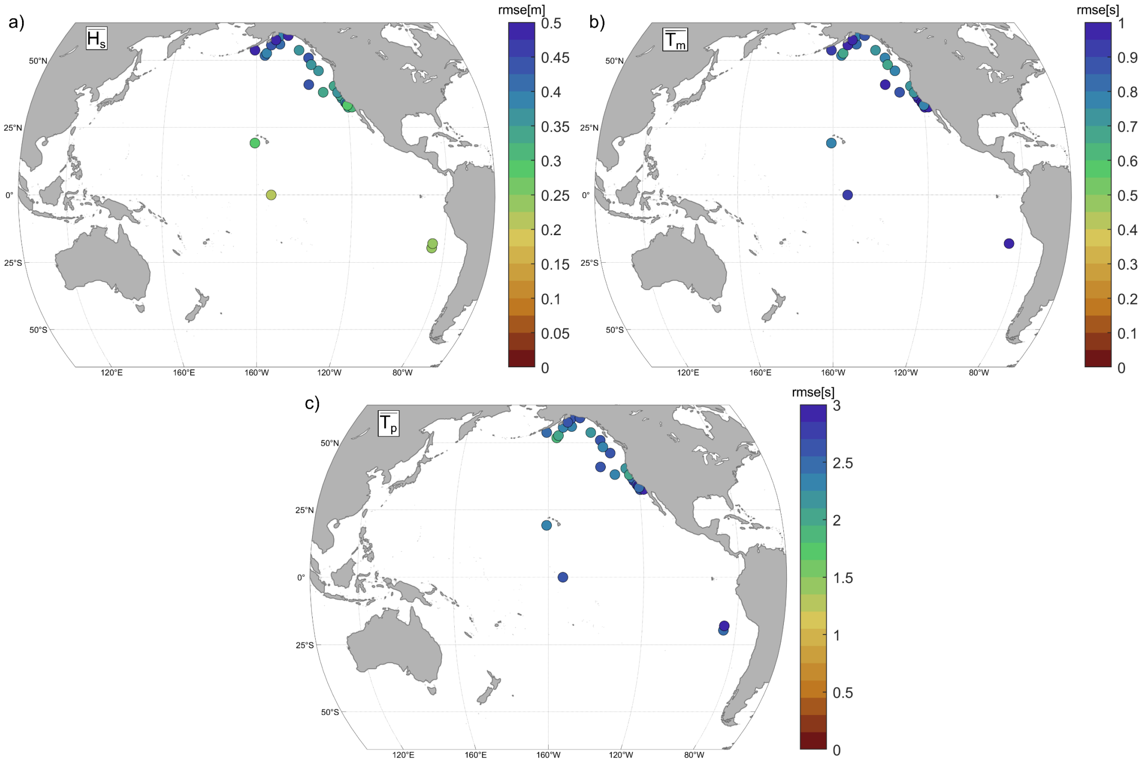

Supplementary Figure 11 | Root mean square error between hindcast and buoy data. a) Mean H_s, b) 95th percentile H_s, c) mean T_{m02} and d) mean T_p.

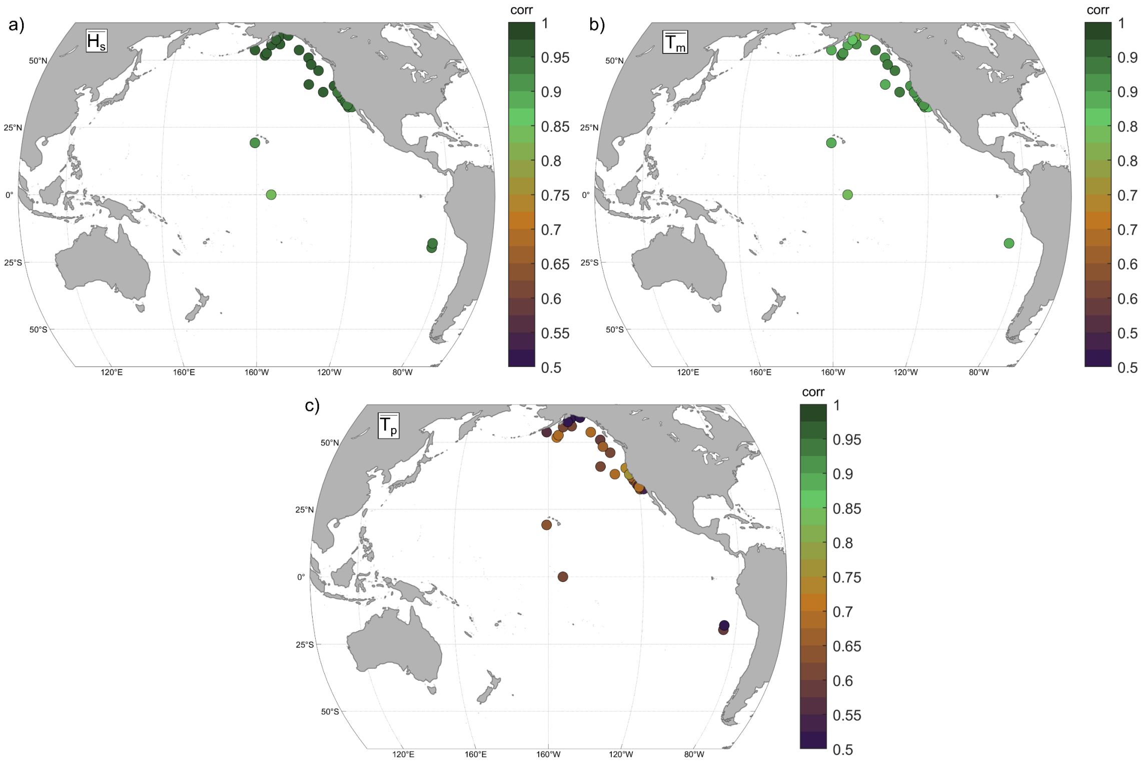

Supplementary Figure 12 | Pearson correlation coefficient between hindcast and buoy data. a) Mean H_s, b) 95th percentile H_s, c) mean T_{m02} and d) mean T_p.

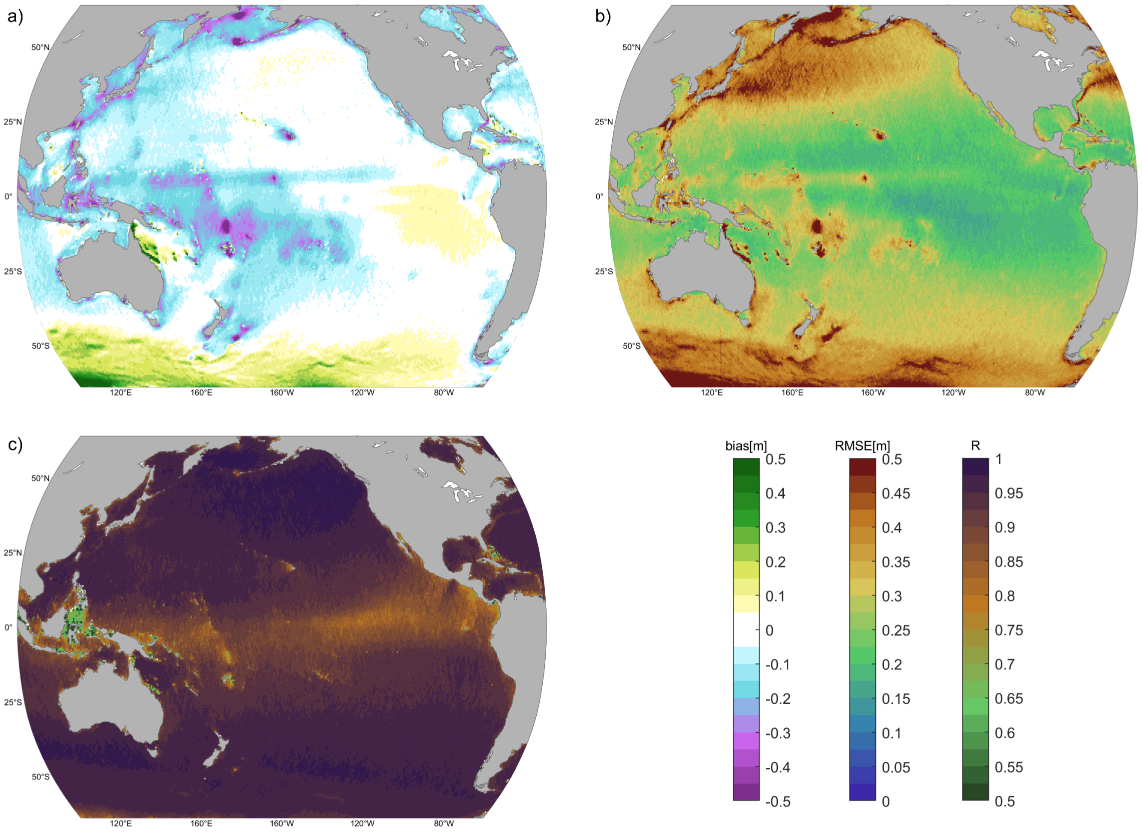

Supplementary Figure 13 | Altimetry-based validation of hindcast data. a) bias in H_s, b) root mean squared error in H_s and c) Pearson correlation coefficient in H_s.

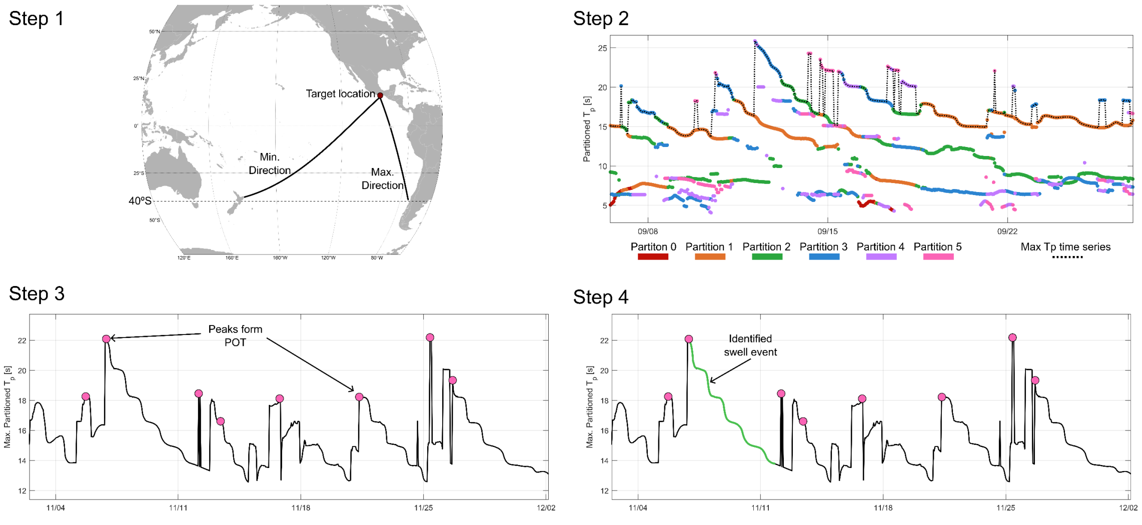

Supplementary Figure 14 | Methodology for swell identification. Each panel illustrates one step of the procedure, from Step 1 to Step 4.

References

1. Coles, S. *An Introduction to Statistical Modeling of Extreme Values*. (Springer London, 2001). doi:10.1007/978-1-4471-3675-0.
2. Tolman, H. Validation of WAVEWATCH-III version 1.15. 33pp. (2002).
3. Chawla, A. & Tolman, H. L. Obstruction grids for spectral wave models. *Ocean Model (Oxf)* **22**, 12–25 (2008).
4. Hersbach, H. *et al.* The ERA5 global reanalysis. *Quarterly Journal of the Royal Meteorological Society* **146**, 1999–2049 (2020).
5. Rogers, W. E. & Linzell, R. S. *The IRI Grid System for Use with WAVEWATCH III.* (2018).
6. Ardhuin, F. *et al.* Semiempirical dissipation source functions for ocean waves. Part I: Definition, calibration, and validation. *J Phys Oceanogr* **40**, 1917–1941 (2010).
7. Hasselmann, S., Hasselmann, K., Allender, J. H. & Barnett, T. P. Computations and parameterizations of the nonlinear energy transfer in a gravity-wave spectrum. Part II: parameterizations of the nonlinear energy transfer for application in wave models. *J. PHYS. OCEANOGR.* **15**, 1378–1391 (1985).
8. Ardhuin, F., O'Reilly, W. C., Herbers, T. H. C. & Jessen, P. F. Swell transformation across the continental shelf. Part I: Attenuation and directional broadening. *J Phys Oceanogr* **33**, 1921–1939 (2003).
9. Battjes, J. A. & Janssen, J. P. F. M. ENERGY LOSS AND SET-UP DUE TO BREAKING OF RANDOM WAVES. in *Proceedings of the Coastal Engineering Conference* vol. 1 569–587 (1979).
10. Stopa, J. E., Ardhuin, F., Babanin, A. & Zieger, S. Comparison and validation of physical wave parameterizations in spectral wave models. *Ocean Model (Oxf)* **103**, 2–17 (2016).
11. Alday, M., Accensi, M., Ardhuin, F. & Dodet, G. A global wave parameter database for geophysical applications. Part 3: Improved forcing and spectral resolution. *Ocean Model (Oxf)* **166**, (2021).
12. Stopa, J. E. Wind forcing calibration and wave hindcast comparison using multiple reanalysis and merged satellite wind datasets. *Ocean Model (Oxf)* **127**, 55–69 (2018).
13. Passaro, M., Fenoglio-Marc, L. & Cipollini, P. Validation of significant wave height from improved satellite altimetry in the German bight. *IEEE Transactions on Geoscience and Remote Sensing* **53**, 2146–2156 (2015).
14. Ardhuin, F. *et al.* Observing sea states. *Frontiers in Marine Science* vol. 6 Preprint at https://doi.org/10.3389/fmars.2019.00124 (2019).
15. Dodet, G. *et al.* The Sea State CCI dataset v1: Towards a sea state climate data record based on satellite observations. *Earth Syst Sci Data* **12**, 1929–1951 (2020).
