# Supplementary material for: Four decades of intensifying Southern Ocean swells along the Pacific coast of the Americas
Source: Nat Commun. 2026 Apr 29;17:5860. doi: 10.1038/s41467-026-71813-1 (PMC13333821; doi:10.1038/s41467-026-71813-1)
Supplement: Supplementary file 2 — Transparent Peer Review file [file 41467_2026_71813_MOESM2_ESM.pdf]

nature portfolio

# Peer Review File

## Four decades of intensifying Southern Ocean swells along the Pacific coast of the Americas

Corresponding Author: Dr Hector Lobeto

**This file contains all reviewer reports in order by version, followed by all author rebuttals in order by version.**

Version 0:

Reviewer comments:

Reviewer #1

(Remarks to the Author)
This paper by Lobeto et al. investigates high energy swells generated in the Southern Pacific Ocean and reaching the eastern coast of the Americas, based on a 45-year numerical hindcast. The paper is well-written and organized, the figures are clear and of very good quality. However, while the method used to partition wave spectra and isolate southwester swells reaching the coast is innovative, the validation of the wave hindcast is not sufficiently detailed and raises important questions. As a consequence, I do not think that the paper can be accepted in its present form and I encourage the authors to address the following important points and to resubmit a new version:
-First, it is well-known that ERA5 winds have strong negative biases at high winds (over 20% for 25 m/s), which can lead to strong negative biases when modelling storm waves if no correction is considered. For instance, Alday et al. (2021) applied a 5% correction above 20 m/s combined to a tuning to 1.75 of the wave growth parameter in the wind input source term (so called “Betamax”). More recently, Gaffet et al. (2025) applied a quantile-quantile correction derived from a comparison between ERA5 winds and wind estimates from satellite altimeters. In the present study, the authors do not mention any bias correction while using ERA5 winds in their hindcast, which questions the reliability of their hindcast, namely for extreme sea states, on which this study is focused.
-Related to the previous issue, the validation of the wave hindcast is limited to a few buoys, all located in the same area (~central California). I am sure that plenty of wave buoys are available off the coasts of Central and Southern America . The authors should demonstrate that their numerical hindcast is accurate in regions where energetic waves reach the coast.
-In section “Historical trends”, L229-232, the authors extrapolate the trends from their hindcast to suggest that wave power will keep on increasing in the future: why that? This is a very strong statement, that should absolutely be supported by wave climate change scenarios. Indeed, several studies did not show any increase in this area for 2100 (e.g. Hermer et al., 2013) while it is not clear that the increase found at low latitude in the Pacific by Lobeto et al. (2022, figure 3) will result in increased wave power along the coast of the Americas. Also, the reasons for such possible increased should be discussed.
I have also identified the following minor issues:
-L17: this sentence is ambiguous, please rather state “SAM+ phases are associated with more energetic swells.
-L35-40: Please note that waves with larger Tp experience more shoaling and hence result in larger wave height at breaking compared to shorter-period waves with a similar amount of energy in deep water. Also, it would be worth mentioning that distant swells usually have narrower spectra and hence drive large infragravity waves, which can have a key contribution to marine flooding.
-L72: it depends on where you close the Indian Ocean but I doubt that swells generated in the Indian Ocean can reach the eastern coast of America.
-L84-86: but this is the only area where you present a validation of your wave numerical hindcast.
-L117: it is obvious that a wave model with a 0.25 km resolution cannot represent wave breaking.
-L132: I think that this is always the case.
-L145: “climate-related anomalies” is not clear to me.
-L272-274: it would be worth reminding why distant long period waves represent a higher hazard compared to normal swells.
-L335-344: several partitioning algorithms are available, could you please justify why you used one based on Tp?
-L403: the wave energy corresponds to the wave height squared, therefore “proportionnal” is inadequate.
-L501: please check “m0_alfa” in eq.2 and “m0_teta” L501.
-L521: here I do not understand, if you run your own model, you can output wave spectra and compute all the period you want, no?

Cited references:
Alday, M., Accensi, M., Ardhuin, F., and Dodet, G., 2021. A global wave parameter database for geophysical applications. Part 3: Improved forcing and spectral resolution, Ocean Model., 166, 101848, https://doi.org/10.1016/j.ocemod.2021.101848, 2021.

Gaffet, A., Bertin, X., Sous, D., Michaud, H., Roland, A. and Cordier, E., 2025. A new global high-resolution wave model for the tropical ocean using WAVEWATCH III version 7.14. Geoscientific Model Development 18 (6), 1929-1946, https://doi.org/gmd-18-1929-2025.

Hemer, M., Katzfey, J. and Trenham, C.E., 2013. Global dynamical projections of surface ocean wave climate for a future high greenhouse gas emission scenario, Ocean Modelling (70), 221-245, https://doi.org/10.1016/j.ocemod.2012.09.008.

Lobeto, H., Menendez, M., Losada, I. J. & Hemer, M. The effect of climate change on wind-wave directional spectra. Glob Planet Change 213, (2022).

Reviewer #2

(Remarks to the Author)
GENERAL SUMMARY

The manuscript “Southern Ocean swells pose a significant and growing threat to the Pacific coast of the Americas” assesses the characteristics of swells generated in the Southern Pacific Ocean (SO) once they’ve propagated to the Pacific coast of the Americas. Swell events are characterized based on different indicators (i.e., wave height, period, direction, and frequency of occurrence), and they are evaluated by their temporal variability across multiple time scales, with an emphasis on long-term trends. The main findings indicate that waves are more energetic close to their generation area, in the southern Pacific Ocean, gradually decreasing their wave height and period from south to north along the Americas. Results also indicate that SO swells varied across seasons, years, and decades, and interannual variability, driven by the Southern Annular Mode (SAM) is the dominant time scale influencing wave power variations across the Pacific coast of the Americas. The analysis, which uses a novel framework to isolate swell events based on spectral data, offers an alternative framework to analyze large SO swells individually and in detail to more traditional approaches that use the basis of total integrated parameters. Overall, the authors do an excellent job of demonstrating the influence of SO swells over the full-length of the Pacific coast of the Americas, and their main conclusions are supported by the high-level framework adopted by the study. Still, there are some areas for further improvement, specifically to clarify and highlight the implications of their findings in a broader context, refine the descriptions of swells related to impacts, and refine/better explain some of the methodological approaches.

Specific Comments

The manuscript addresses a novel and relevant topic, which is highly significant because SO-generated swells with high energy have the potential to cause hazards. I liked how the authors tried to define the impact of the swell events by cross-referencing them with reported hazards along the Pacific coast of the Americas, rather than broadly claiming that because they’re “extreme” they are impactful. However, I believe the title overstates the relationship the authors describe. First, the authors compile a list of events that cause damage in Table 1. It is unclear whether these events are related to SO swell in the articles, or whether the authors found damaging events and then later related them to events they characterized as swell. If the latter is the case, they probably shouldn’t be described as “numerous SO swell events” causing some form of damages (line 74-75). If the reports directly relate the damage/impacts to SO swell, then that should be more clearly stated.

Following on, it is noted that the events don’t necessarily reflect all the countries affected or damage caused, but the statement is made that “The collected information shows that the impact of these events is more frequent on the coasts of South and Central America.” I don’t believe the assessment done is rigorous enough to make this statement – and if this table is part of the results, more detail must be provided on how events were selected, the methods for finding the events, etc. in order to ensure the selection criteria does not bias the results. If the point of this table is to show that some of the SO swell events may in fact cause impacts, this could be brought up later once swells are characterized.
For example, Cross referencing – 23 swells compared to 900 at each location – isn’t a very large sample size and it doesn’t necessarily indicate that all the events being defined as powerful do cause impacts. The manuscript would benefit from more explicitly defining how the cross-reference method was implemented in the methods section.

On that note, some of the jargon surrounding the hazardous nature of these events needs to be more clearly articulated. For example, on Line 144 – the swells are described as “potentially damaging events” and this terminology is used throughout the manuscript, but in other instances, hazardous event terminology with more certainty is used, like in line 302 with “hazardous SO swells”. At the beginning of the “Discussion & Conclusions” section (line 257), swells in the SO are mentioned to be important to coastal hazards, but this is not always the case. These swells can lead to hazards but given the right circumstances! This is contradictory to what is mentioned in the introduction regarding the US Pacific coastline, where these swells have “less ability” to cause impacts and may be linked to recreational activities, like surfing.

Another topic I’d like discussed a bit more is whether wave transformation processes and bathymetry may impact coastal results. It states in the manuscript that coastal points were sufficiently far away from wave breaking, but what about wave

shoaling? The majority of coastal points are in deep water, but a few locations are located in 100 – 600 m of water, and with longer period waves (e.g., >14s) you could calculate that the wave base could in fact interact with the seafloor, beginning wave shoaling processes. One of the coastal points in the pacific northwest of the US has very different results than its surrounding geography – and I wonder if that may be part of the reason.

Along those same lines, the depth of the buoys can be quite different when compared to the equivalent hindcast point. For example, P4 is at 1906 m and an equivalent in location buoy at 123 degrees W is at 600 m – how different can the partitioned, maximum Hs be between these two?

I'm also wondering how many of the same swell events impact large stretches of coastline. How many of the top 20 events each year also occur in neighboring stations? This would be a cool analysis to complete if not already done.

The authors define "frequency of occurrence" based on the 20th-ranked annual event as the threshold to count annual exceedances. How sensitive are results in the trend analysis to the chosen threshold?

Can you justify the approach for ENSO calculations? Are they occurring over the calendar year or over some wave year focused on not breaking up the "wave season"? If you have a calendar year that switches from El Nino to La Nina like 1983 or 2016 – would the annual average accurately characterizes the index and the extremeness of the ENSO phase?

Lines 556 to 557, describe the interannual variability in swell indicators. Does the interannual variability consider JUST 2 points (i.e., end-point method) from all the events for the 45 years? If that is the case, this maximum-minimum difference is potentially more representative of the "bounds of variability" within the dataset or what the maximum interannual variability could be in each location based on 45 years of available data. This difference could potentially capture a maximum and minimum related to climate modes with decadal or multi-decadal periodicities, representing a difference that is not frequently experienced over the period of study, and thus, might not represent the typical/more frequent interannual variability of the region. If an end-point method is being considered, the potential large difference could not be frequently observed. Have the authors assessed how often a 60% variability is observed (reported in lines 296-298 at specific locations)? An end-point approach could potentially overestimate the dominance of interannual variability in wave power variability by capturing variations related to infrequent climate modes. To better represent typical interannual variability, one suggestion is to considering differences across all data points (consecutive or not) and then averaging them to provide a more representative variability of all potential climate modes influencing oscillations across the Pacific coast of the Americas.

The authors acknowledge the importance of both the Southern Annular Mode (SAM) and the El Niño Southern Oscillation (ENSO) because of their correlation to wave power. This analysis could be potentially replicated but considering the interaction of these two climate modes during the same period. Positive SAM and negative ENSO are known to increase wave heights in the SE Pacific, as acknowledged by the authors. Have the authors considered doing so? This could be done with an interactive term and could potentially increase the correlation or reveal new findings regarding different combinations of phases.

The discussion and conclusions section could be strengthened if the authors highlight the implications of overlooking potentially hazardous secondary events, such as SO swells, in the context of changing frequency, especially because some locations do show significant increases. This is particularly evident from lines 287 to 290. This could also be strengthened if events of different frequencies were considered in the definition of this indicator; as previously mentioned, rarer or more frequent events could yield different results in terms of changes in the frequency of occurrence than the 20th-ranked event/98th percentile. In this section, findings related to changes in swell indicators associated with SAM in the NH could help emphasize the importance of large-scale climate patterns for projecting potential hazards, especially in the NH where the SO swells do not dominate the wave climate.
Regarding swell indicators, has the historical variability in other metrics, like variance and standard deviation, other than the mean been explored? The mean could be not experiencing significant trends, but other metrics could, and this would strengthen the characterization of the historical variability of SO swells.

Trend calculation, page 12 – have the authors considered using a more robust method for assessing linear trends? Like Sen's Slope estimator, which is known to be more robust to outliers and non-normality in the data, which should be expected with the datasets used.

The discussion and conclusions would benefit from incorporating a more explicit discussion of the implications of this study in a broader context. While the novelty of the work is clear, the manuscript frames the significance of results on a more regional basis does this have implications for other areas across the globe?

Line-by-line Comments
- First paragraph is really well written and very easy to understand.
- Line 39: what does "moderated at long distances" mean?
- Lines 62 – 64: There are a bunch of publications that look at the combination of different sea level components, especially on the US West Coast. I was surprised they weren't cited here. You don't have to cite them, but could strengthen this part. They are listed below.

Serafin, K. A., Ruggiero, P., & Stockdon, H. F. (2017). The relative contribution of waves, tides, and nontidal residuals to extreme total water levels on US West Coast sandy beaches. Geophysical Research Letters, 44(4), 1839-1847.

Melet, A., Meyssignac, B., Almar, R., & Le Cozannet, G. (2018). Under-estimated wave contribution to coastal sea-level rise. Nature Climate Change, 8(3), 234-239.

Melet, A., Almar, R., Hemer, M., Le Cozannet, G., Meyssignac, B., & Ruggiero, P. (2020). Contribution of wave setup to projected coastal sea level changes. Journal of Geophysical Research: Oceans, 125(8), e2020JC016078.

Marcos, M., Rohmer, J., Vousdoukas, M. I., Mentaschi, L., Le Cozannet, G., & Amores, A. (2019). Increased extreme coastal water levels due to the combined action of storm surges and wind waves. Geophysical Research Letters, 46(8), 4356-4364.

• Line 73: "This area" will be the study for the research – it's not clear what this area pertains to. The SO? The pacific or eastern Indian ocean? Please clarify.
• Line 105: if the wave dataset is a hindcast, it should be called that here, rather than just in the methods, so there is no mistaking it for observations.
• ~Line 180 Seasonal variability – it might be interesting to bring in the publication below, which found off the California coast the southern hemisphere swell events were happening during daily weather types occurring in austral winter
Rueda, A., Hegermiller, C. A., Antolinez, J. A., Camus, P., Vitousek, S., Ruggiero, P., ... & Mendez, F. J. (2017). Multiscale climate emulator of multimodal wave spectra: MUSCLE-spectra. Journal of Geophysical Research: Oceans, 122(2), 1400-1415.

• Lines 211-213 – it doesn't seem that correlation values were below 0.4 at all locations. From Figure SM6, it actually seems like values are higher than 0.4 in points 9, 11, 13, and 15 – 18.
• Lines 274 and 279 – why only flooding is accounted for? What about the potential for coastal erosion? Wave setup is generally more related to flooding, but runup has been associated strongly with erosion.
• Line 303: increasing exposure to what?
• Paragraph from lines 318 – 324: are there any studies in the literature that show the compounding effects of SLR + increases in swells + SAM? The interaction of more than the two dynamics mentioned, but there are potentially more processes involved in this interaction, so it would be beneficial to the manuscript to acknowledge this.
• Lines 417 to 419: results for trends are based on individual partitions, right? Not on total integrated wave parameters? It's not clear from the writing
• The framework steps listed from 455 to 485 are somewhat difficult to follow, especially from steps 1 to 4. If some of these steps can be shown with examples in Figure SM2, that would be helpful.
• Page 10, Step 6 – why 48h? Is this related to the duration of ET/storms?
• Page 10, Steps 3-9 - Why 100 events? A bit confused where this number comes from.
• Line 552 – the term "climate variability" is brought up here for the first time, what does this mean? Does it include all the variabilities listed below?
• Lines 554-555: has seasonal variability been assessed for each season separately OR the maximum relates to winter and the minimum to summer? This is unclear.
• Figure SM2: where are the partitions T0, T1,…T6 in this figure? Is each dot a partition? Or each partitions have its own time series in this plot with a sequence of dots? It might be helpful to highlight how the partitions are represented in these plots so that the readers can better understand the explanation of the algorithm steps and visualize the independence of swell events. Maybe highlight which is the corresponding Tp and direction to the peak Hs used to derive all stats from the analyses.
• Figure SM4: it is very challenging to visualize the gray arrows, they should be thicker and perhaps represented by a different color. Another suggestion is to consider using dashed gray lines, this would be interesting because they would overlap with black arrows where the direction does not change as much and would highlight even more the different toward the north.

Reviewer #3

(Remarks to the Author)
Major comments:

The analyses and findings presented in this study are good and well worth reporting to the scientific community. The authors present a robust methodology for studying Southern Ocean generated swells that reach the west coast of the American continent, with a focus on the 20 most intense events per year (one every ~2.5 weeks on average). While not entirely novel, there are interesting (and potentially important) results regarding seasonal variability and influence of climate oscillations (ENSO and SAM), alongside an assessment of long-term trends. The separation of the swell wave extremes' variance into seasonal, interannual and long-term trends, utilising swell identification and tracking, is an excellent contribution.

However, in my opinion, the manuscript suffers from several significant flaws which need to be ameliorated before publication. Considering its focus on coastal damage and concomitant risk associated with swell events, the assertion that "Specifically, the Pacific coast of the Americas is one of the coastal regions in the world most affected by these events" (lines 70-71) needs to be better unpacked and put into context. For instance, over the last decade, an increasing number of journal articles have identified and discussed the impacts of distant-source swell events (with Southern Ocean sources in many cases), modulated by tides and sea level, to Pacific and Indian Ocean Island nations. Only one of the oldest of these papers (Hoeke et al. 2013) is cited but not really discussed. A very non-comprehensive list of more recent publications includes:

Wadey, et al. (2017). Coastal flooding in the Maldives: an assessment of historic events and their implications. Nat. Hazards

89, 131–159. doi: 10.1007/s11069-017-2957-5

Ford, et al. (2018). Inundation of a low-lying Urban atoll island: Majuro, Marshall Islands. Nat. Hazards 91, 1273–1297. doi: 10.1007/s11069-018-3183-5.

Canavesio, R. (2019). Distant swells and their impacts on atolls and tropical coastlines. The example of submersions produced by lagoon water filling and flushing currents in French Polynesia during 1996 and 2011 mega swells. Global and Planetary Change, 177, 116-126.

Wandres, et al. (2020). Distant-source swells cause coastal inundation on Fiji's coral coast. Front. Mar. Sci. 7:546. doi: 10.3389/fmars.2020.00546.

Andréfouët, et al. (2023). Hydrodynamical impact of the July 2022 'Code Red'distant mega-swell on Apataki Atoll, Tuamotu Archipelago. Global and Planetary Change, 228, 104194.

Wandres, et al. (2023). Wave climate variability and trends in Tuvalu based on a 44-year high-resolution wave hindcast. Journal of Geophysical Research: Oceans, 128(4), e2022JC019523.

As outlined in Storlazzi, et al. (2018) and other papers, such events represent an increasingly existential threat (when coupled with sea level rise) to many such oceanic communities. This manuscript should thereforcompare and contrast the Pacific coast of the Americas with other areas of the world at risk of such swell-driven coastal impacts as reported in these and other studies, including (where applicable) other studies' findings on Southern Ocean swell extreme event trends and variability. For instance, a single event may result in damages/loss equivalent to a substantial portion of annual GDP at many oceanic island locations, which undoubtably suffer the highest relative impacts and future risk; the "Pacific coast of the Americas", on the other hand, likely suffer significantly higher overall gross level of impacts in terms of total economic costs (owing to their much larger size and populations). This is just an example - to reiterate my overall view - particularly given the general audience of Nature Communications - a better framing of the context of the findings of this study against other climate-related hazards studies, especially ones with similar motivations/underlying coastal hazard processes, is required.

Storlazzi, Curt D., et al. "Most atolls will be uninhabitable by the mid-21st century because of sea-level rise exacerbating wave-driven flooding." Science advances 4.4 (2018): eaap9741.

Other areas that need to be improved:

• The reported research is (apparently) based on a (new) global wave hindcast developed specifically for this analysis? Building a global implementation of WAVEWATCH III, with tuning and verification, is not a trivial exercise … why weren't existing published, well verified wave hindcasts such as the CAWCR (Durrant et al. 2014/ Smith et al. 2021) or GOW2 (Perez et al. 2017) used? As far as I know, these existing wave hindcasts (and undoubtably others) make available the same spectral partitioning variables used in this study. They also offer extensive global verification reports from satellite and from buoys, enabling understanding of strengths and weaknesses of these extant hindcasts to different wave conditions. Verification of the wave hindcast presented here is (apparently) limited to significant wave height (and not other bulk or partitioned wave parameters) at five coastal wave buoys offshore of central and southern California (Figure SM3/Table SM2), thus representing only a small portion of the study region, near its northernmost limit, raising questions about the hindcasts' overall veracity. Both better justification of why extant, published wave hindcasts were not used and much better verification/assurances of the wave hindcast that was used is needed.

• Regarding the wave partition tracking algorithm ("Isolation of swells") upon which this study relies, there appears to be the assertion that the "algorithm has been previously applied using total integrated wave parameters". However, there is a nearly 30-year history development of space-time tracking of wave spectral partition algorithms: see Delpey, et al. (2010) and citations therein for a good review of its history and use up to that point, including a more complex adaptation of the swell travel distance equation than the Eq. 1 used in this paper. While the partition tracking algorithms used here may be different and improved from previous, it is not clear what makes them "novel" as stated in the abstract and discussion. More discussion and justification in this regard is needed.

Delpey, M. T., Ardhuin, F., Collard, F., & Chapron, B. (2010). Space-time structure of long ocean swell fields. Journal of Geophysical Research: Oceans, 115(C12).

Technical aspects that could be improved/better discussed:

• The correlations between swell mean wave power and the SAM and ENSO indices are presented, but the authors do not indicate whether these correlations are statistically significant at the 95% confidence level. That might be because they compare annual values, what would happen if a higher temporal resolution were used?

• The authors do show statistical significance in the trends, but only at the 90% confidence level. What would happen if the 95% confidence level is chosen instead? Are the trends statistically significant then?

• Trend magnitude vs. model uncertainty: the reported trend in wave height (~0.3 cm/year, yielding ~13.5 cm over 45 years) is very close to the model's root-mean-squared error (~11 cm, from Figure SM3). Is there scope to discuss this?

• The manuscript does not have an in-depth discussion on the reason for the observed trends. Can they be linked to or compared against, for example, the results presented in Young and Ribal (2019), or the trends in the Southern Annular Mode during the historical hindcast?
Young, I. R., & Ribal, A. (2019). Multiplatform evaluation of global trends in wind speed and wave height. Science, 364(6440), 548-552.

Minor notes:

- Line 14: "characterizing their primary characteristics" might need a rephrasing.

- Line 25: "For that matter…" consider a different turn of phrase. It doesn't really make sense.

- "destructive" … "damage" … this is an oversimplification. Wave power (the ability to do work) is roughly linearly related to breaking wave height, wave setup and wave overtopping, etc… and likely more correlated with erosion, although that is more complex and depends on many other factors.

- Introduction, first paragraph really could/should be better written, also recommend reorganising the Introduction – e.g. lead with the discussion of May 2015 and link it with other event-driven damaging/inundation event recorded in earlier research, as suggested above.

- Lines 34-35: "Swells [..] follow great circles paths, with minor deviations due to ocean currents". Ocean currents can have a massive impact on ocean swells, please see Gallet and Young (2014).

Gallet, Basile, and William R. Young. 2014. "Refraction of swell by surface currents." Journal of Marine Research 72, (2).

- More rigorous definition of Southern Ocean is needed, i.e. "we define it as south of the *** parallel"

- Lines 40-42: "Additionally, the height of the infragravity wave induced by long mature swells, which has been found to be correlated with the product between the wave height and the squared period of the generating waves7,8" … this statement, and the articles referenced, refer to the bound wave release mechanism of coastal infragravity wave generation. What about the breakpoint mechanism (which has a different, less consistent relationship with wave period)? Consider whether this needs to be re-phrased to include this mechanism or omitted.

- Figure 1: How were the mean wave energy gain and losses calculated? Provide an explanation for the observed patterns of gains and losses of wave energy.
- Lines 136-138: why is this approach taken (i.e. to characterize the peak of the event as "corresponding to the peak spectral energy density of the event, i.e., when $Hs$ is at its maximum."), when in the introduction section, the importance of wave power (energy flux) is discussed and is a stated integrated wave parameter used?

- Figures 3 and SM6: It might be worth marking the points that show a statistically significant correlation.

- Figure 3: Why not putting the left panel of Figure 3 to the right of Figure 2, and combine the right panel of Figure 3 (correlations with the Antarctic Oscillation Index) with Figure SM6 (correlations with the Multivariate ENSO Index)?

- Line 126: Is there any sensitivity to selecting more events per year?

- Figure SM1: The southernmost point the authors defined is at 30.5°S latitude. Can this be extended further South? With the current configuration the authors are missing the area from Chiloé Island to La Serena, which have a considerable population density (https://sedac.ciesin.columbia.edu/downloads/maps/grump-v1/grump-v1-population-density/chldens.jpg). Viña del Mar, a very popular tourist destination which has been affected by extreme wave events in the past (e.g.,: https://www.infobae.com/america/america-latina/2024/12/29/un-fuerte-oleaje-sorprendio-a-los-turistas-en-vina-del-mar-y-las-autoridades-chilenas-activaron-medidas-de-seguridad/) is at 33°S and therefore not included in this analysis.

- Figure SM3: Please mention what the grey and red points mean in the caption. These results show a clear positive bias (overestimation) by the model, which could be discussed.

- Lines 206-208: The pioneering study of Hemer et al. (2010) should also be included as a reference here.

Hemer, M. A., Church, J. A., & Hunter, J. R. (2010). Variability and trends in the directional wave climate of the Southern Hemisphere. International Journal of Climatology: A Journal of the Royal Meteorological Society, 30(4), 475-491.

- Figure SM4: The caption needs fixing; the grey and black arrows represent different things.

- Line 222: Why not using the 95% confidence interval as it's usually done? How would the results change?

- Lines 226-228: "Trends in wave period" -> Where are they? Can they be added to Figure 4 even if only statistically significant at 55% of the locations?

- Lines 323-324: “As a result, coastal water levels in areas affected by these swells are likely to show an accelerating positive trend due to the interaction of these two dynamics.”… I don’t follow this logic.

- Lines 405 and 407: I suggest changing “corresponding to” to “associated with”.

- Line 420: in the employed numerical modelling -> change to “in the employed numerical model”

- Line 429: remove the word “secondary”

- In the Supplementary Material, in the “Swell identification algorithm” section, consider incorporating a formula for the weighted Euclidean distance, it might help improve the flow of the reading (see for example Jiang, 2019)

Jiang, H. (2019). Spatially tracking wave events in partitioned numerical wave model outputs. Journal of Atmospheric and Oceanic Technology, 36(10), 1933-1944.

Reviewer #4

(Remarks to the Author)
I co-reviewed this manuscript with one of the reviewers who provided the listed reports. This is part of the Nature Communications initiative to facilitate training in peer review and to provide appropriate recognition for Early Career Researchers who co-review manuscripts.

Reviewer #5

(Remarks to the Author)
I co-reviewed this manuscript with one of the reviewers who provided the listed reports. This is part of the Nature Communications initiative to facilitate training in peer review and to provide appropriate recognition for Early Career Researchers who co-review manuscripts.

Version 1:

Reviewer comments:

Reviewer #1

(Remarks to the Author)
In this new version, the authors seriously addressed the comments raised by the two reviewers and their paper improved substantially. I only have a few last minor suggestions (please note that I refer to line numbers in the clean revised version):

-in their rebuttal, the authors state that no wave buoy data in the Southern hemisphere meet with their quality requirement (« a minimum temporal coverage of 10 years and a maximum time gap of 40% »). This is not true, for instance data from the buoy « Stratus » (WMO 32012, off the Chile/Peru border, easily accessible through the Copernicus portail https://marineinsitu.eu/dashboard/) are available from 2007 to 2018 and has less than 10% of gaps. Furthermore, this data includes several events with Hm0 reaching 5 to 6 m, including the event of May 2015 discussed by the authors in their introduction. Next I found on figure SM10 that this buoy as well as WMO 32302 were apparently included in their validation procedure : please check and clarify. Could you show how the event of May 2015 s reproduced in your hindcast ?

-L25 : « interaction » is not fully appropriate in this context, do you mean « coincidence » ?

-L36 : please rephrase « waves …with waves »

-L204 : how do you explain that a highest proportion of SO swells reach the southern coast of Mexico and Guatemala in MAM and not JJA as in other locations ? Could it be a matter of wave direction, e.g. coming more from the south in MAM compared to JJA ?

-caption figure SM3 : the variable displayed is apparently Hs and not wave energy.

Reviewer #2

(Remarks to the Author)
The authors have made substantial progress since the first round of reviews, including:
• Terminology and definitions have been clarified. For instance, authors now better distinguish between “potential for hazards” and “hazardous events” consistently across the manuscript.
• Revisions improved clarity and transparency on assumptions and methodological choices, such as thresholds and event selection criteria.
• The shift from calendar to ENSO-year definitions appropriately captures full ENSO phase transitions within the same analysis period.
• Authors now use a more robust approach to assess interannual variability, replacing the previous end-point method, which

was pointed out as potentially problematic. This corrected the earlier overemphasis on interannual variability, and seasonal variability emerged as the dominant mode of variability instead of interannual variability.

Remaining Concerns and Suggestions

• If Table 1 was used to help develop the algorithm and validate impactful events, then it would be useful if it included the wave characteristics (Hs, Tp, and Dp) associated with the Southern Ocean (SO) swells events. Suggesting that authors indicate which events were used in this validation and to potentially add extra columns with the average ± standard deviation of wave height, wave period, and wave direction; this would make the table more than introductory, but also a methodological and interpretive reference point.

o Some of the sources aren't actually sources. For example, patch.com is a general community-specific news site across the United States. There over 30,000 community-level patch news providers (or so the website says). Can the authors put the actual reference link for where this was found? This goes for the other generic references in this table too.

• L135–136: The language used here suggests that all the points are deep enough to avoid shoaling processes, even though the rebuttal (pg 17) states that may not be the case for two nodes, which may or may not show variation outside of the trends seen across the broader region. So, while it's true that the majority of locations (and those two locations) are at a depth that is "sufficient to avoid substantial shoaling processes" an acknowledgement of the two locations that may effected by shoaling processes would be more transparent as the text says the avoid them, and because on average these waves are 15 s, which means a good amount of them have longer periods than that too.

• L185 – 188: The way this line is written, it's unclear whether SO swell wave height or total wave height is larger. It's unclear from SM4 too, whether SO swell wave height is being subtracted from the total wave height, or vice versa.

• L216 and L625: If I'm understanding correctly, the annual values of the MEI index was calculated based on an ENSO year definition of May-April, but the annual wave conditions were computed over the calendar year. Could this potentially be a reason for less strong correlation? If waves were recalculated to reflect the same time period ENSO is being defined, would the correlation change?

• L586: The section here should explicitly state that these methods are performed by individual sites. This is particularly important for the Frequency of Occurrence threshold.

• The discussion in L225–241 may needs some revision. It is inconsistent to describe correlations of 0.6–0.7 (maximum correlations observed) with SAM as "high" but correlations < 0.6 with ENSO as "weak," when the threshold values overlap. The interpretation should be consistent. For example, potentially point out lower correlation values for ENSO and less significance, and highlight the locations that have a strong correlation too.

• Lines 313 – 327: One thing to note here, especially in relation to the NH. The largest waves (height and period) from a hazard perspective in Washington, Oregon, and Northern California occur in December, January, February, when there are very few SO swells occurring (Figure 2 right). These waves seem much larger than those from SO swells, and likely what are used for coastal hazards protection/design. So disregarding them SO swells in analysis potentially won't lead to an underestimation of coastal risk in design in this location. I don't think Figure 4SM would bring up the true difference between waves from DJF compared to the SO swell, because they're not really occurring at the same time. See some citations below with general wave climatology for the region.

García-Medina, G., Özkan-Haller, H. T., & Ruggiero, P. (2014). Wave resource assessment in Oregon and southwest Washington, USA. Renewable Energy, 64, 203-214.

Allan, J. C., & Komar, P. D. (2006). Climate controls on US West Coast erosion processes. Journal of coastal research, 22(3), 511-529.

• SO swell impact to hazards would maybe increase them at times of the year there wouldn't be as likely to have hazards. I wonder, for example, the role they play in sneaker type waves (e.g., Garcia-Medina et al., 2018)

García-Medina, G., Özkan-Haller, H. T., Ruggiero, P., Holman, R. A., & Nicolini, T. (2018). Analysis and catalogue of sneaker waves in the US Pacific Northwest between 2005 and 2017. Natural Hazards, 94(2), 583-603.

• Christie Hegermiller has a few papers that seem to back up some of these points for Southern California – that SH generated swell can contribute a lot to local waves, especially in JJA. (Figure 2 in the GRL paper referenced below has a nice diagram of this). Figure 1 shows how the contribution can vary at a high resolution locally as well – which might be a nice point to bring up based on the coarseness of the results presented within this manuscript.

Hegermiller, C. A., Rueda, A., Erikson, L. H., Barnard, P. L., Antolinez, J. A. A., & Mendez, F. J. (2017). Controls of multimodal wave conditions in a complex coastal setting. Geophysical Research Letters, 44(24), 12-315.

Hegermiller, C. A., Antolinez, J. A., Rueda, A., Camus, P., Perez, J., Erikson, L. H., ... & Mendez, F. J. (2017). A multimodal wave spectrum–based approach for statistical downscaling of local wave climate. Journal of Physical Oceanography, 47(2), 375-386.

• L362–364: Specify the period over which the positive SAM trend is observed to better clarify why it may relate to increased SO swell energy.

• L646–648: The explanation of how seasonal variability is computed could be clarified — especially how seasonal means are derived. Are seasonal means calculated per year and then averaged, yielding four mean values (Winter, Spring, Summer, Fall)?
• Figures in general: Labeling panels (e.g., a, b) would be better than indicating location (e.g., left, right), especially when the number of panels increases (e.g., Figure 4). Having to keep track of top-left,top-right,bottom-left becomes slightly less easy to read.
• Figure 2 – text cut off from right panel figure.
• Data Availability statement information about access to the dataset analyzed in the study is provided, but information about access to the data generated during the study are not provided.

Reviewer #3

(Remarks to the Author)
I think the manuscript is substantially improved, particularly the introduction and overall presentation of the context of this study and implications. Also, I thank the authors for the thorough response to reviewers' comments.

There still could be better links between the why/what of the events of May 2015/May 2007 and the following paragraphs, but that is purely for readability and should be no barrier to publication.
In my view, there are still a few, primarily technical, issues that need to either be addressed or that at least more thoroughly explained for the article to be considered rigorous.

Key among these is that the wave hindcast verification/validation is still insufficient. It would be much preferable if the "dedicated article describing the full validation" described in the response to reviewers was sufficiently complete to cite in this article. I understand sometimes logistics are such that papers are published out of preferred sequence, and that additional validation figures have been added to the supplementary materials, but it still doesn't answer questions about how this wave hindcst(the source data central to the findings of this study) compares to other contemporary efforts. Most important though, is the (continued) reliance on five buoys, clustered in a small region of the very large study area, for verification of spectral partitioning and swell tracking central to this analysis – the assertion in the response to the reviewers that other (e.g. NOAA) buoy did not "meet the required standards" is difficult to follow. What are the "required standards"? Jiang et al. (2016) applied a very similar swell tracking algorithm to multiple NDBC (NOAA) buoys to study swell arrival time, so it can definitely be done. Also, Portilla et al. (2009) –cited in this sudy - use a convolutional filter for noise removal. Perhaps these might help? More concise discussion on what the authors' issues around applying the spectral partition/swell tracking algorithm, and ideally broader validation with additional buoys would greatly improve the rigour of this paper.

In "Trend calculation": not sure that …" consistent with conventions adopted in IPCC" reference is appropriate. To my knowledge IPCC conventions are not that straightforward and are in any case, not that clearly stated in the AR6/Working Group 1 report. But this is a very minor comment and addressing it (or not) should not be any barrier to publication.

Finally, I'm slightly uncomfortable with the assertion in the discussion and conclusion that "This approach has enabled a detailed and individualized study of large SO swells *for the first time*, which would have been difficult to achieve using more traditional approaches on the basis of total integrated parameters." (I have added the italics).
Algorithms for spectral partition analysis and tracking of swells (not just the space-time methods outlined by Delpey et al, 2010), including those with generation zones in the SO, can be widely found in the literature. For example, a quick search turns up:
Zheng, Zhenjun, et al. "Investigation of multimodal wave climate using spectral partitioning and wave system tracking algorithms." Ocean Modelling 188 (2024): 102327.
Algorithms presented in this manucript's study may differ/improve upon in details of previous studies' algorithms, and it is likely the first time swell partition and tracking has been used to identify extremes over such a long coastline at climate timescales (for which I applaud the authors), but (if I am to be pedantic) it seems a very large stretch to say the authors' approach has enabled a detailed and individualized study of large SO swells *for the first time*.

Reviewer #4

(Remarks to the Author)
I co-reviewed this manuscript with one of the reviewers who provided the listed reports. This is part of the Nature Communications initiative to facilitate training in peer review and to provide appropriate recognition for Early Career Researchers who co-review manuscripts.

Reviewer #5

(Remarks to the Author)
I co-reviewed this manuscript with one of the reviewers who provided the listed reports. This is part of the Nature Communications initiative to facilitate training in peer review and to provide appropriate recognition for Early Career Researchers who co-review manuscripts.

Version 2:

Reviewer comments:

Reviewer #5

(Remarks to the Author)
I co-reviewed this manuscript with one of the reviewers who provided the listed reports. This is part of the Nature Communications initiative to facilitate training in peer review and to provide appropriate recognition for Early Career Researchers who co-review manuscripts.

**Open Access** This Peer Review File is licensed under a Creative Commons Attribution 4.0 International License, which permits use, sharing, adaptation, distribution and reproduction in any medium or format, as long as you give appropriate credit to the original author(s) and the source, provide a link to the Creative Commons license, and indicate if changes were made.
In cases where reviewers are anonymous, credit should be given to 'Anonymous Referee' and the source.
The images or other third party material in this Peer Review File are included in the article's Creative Commons license, unless indicated otherwise in a credit line to the material. If material is not included in the article's Creative Commons license and your intended use is not permitted by statutory regulation or exceeds the permitted use, you will need to obtain permission directly from the copyright holder.
To view a copy of this license, visit https://creativecommons.org/licenses/by/4.0/

**Reviewer #1 (Remarks to the Author):**

This paper by Lobeto et al. investigates high energy swells generated in the Southern Pacific Ocean and reaching the eastern coast of the Americas, based on a 45-year numerical hindcast. The paper is well-written and organized, the figures are clear and of very good quality. However, while the method used to partition wave spectra and isolate southwester swells reaching the coast is innovative, the validation of the wave hindcast is not sufficiently detailed and raises important questions. As a consequence, I do not think that the paper can be accepted in its present form and I encourage the authors to address the following important points and to resubmit a new version:

We thank the reviewer for the thorough and constructive review. We appreciate the positive assessment of the manuscript and the valuable comments provided. In the revised version, we have carefully addressed all major and minor points raised, improving the validation of the hindcast, clarifying methodological choices, refining the interpretation of results, and correcting or expanding specific statements where necessary. We believe that these changes have substantially strengthened the manuscript and improved its clarity and robustness.

First, it is well-known that ERA5 winds have strong negative biases at high winds (over 20% for 25 m/s), which can lead to strong negative biases when modelling storm waves if no correction is considered. For instance, Alday et al. (2021) applied a 5% correction above 20 m/s combined to a tuning to 1.75 of the wave growth parameter in the wind input source term (so called “Betamax”). More recently, Gaffet et al. (2025) applied a quantile-quantile correction derived from a comparison between ERA5 winds and wind estimates from satellite altimeters. In the present study, the authors do not mention any bias correction while using ERA5 winds in their hindcast, which questions the reliability of their hindcast, namely for extreme sea states, on which this study is focused.

We thank the reviewer for this constructive comment and for the references provided. As the reviewer correctly points out, ERA5 winds are known to exhibit negative biases in certain regions, particularly at high wind speeds. Nevertheless, the following points have determined our configuration choices:

1. The use of ERA5 as forcing for wave hindcast development currently represents the state of the art, as supported by the two references kindly provided by the reviewer.
2. While we acknowledge the added value of the corrections proposed by Alday et al. (2021) and Gaffet et al. (2025), our hindcast was developed following a strict

calibration procedure based on the methodology of Stopa (2018), using satellite altimetry data. Although this methodology does not specifically target extremes, it provides the Betamax value that minimizes the overall bias of Hs at the global scale, balancing regions with positive and negative biases. Following its application, we obtained an optimal Betamax of 1.46, very similar to the value reported by Gaffet et al. (1.43).

3. It should also be noted that this global hindcast was developed prior to the availability of the study by Gaffet et al. (2025). Furthermore, while we initially considered applying the Betamax correction proposed by Alday et al. (2021), preliminary tests revealed undesirable positive biases under moderate conditions, as also discussed by Gaffet et al. (2025). For this reason, we opted to retain the standard calibration procedure, which provides more stable results for the general purposes of this hindcast, even at the cost of potential biases in some regions under extreme wind conditions.
4. A global validation of the hindcast has been performed using both satellite altimetry and buoy measurements. We will elaborate on this validation in the following response.
5. Although a potential negative bias in extreme events could exist, affecting the individual magnitudes of such events, its impact on their occurrence should be limited. The proposed method, which applies a variable threshold to identify events until a fixed number is reached, ensures that the events are detected even if their intensities are lower than their theoretical values. Consequently, we believe this issue should not significantly affect the sign of the trends, the correlations identified, or the seasonal variability in event occurrence.

That said, we fully agree with the reviewer that it is important to explicitly acknowledge in the manuscript the potential underestimation of storm magnitudes resulting from the use of ERA5 winds. This clarification has been included in the revised text.

L408-416: *"Additionally, it must be acknowledged that, although the hindcast used in this study—forced with ERA5—has been carefully calibrated and validated against both buoys and altimetry, the use of this forcing may still lead to some underestimation of wave conditions associated with extreme winds[78,79]. While this could affect the absolute magnitudes of the events, it should not compromise the main conclusions of this study. The applied methodology, which uses a variable threshold to identify events until a fixed number is reached, ensures that events are consistently detected even if their intensities are slightly underestimated. Together with the consideration of a sufficiently large sample of events each year, this provides robustness against systematic biases, ensuring that the detection of trends, interannual variability, and seasonality remains reliable."*

References:

- Stopa, J. E. (2018). Wind forcing calibration and wave hindcast comparison using multiple reanalysis and merged satellite wind datasets. Ocean Modelling, 127, 55-69.

Related to the previous issue, the validation of the wave hindcast is limited to a few buoys, all located in the same area (~central California). I am sure that plenty of wave buoys are available off the coasts of Central and Southern America. The authors should demonstrate that their numerical hindcast is accurate in regions where energetic waves reach the coast.

We thank the reviewer for highlighting this important point. We fully agree that a robust validation of the hindcast is essential to ensure the credibility of the results. The wave hindcast employed in this study is a new global dataset developed at IHCantabria, based on previous products (Perez et al., 2017, Reguero et al., 2012), using WAVEWATCH III forced with ERA5 winds, as mentioned in the manuscript. The hindcast was carefully calibrated following the methodology of Stopa (2018) using satellite altimetry, and validated globally against both satellite and buoy data.

Although a dedicated article describing this hindcast is currently in preparation, to provide transparency and robustness to the present study we have included in the Supplementary Material: (i) a detailed description of the model configuration, (ii) the calibration procedure, and (iii) the validation results, with a focus on the Pacific Ocean. As shown in Figure SM13, biases with respect to altimetry are very small, particularly in the study region where they remain below ±0.1 m. It is worth noting that, as in other hindcasts (e.g., Alday et al., 2021; Perez et al., 2017), biases in the generation areas of SO swells tend to be positive. RMSE values are well controlled, generally below 0.3 m in the study region and below 0.4 m in the generation areas. Correlations are very high (>0.95 in most of the Pacific Ocean), with the only exception being equatorial areas, where values are around 0.8 or higher.

Comparisons with buoy data also show very good performance. At the 95th percentile of Hs, biases remain below –1 m, and in most cases below –0.5 m, with an overall mean of –0.4 m across all buoys. This indicates a tendency toward underestimation, although the magnitude of the bias remains limited. Biases in mean and peak periods remain below 1 s, generally showing a positive sign (overestimation). We also note that most long-term buoys are located in the Northern Hemisphere, which prevents a complete assessment of biases for Southern Ocean swells.

Regarding the specific validation of Southern Ocean swells, this is inherently complex, as it requires spectral data of sufficient length and quality. We explored both the NOAA and

CDIP buoy databases. We selected CDIP spectra because they provided higher quality for this analysis. We made extensive attempts to include NOAA buoys in the validation, since a few of them provide data along other parts of the American coast. However, the results were very inconclusive, most likely due to issues related to the spectral reconstruction of these buoys. At first, one might attribute these discrepancies to model errors, but this was ruled out after confirming that NOAA buoys located near CDIP stations, which otherwise yielded very good results, still showed noisy and inconclusive comparisons. For this reason, we ultimately relied on CDIP spectra. Among these, we selected five buoys with sufficiently long records in affected regions, although most were clustered in a single area (coast of California). No buoys in the Southern Hemisphere outside of generation zones met the required standards.

Moreover, the swell identification algorithm requires clean and continuous spectral data, a condition often not met by buoy spectra. Therefore, applying the algorithm directly to buoy-derived partitions proved difficult to interpret reliably in terms of results. Instead, we adopted an alternative validation approach based on equivalent significant wave heights reconstructed from the directional and frequency sectors associated with Southern Ocean swells at the selected buoys. This approach produced robust results, especially considering the complexity of the comparison.

Finally, we emphasize that while systematic biases may exist, they should not alter the overall behavior of the results. By considering a sufficiently large sample of events, the influence of potential biases is reduced in the aggregated metrics, preserving the detection of positive trends, interannual variability, and seasonality. We also now explicitly acknowledge the limitations of ERA5 forcing in the revised manuscript (please see previous comment).

References:


- Stopa, J. E. Wind forcing calibration and wave hindcast comparison using multiple reanalysis and merged satellite wind datasets. Ocean Model (Oxf) 127, 55–69 (2018).
- Perez, J., Menendez, M., & Losada, I. J. (2017). GOW2: A global wave hindcast for coastal applications. Coastal Engineering, 124, 1-11.
- Alday, M., Accensi, M., Ardhuin, F., & Dodet, G. (2021). A global wave parameter database for geophysical applications. Part 3: Improved forcing and spectral resolution. Ocean Modelling, 166, 101848.
- Reguero, B. G., Menéndez, M., Méndez, F. J., Mínguez, R., & Losada, I. J. (2012). A Global Ocean Wave (GOW) calibrated reanalysis from 1948 onwards. Coastal Engineering, 65, 38-55.

In section "Historical trends", L229-232, the authors extrapolate the trends from their hindcast to suggest that wave power will keep on increasing in the future: why that? This is a very strong statement, that should absolutely be supported by wave climate change scenarios. Indeed, several studies did not show any increase in this area for 2100 (e.g. Hermer et al., 2013) while it is not clear that the increase found at low latitude in the Pacific by Lobeto et al. (2022, figure 3) will result in increased wave power along the coast of the Americas. Also, the reasons for such possible increased should be discussed.

We thank the reviewer for the comment. We believe it is an excellent suggestion to support this statement with previous studies on climate change projections. We also agree that it is more appropriate to do so in the Discussion section and to remove this strong statement from the Results.

Specifically, we will rely on the study by Lobeto et al. (2022), which is based on changes in directional wave spectra. Comparisons based on integrated parameters are not entirely adequate, since, as noted in Lobeto et al. (2021), the use of integrated parameters inevitably combines the signals of change from different wave systems. In regions where Southern Ocean swells are not the dominant system, the signal of increase may be masked by the changes in more dominant wave systems. Figure 6 in Lobeto et al. (2022) shows a positive change in the energy originating from the Southern Ocean, which propagates from the generation region toward the Northern Hemisphere. This increasing signal can be seen at every ocean point reached by Southern Ocean swells.

We have revised the text accordingly, adding this reference to support the statement while consistently framing it in potential terms, since, as the reviewer notes, this is a very strong claim that requires caution.

Therefore, the statement mentioned by the reviewer has been reformulated as follows:

L257-259: *"This result indicates a sustained increase in wave power over the last decades for the most energetic SO events affecting these coasts throughout the year."*

In addition, the Discussion has been revised as follows:

L346-361: *"Another reason why these events require considerable attention is their long-term evolution. Previous studies have assessed the historical and projected trends in wave height in the SO. However, outside the generation regions, studies relying solely on integrated parameters may mask the signals of SO swells*[67]*. This occurs because the change signal in the integrated parameter also combines the contributions of all individual systems. Consequently, along much of the studied coastline, where these swells are often*

*not the dominant system, their change signal is not captured by such studies, making it difficult to assess their past or future behavior. This study takes a step farther by examining the historical trend of these swells outside their generation zones. A statistically robust positive historical trend along nearly the entire coast of the Americas has been observed, affecting several indicators, including wave height, period and power. Considering the origin of these swells, our results are consistent with previous studies based on altimetry[68,69] and hindcast[70,71] data, which reported increases in mean and high-percentile* $H_s$ *in the SO. Moreover, the agreement in the sign of change with projected changes in* $H_s$ *reported in earlier works[72–75] in this ocean region suggest that a continued increase in the future is likely. Most importantly, these findings are also in line with prior conclusions drawn from wave spectra in climate projections[24,67], which showed a robust increase in SO swell energy along all points where these swells propagate including the west coast of the Americas by the end of the century."*

References:


- Lobeto, H., Menendez, M., & Losada, I. J. (2021). Projections of directional spectra help to unravel the future behavior of wind waves. Frontiers in Marine Science, 8, 655490.
- Lobeto, H., Menendez, M., Losada, I. J., & Hemer, M. (2022). The effect of climate change on wind-wave directional spectra. Global and Planetary change, 213, 103820.


I have also identified the following minor issues:

-L17: this sentence is ambiguous, please rather state "SAM+ phases are associated with more energetic swells.

Thank you for the comment. We have reworded the sentence accordingly.

L16-17: *"The Southern Annular Mode exerts a strong influence, with its positive phase (SAM+) being associated with more energetic swells."*

-L35-40: Please note that waves with larger Tp experience more shoaling and hence result in larger wave height at breaking compared to shorter-period waves with a similar amount of energy in deep water. Also, it would be worth mentioning that distant swells usually have narrower spectra and hence drive large infragravity waves, which can have a key contribution to marine flooding.

Thank you for the suggestion. We have changed the text accordingly:
L47-53: *"Additionally, waves with longer periods experience more shoaling than shorter waves transporting a similar amount of energy, resulting in a higher wave height at breaking and a larger release of energy upon dissipation. Moreover, distant long mature swells usually exhibit narrower spectra, which enhances the generation of infragravity waves. The height of these infragravity waves, released through the bound-wave mechanism, and for which a correlation with the product of the wave height and the squared period of the generating waves has been found[12,13], may also be relevant and capable of damaging coastal zones[14]."*

-L72: it depends on where you close the Indian Ocean but I doubt that swells generated in the Indian Ocean can reach the eastern coast of America.

Thank you for the comment. If we consider the eastern boundary of the Indian Ocean to be roughly at the eastern coast of Australia and assume great-circle path trajectories, our tracking algorithm indicates that some strong swells reaching the west coast of the Americas can indeed originate in the eastern part of the Southern Indian Ocean. This conclusion is also supported by previous studies: Pérez et al. (2015) showed, for two locations along the western coast of the Americas, that part of the incoming energy could be traced back to the Southern Indian Ocean (their Figs. 7 and 8). Similarly, Lemos et al. (2021) illustrated in their Fig. 5 the projected changes in swells generated in the Southern Indian Ocean across different ocean basins, showing that these swells reach our study coast. In addition, following this latter work, we present here a figure not included in their publication that shows the absolute number of swell events per grid point departing from the Southern Indian Ocean.

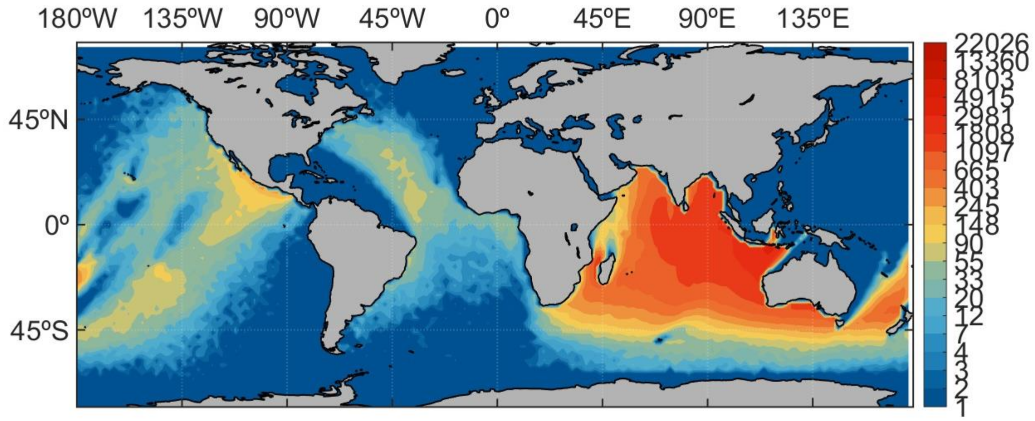


References:

- Pérez, J., Méndez, F. J., Menéndez, M., & Losada, I. J. (2014). ESTELA: a method for evaluating the source and travel time of the wave energy reaching a local area. Ocean Dynamics, 64(8), 1181-1191.

- Lemos, G., Semedo, A., Hemer, M., Menendez, M., & Miranda, P. M. (2021). Remote climate change propagation across the oceans—the directional swell signature. Environmental Research Letters, 16(6), 064080.

-L84-86: but this is the only area where you present a validation of your wave numerical hindcast.

Thank you for the comment. We agree with this observation. We have now included a more detailed validation, as discussed in our previous responses.

-L117: it is obvious that a wave model with a 0.25 km resolution cannot represent wave breaking.

Thank you for the comment. We agree and have reworded the sentence to avoid stating the obvious while keeping the rationale for the selection of the points.

L133-135: *"These points are representative of the entire coastal area while ensuring sufficient distance from wave-breaking shallow water zones."*

-L132: I think that this is always the case.

Thank you for the comment. We agree and have revised the sentence to clarify that the stability of wave direction is a general feature of swell events, with observed variations mainly reflecting the spatial evolution of the generating events in the Southern Ocean.

L152-153: *"The wave direction remains stable during swell events, with any observed variations most likely reflecting the spatial evolution of the generating events in the SO[55]."*

-L145: "climate-related anomalies" is not clear to me.

Thank you for the comment. We have clarified this point by specifying that we refer to large-scale climate variability modes such as ENSO.

L164-165: *"Other factors, such as interactions with other dynamics (e.g., tides, large-scale climate variability modes such as ENSO) and existing local coastal exposure, also play crucial roles."*

-L272-274: it would be worth reminding why distant long period waves represent a higher hazard compared to normal swells.

Thank you for the comment. We have expanded the explanation to emphasize that distant long-period swells are particularly hazardous not only because long periods enhance shoaling and run-up, but also because their narrow spectra favor the generation of infragravity waves, both of which contribute to coastal flooding.

L302-305: *"The combination of relatively high wave heights and long periods can make these swells hazardous, as their long periods enhance shoaling and run-up while their typically narrow spectra promote the generation of infragravity waves. Therefore, these events can drive significant water level increases at the coast, leading to coastal flooding and severe erosion (Table 1)."*

-L335-344: several partitioning algorithms are available, could you please justify why you used one based on Tp?

Thank you for the comment. As the reviewer notes, several algorithms exist for tracking swells. Our approach builds on the spectral partitioning framework of Portilla et al. (2012) and introduces a novel aspect by adapting it to the identification of swell events from spectral partition time series of Tp at specific coastal locations. In contrast to studies such as Delpey et al. (2010), which reconstruct basin-scale swell fields using spatially continuous spectral data and group velocity formulations, our methodology operates directly on partitioned time series at a single location. This improves computational efficiency and avoids the need for full spatial fields, making the method better suited to our focus on coastal impacts.

We have modified the text to include references to alternative algorithms and to clarify why we selected this approach.

L507-509: *"The approach for isolating swells is based on previous works*[53–55]*. Unlike space–time tracking methods developed to reconstruct basin-scale swell fields*[85]*, it is applied here to identify and characterize swell events directly from time series at individual coastal locations."*

References:

- Portilla-Yandún, J. Storm-source-locating algorithm based on the dispersive nature of ocean swells. Avances 4, (2012).

- Delpey, M. T., Ardhuin, F., Collard, F. & Chapron, B. Space-time structure of long ocean swell fields. J Geophys Res Oceans 115, (2010).

-L403: the wave energy corresponds to the wave height squared, therefore "proportionnal" is inadequate.

Thank you for pointing this out. We have revised the sentence to more accurately reflect the relationship between significant wave height and the spectral energy density

L468-470: *"Significant wave height ($H_s$): This represents the average of the highest one-third of wave heights measured in a one-hour sea state and is directly related to the total energy density of the spectrum."*

-L501: please check "m0_alfa" in eq.2 and "m0_teta" L501.

Thank you. We have corrected the typo.

L573-578: *"For comparison, wave energy propagating in a direction within the directional range connecting the buoy locations to the SO via great circle paths was selected. Additionally, only energy with periods longer than 15 s was considered. Comparisons were made on the basis of the equivalent $H_s$ ($H_s^e$), which was calculated as follows:*

$$H_s^e = 4.004\sqrt{m_{0\alpha f}} \qquad \text{Eq. 2}$$

*where $m_{0\alpha f}$ is the zeroth-order moment of the wave energy spectrum within a specified directional range $\alpha$ and frequency range f."*

-L521: here I do not understand, if you run your own model, you can output wave spectra and compute all the period you want, no?

Thank you for the comment. This point indicates that the parameter was not stored in our hindcast and is therefore not available for any period. A hindcast of this length is computationally very demanding, and practical constraints require decisions on which parameters can be archived, since it is not feasible to save the full spectral information. This is particularly relevant for partitions: in our case, we stored six (one sea and five swell partitions), which multiplies the storage requirements for each parameter by six. For that

reason, careful choices had to be made, and we decided to retain the peak period because of its potential utility for spectral reconstruction, but not the mean period. This decision was taken when the hindcast was originally developed a few years ago, and it is not feasible to rerun the entire simulation now solely to extract this additional parameter. We believe that the approach we propose adequately addresses this limitation with sufficient rigor.

Cited references:

Alday, M., Accensi, M., Ardhuin, F., and Dodet, G., 2021. A global wave parameter database for geophysical applications. Part 3: Improved forcing and spectral resolution, Ocean Model., 166, 101848, https://doi.org/10.1016/j.ocemod.2021.101848, 2021.

Gaffet, A., Bertin, X., Sous, D., Michaud, H., Roland, A. and Cordier, E., 2025. A new global high-resolution wave model for the tropical ocean using WAVEWATCH III version 7.14. Geoscientific Model Development 18 (6), 1929-1946, https://doi.org/gmd-18-1929-2025.

Hemer, M., Katzfey, J. and Trenham, C.E., 2013. Global dynamical projections of surface ocean wave climate for a future high greenhouse gas emission scenario, Ocean Modelling (70), 221-245, https://doi.org/10.1016/j.ocemod.2012.09.008.

Lobeto, H., Menendez, M., Losada, I. J. & Hemer, M. The effect of climate change on wind-wave directional spectra. Glob Planet Change 213, (2022).

**Reviewer #2 (Remarks to the Author):**

GENERAL SUMMARY

The manuscript "Southern Ocean swells pose a significant and growing threat to the Pacific coast of the Americas" assesses the characteristics of swells generated in the Southern Pacific Ocean (SO) once they've propagated to the Pacific coast of the Americas. Swell events are characterized based on different indicators (i.e., wave height, period, direction, and frequency of occurrence), and they are evaluated by their temporal variability across multiple time scales, with an emphasis on long-term trends. The main findings indicate that waves are more energetic close to their generation area, in the southern Pacific Ocean, gradually decreasing their wave height and period from south to north along the Americas. Results also indicate that SO swells varied across seasons, years, and decades, and interannual variability, driven by the Southern Annular Mode (SAM) is the dominant time scale influencing wave power variations across the Pacific coast of the Americas. The analysis, which uses a novel framework to isolate swell events based on spectral data, offers an alternative framework to analyze large SO swells individually and in detail to more traditional approaches that use the basis of total integrated parameters. Overall, the authors do an excellent job of demonstrating the influence of SO swells over the full-length of the Pacific coast of the Americas, and their main conclusions are supported by the high-level framework adopted by the study. Still, there are some areas for further improvement, specifically to clarify and highlight the implications of their findings in a broader context, refine the descriptions of swells related to impacts, and refine/better explain some of the methodological approaches.

We sincerely thank Reviewer #2 for the thorough and constructive review. We greatly appreciate the positive evaluation of the manuscript and the recognition of its novelty and relevance. In the revised version, we have carefully addressed all the major and minor comments raised, clarifying the implications of our findings in a broader context, refining the descriptions of potentially hazardous swells and their impacts, and providing further detail on the methodological aspects as requested. We believe that these changes have significantly improved the clarity, robustness, and overall quality of the manuscript, and we are grateful for the reviewer's valuable input, which has been essential in strengthening this work.

Specific Comments

The manuscript addresses a novel and relevant topic, which is highly significant because SO-generated swells with high energy have the potential to cause hazards. I liked how the

authors tried to define the impact of the swell events by cross-referencing them with reported hazards along the Pacific coast of the Americas, rather than broadly claiming that because they're "extreme" they are impactful. However, I believe the title overstates the relationship the authors describe. First, the authors compile a list of events that cause damage in Table 1. It is unclear whether these events are related to SO swell in the articles, or whether the authors found damaging events and then later related them to events they characterized as swell. If the latter is the case, they probably shouldn't be described as "numerous SO swell events" causing some form of damages (line 74-75). If the reports directly relate the damage/impacts to SO swell, then that should be more clearly stated.

Thank you very much for your comment and for your positive assessment. In this case, we examined the reports for damaging events caused by extreme wave conditions. These reports specify the damages attributed to wave activity upon arrival at the coast. While not all of them explicitly mention Southern Ocean swells (although many do), we believe this is due to the highly technical and specific nature of that terminology; instead, the reports often refer more generally to intense or high wave conditions. Although, based on expert judgment, it seemed reasonable to assume that these damages were associated with such events, we verified this by cross-checking the reported event dates with wave climate data from different sources, such as buoys and hindcasts. In other words, the corroboration of these swells as Southern Ocean swells was not based on the method proposed later, but rather on an empirical analysis of wave data, including integrated parameters (e.g., wave height, direction) and spectral information such as directional spectra or partitions. This therefore supports the statement that numerous Southern Ocean events have caused damage, since the damages were documented and we verified that they indeed correspond to this type of event.

Afterward, we designed the algorithm to identify them, and used the events listed in the table—those for which we were certain they were Southern Ocean events—to validate the method. The design of the method, given the nature of the data, was very challenging: whenever we succeeded in capturing some events, others tended to disappear from the sample, until reaching the optimal balance. Having this table available a priori was therefore essential to ensure the proper functioning of the method. We have revised the manuscript to clarify how the table was generated.

L94-97: *"While the origin was explicitly specified in the reports for many of the listed events, for the remaining ones it was corroborated by analyzing the available wave data (buoys and hindcasts), particularly the direction and period of the sea state at the time of swell arrival."*

With regard to the title, and taking into account the above discussion, we have modified it to make it less categorical while still consistent with our results. We have identified the Southern Ocean swells and examined their trend. In addition, we have confirmed that these swells have already caused severe damage along these coasts and that their threat is increasing over time.

Southern Ocean swells pose an important and growing threat to the Pacific coast of the Americas

Following on, it is noted that the events don't necessarily reflect all the countries affected or damage caused, but the statement is made that "The collected information shows that the impact of these events is more frequent on the coasts of South and Central America." I don't believe the assessment done is rigorous enough to make this statement – and if this table is part of the results, more detail must be provided on how events were selected, the methods for finding the events, etc. in order to ensure the selection criteria does not bias the results. If the point of this table is to show that some of the SO swell events may in fact cause impacts, this could be brought up later once swells are characterized.

For example, Cross referencing – 23 swells compared to 900 at each location – isn't a very large sample size and it doesn't necessarily indicate that all the events being defined as powerful do cause impacts. The manuscript would benefit from more explicitly defining how the cross-reference method was implemented in the methods section.

We thank the reviewer for this insightful comment. The statement in question was originally based on an extensive search through scientific literature, disaster reports, and news archives, which suggested that damaging swell events appeared to be more frequently reported in Central and South America than in North America. However, we acknowledge that such evidence is not sufficiently robust and may be affected by reporting biases or inconsistencies in documentation practices across regions.

The reviewer is absolutely right in pointing out that this kind of claim cannot be substantiated solely through external searches. Indeed, the actual occurrence and impact of events should be addressed using the methodology proposed in this study, once the swell events have been objectively characterized.

The table was intended solely as a means to illustrate the importance of these swells along the study coast and for validation, and not as part of the study's results. In light of this, we have revised the introduction and removed the statement to avoid any potential confusion or unsupported inference. We appreciate the reviewer's input, which has helped to improve the clarity and scientific rigor of the manuscript.

L88-101: *"The events listed here were found in previous studies, reports, digital newspaper archives or disaster databases, as referenced in the last column of Table 1. While the origin was explicitly specified in the reports for many of the listed events, for the remaining ones it was corroborated by analyzing the available wave data (buoys and hindcasts), particularly the direction and period of the sea state at the time of swell arrival. Although this information does not necessarily reflect all the countries affected and/or all the damage caused, it provides a clear indication of the severity and relevance of these events. Additionally, the recorded arrival date may vary slightly across different countries. In most cases, the energetic swells from the SO led to coastal flooding, resulting in damage to infrastructure, homes, and personal property, as well as to the disruption or complete interruption of activities and consequent economic losses. Furthermore, these strong swells occasionally cause the capsizing of vessels and the drowning of individuals. Exceptionally energetic events can reach very high latitudes and still cause severe impacts, such as those in 2011 on the coasts of North America (see Event no. 8 in Table 1). On some coasts, these swells can also be appreciated for their recreational value, as they create optimal conditions for surfing[51], for example."*

Regarding the cross-reference, the findings in the table were later used to validate the method, with the aim of verifying that the proposed approach was capable of identifying all the events previously documented there.

On that note, some of the jargon surrounding the hazardous nature of these events needs to be more clearly articulated. For example, on Line 144 – the swells are described as "potentially damaging events" and this terminology is used throughout the manuscript, but in other instances, hazardous event terminology with more certainty is used, like in line 302 with "hazardous SO swells". At the beginning of the "Discussion & Conclusions" section (line 257), swells in the SO are mentioned to be important to coastal hazards, but this is not always the case. These swells can lead to hazards but given the right circumstances! This is contradictory to what is mentioned in the introduction regarding the US Pacific coastline, where these swells have "less ability" to cause impacts and may be linked to recreational activities, like surfing.

Thank you very much for the comment. As the reviewer correctly points out, these events can indeed give rise to hazards under appropriate conditions. For this reason, we have consistently referred to them throughout the manuscript as potentially damaging events, as specified in L162: *"Nevertheless, these events are referred to as potentially damaging events, as their occurrence alone does not necessarily result in damage".* In line with the reviewer's suggestion, we have taken care to ensure consistency and avoid contradictions

across the entire text. Specifically, we have identified instances where expressions such as the one noted by the reviewer were used and replaced them with wording consistent with the rest of the manuscript.

L287-288: *"Swells generated in the SO <u>may pose important coastal hazard</u> for many regions worldwide, particularly along western continental coastlines"*

L302-303: *"The combination of relatively high wave heights and long periods <u>can make</u> these swells hazardous"*

L332-333: *"<u>Potentially</u> hazardous SO swells are more likely to occur during austral winter…"*

L386-388: *"The findings of this study can assist in addressing critical aspects, such as identifying the time of year when <u>potentially</u> hazardous SO swell events are more frequent."*

Another topic I'd like discussed a bit more is whether wave transformation processes and bathymetry may impact coastal results. It states in the manuscript that coastal points were sufficiently far away from wave breaking, but what about wave shoaling? The majority of coastal points are in deep water, but a few locations are located in 100 – 600 m of water, and with longer period waves (e.g., >14s) you could calculate that the wave base could in fact interact with the seafloor, beginning wave shoaling processes. One of the coastal points in the pacific northwest of the US has very different results than its surrounding geography – and I wonder if that may be part of the reason.

We thank the reviewer for raising this important point. To assess potential shoaling effects, we compared the depth at each coastal point with the deep-water criterion ($h/L > 0.5$, i.e., $h > L/2$). For swell periods of 14–20 s, all but two points (P2 at 208 m and P17 at 136 m) satisfy this condition. At P2, the relative depth is $h/L \approx 0.68$ for T=14 s (deep water) and ≈0.33 for T=20 s, while at P17 it is ≈0.44 for T=14 s and ≈0.22 for T=20 s. These values fall within the intermediate-depth regime, meaning that long-period swells may begin to interact with the sea floor. However, both locations remain far from the shallow-water threshold ($h/L < 0.05$), so any shoaling effect is expected to be very limited. This may partly contribute to local deviations such as those observed in the U.S. Pacific Northwest, while the broader regional conclusions remain unaffected.

We have added this discussion to the manuscript:

L135-136: *"At the same time, the points are located at depths sufficient to avoid, even for long-period waves ($T_p$ longer than 14 s), any pronounced shoaling processes."*

Along those same lines, the depth of the buoys can be quite different when compared to the equivalent hindcast point. For example, P4 is at 1906 m and an equivalent in location buoy at 123 degrees W is at 600 m – how different can the partitioned, maximum Hs be between these two?

We thank the reviewer for raising this point. For the model–data comparison used in our validation, we did not use the 22 coastal analysis points. Instead, for each buoy we selected the nearest hindcast grid point providing directional spectra and compared the incoming SO-swell energy along the great-circle sector connecting the buoy to the Southern Ocean, filtered to periods >15 s. The equivalent significant wave height was computed consistently from both buoy and model spectra, and the same swell-isolation algorithm was applied at buoy locations. This procedure is already described in Methods – Validation.

For completeness, the table below lists the buoy depths together with the corresponding hindcast point depths used in the comparison:

| ID | Longitude [º] | Latitude [º] | Depth [m] | Depth hindcast [m] |
|---|---|---|---|---|
| 067 | -119.892 | 33.226 | 315 | 861 |
| 222 | -121.497 | 34.767 | 650 | 404 |
| 029 | -123.465 | 37.940 | 600 | 410 |
| 157 | -122.104 | 36.335 | 369 | 1582 |
| 071 | -120.780 | 34.452 | 550 | 604 |

Although depth differences do exist—most notably for buoy CDIP 157—all sites and their corresponding hindcast points are located in deep water for the SO-swell periods considered (typically 15–22 s). Under these conditions, depth-induced shoaling or refraction effects on the partitioned maximum Hs at the event peak are expected to be minor compared to other sources of mismatch, such as spatial separation, grid resolution, or spectral partition labeling. This is precisely why buoys at sufficiently large depths were selected and why the evaluation was focused on SO-swell energy during identified events.

L568-572: *"To validate the incoming wave energy propagating from the SO in the modeled dataset, observations from five spectral buoys managed by the Coastal Data Information Program (CDIP) were used. For each buoy, the nearest hindcast grid point providing directional spectra was selected for comparison. All the selected buoys are located at depths sufficiently large to avoid wave-breaking related shallow-water processes. Table SM2 summarizes the key characteristics of the buoys."*

I'm also wondering how many of the same swell events impact large stretches of coastline. How many of the top 20 events each year also occur in neighboring stations? This would be a cool analysis to complete if not already done.

We thank the reviewer for this suggestion. Our analysis is intentionally based on the 20 most powerful swell events per year at each location, precisely to ensure robustness against the potential noise and inconsistencies that can arise when tracking individual events across multiple coastal points (e.g., event merging, truncation of events, or overlap with other systems). As discussed in the manuscript, this strategy ensures a consistent dataset across the entire coastline and minimizes the influence of unresolved noise, allowing us to provide a comprehensive and robust characterization of extreme swell conditions. Attempting to track the same event across stations would represent a different, event-by-event philosophy, which falls outside the scope of the present work.

Nonetheless, we agree that the alongshore coherence of Southern Ocean swells is a highly relevant topic and a natural avenue for future research. In fact, this forms part of our planned future work, where we aim to investigate the spatial footprint and compound impacts of selected extreme swell events.

The authors define "frequency of occurrence" based on the 20th-ranked annual event as the threshold to count annual exceedances. How sensitive are results in the trend analysis to the chosen threshold?

We thank the reviewer for this important observation. In our framework, the threshold is not arbitrarily chosen but is site-specific and time-invariant, defined as the maximum, over 1979–2023, of the power of the 20th-ranked annual event at each site. This choice was intentional for two reasons: (i) it anchors the indicator to a high, fixed severity level that is comparable across years and locations, and (ii) it guarantees full dynamic range in the annual exceedance counts (0–20) without saturating the metric (too many exceedances if the cut were low) or emptying it (too few if the cut were extreme). By construction, the threshold-setting year reaches 20 exceedances, while all other years remain ≤20, which stabilizes trend estimation.

As a consequence, the metric is structurally constrained and does not depend on tuning the threshold: any alternative definition would undermine the comparability and consistency of the indicator. In this sense, a classical sensitivity analysis is not applicable,

because the definition of "frequency of occurrence" is uniquely tied to the chosen framework rather than to an adjustable parameter. To avoid ambiguity, we have clarified this rationale in the Methods section.

L608-616: *"Frequency of occurrence: This indicator represents the number of events – out of the 20 events selected per year at each point – that exceed a certain threshold. To ensure that the threshold was adaptable to varying wave conditions across the study locations, it was defined as the highest value of the 20$^{th}$-ranked annual event by power over the 45-year period analyzed. This definition (i) anchors the indicator to a fixed, high-severity level that remains comparable across years and locations, and (ii) guarantees the full 0–20 dynamic range in annual exceedance counts, avoiding both saturation (too many exceedances if the threshold were set lower) and emptiness (too few exceedances if set higher). By construction, the year that sets the threshold attains 20 exceedances, whereas all other years are ≤20."*

Can you justify the approach for ENSO calculations? Are they occurring over the calendar year or over some wave year focused on not breaking up the "wave season"? If you have a calendar year that switches from El Nino to La Nina like 1983 or 2016 – would the annual average accurately characterizes the index and the extremeness of the ENSO phase?

We thank the reviewer for this important comment. In the original submission, ENSO was aggregated on a calendar-year basis (January–December) for consistency with the analysis conducted for SAM and, more generally, with the rest of the manuscript. However, as the reviewer correctly points out, this definition may artificially mix different phases of the same ENSO event across the year boundary. ENSO events typically grow during boreal summer, peak in boreal winter (DJF), and decay in boreal spring (MAM). In years such as 1982–83 and 2015–16, very strong El Niño peaks occurred in DJF but were followed within the same calendar year by a rapid collapse and, in some cases, the onset of La Niña. A January–December average therefore combines pre-peak positive anomalies with post-peak neutral or negative anomalies, which does not adequately represent the ENSO forcing relevant for austral-winter (JJA) waves.

To address this, in the revised analysis we compute ENSO on an enso-year basis (May–April), so that the full cycle of growth, peak, and decay remains within a single year. We also tested alternative definitions, such as restricting the ENSO index to the months in which wave events occurred or weighting monthly ENSO values by event intensity, and obtained consistent results, none of which improved the relationship in a robust way.

In addition, to enhance the rigor of the article, we used the annual SAM Marshall index, calculated from the annual mean pressure fields, rather than the annual-averaged monthly

AAO index. The results slightly reduce the maximum correlations but improve the minimum correlations.

Finally, in the first version of the manuscript we reported Pearson correlations to quantify the relationship between climate modes and interannual swell variability in the South Pacific. In the revision, we have adopted simple linear regression models. This provides a transparent statistical framework that allows us to compare results across indices and extend the analysis to models with more than one predictor. In the univariate case, the two approaches are mathematically equivalent: the regression slope corresponds directly to the correlation coefficient, and the associated $p$-values are identical.

We have modified the MS accordingly:

L216-223: *"Interannual variability is highly pronounced in energetic SO swells. The annual time series of $H_s$ at the target coastal points clearly reflects this variability (Figure SM5). To understand the patterns governing these variations, we analyzed their correlation with two climate circulation patterns characterized by climate teleconnection indices (see Methods). First, the correlation with the Southern Annular Mode (SAM) was examined via the observation-based Marshall index[59], which is associated with atmospheric circulation in the SO. Second, the correlation with the ENSO phenomenon was assessed via the multivariate ENSO index (MEI), which influences climate conditions in the Pacific, particularly in the intertropical region."*

L619-630: *"The correlation between changes in wave power and different teleconnection patterns was calculated using linear regression models (Eq. 4), which in the univariate case are mathematically equivalent to Pearson's linear correlation[87]. Annual indices were used to explore interannual variability through aggregated annual metrics. For SAM, we used the annual observation-based Marshall index[59]. For ENSO, we obtained annual values from the monthly MEI index using an enso-year definition (May–April)[88–91], ensuring that the full evolution of individual ENSO episodes (El Niño/La Niña) is contained within a single year, while keeping the main wave season (JJA) intact and not split across two different years.*

*The regression models were then fitted between these indices and the annual mean $P_{wp}$ of the 20 most powerful events at each target point.*

$$P_y = \alpha + \beta X_y + \varepsilon_y \qquad \text{Eq. 4}$$

*where $P_y$ is the annual mean $P_{wp}$ of the 20 most powerful events, $X_y$ is the annual index, $\alpha$ is the intercept, $\beta$ is the slope and $\varepsilon_y$ is the residual."*

We have also updated the description of the results to refer to SAM instead of AAO, and we have revised Figure 3 accordingly.

L224-233: *"The results indicate a strong correlation between the annual mean wave power of the selected events and the positive phase of the SAM, with all analyzed locations showing significant correlations at the 90% confidence level (Figure 3). During this phase, westerly winds in the Southern Hemisphere (SH) intensify and shift poleward, along with extratropical cyclones generated in this region[60,61], which likely contribute to increased wave power in events generated south of 40°S. These findings align with previous studies showing a strong correlation between the positive phase of this index and both mean and extreme wave heights in the SO and eastern Pacific Ocean[62–65]. The observed correlations along the Pacific coast of the Americas are particularly relevant north of the equator, consistently exceeding 0.5. The maximum values are found along the coast of Guatemala and the Southernmost coast of Mexico, ranging from 0.6 to 0.7. South of 5°S, the correlation values fall below 0.4 at most locations."*

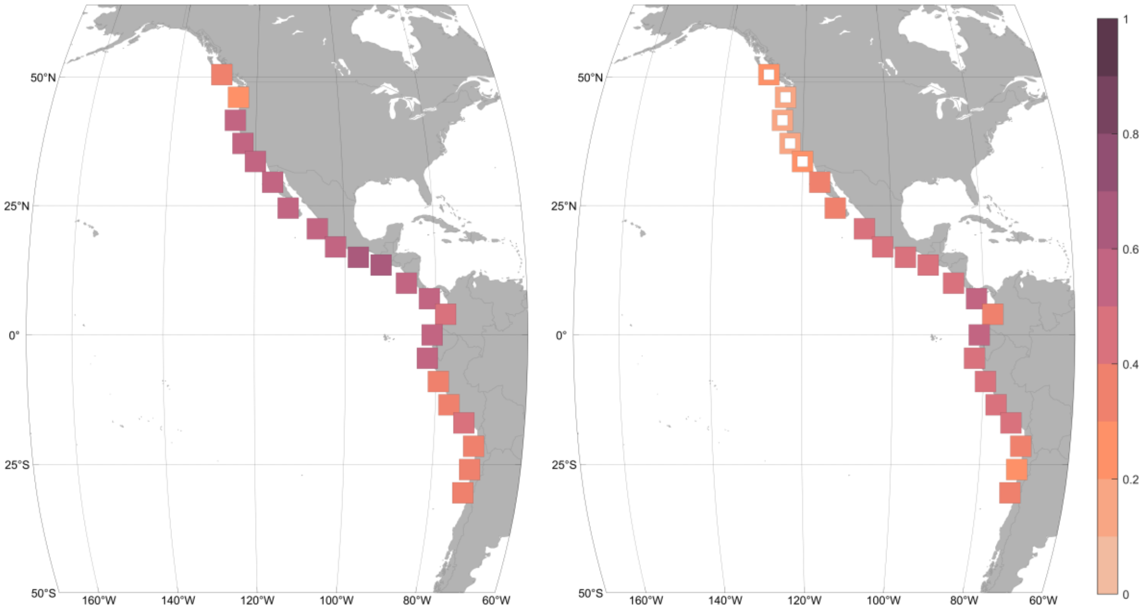


***Figure 1****: Left panel: Linear correlation between the annual mean wave power and the positive phase of SAM (Marshall Index). Right panel: Linear correlation between the annual mean wave power and the negative phase of ENSO (MEI). Filled squares indicate statistical significance at the 90% confidence level.*

Lines 556 to 557, describe the interannual variability in swell indicators. Does the interannual variability consider JUST 2 points (i.e., end-point method) from all the events for the 45 years? If that is the case, this maximum-minimum difference is potentially more representative of the "bounds of variability" within the dataset or what the maximum interannual variability could be in each location based on 45 years of available data. This

difference could potentially capture a maximum and minimum related to climate modes with decadal or multi-decadal periodicities, representing a difference that is not frequently experienced over the period of study, and thus, might not represent the typical/more frequent interannual variability of the region. If an end-point method is being considered, the potential large difference could not be frequently observed. Have the authors assessed how often a 60% variability is observed (reported in lines 296-298 at specific locations)? An end-point approach could potentially overestimate the dominance of interannual variability in wave power variability by capturing variations related to infrequent climate modes. To better represent typical interannual variability, one suggestion is to considering differences across all data points (consecutive or not) and then averaging them to provide a more representative variability of all potential climate modes influencing oscillations across the Pacific coast of the Americas.

We thank the reviewer for this insightful comment. We fully agree that an end-point approach may not adequately represent the variability typically experienced under normal conditions, as it can be strongly influenced by infrequent events.

In response, we have revised our analysis to adopt a mean-based approach, thereby avoiding potential overestimations related to end-point methods. Specifically, we retain the original definitions of seasonal and trend-associated variability, but we recalculate interannual variability using the Gini mean difference (GMD), which computes the average of all pairwise absolute differences among annual means. This metric provides a more representative measure of typical interannual fluctuations, as suggested by the reviewer.

To gain insight into the maximum interannual variations without relying on the end-point approach, which introduces additional uncertainties, we computed the normalized standard deviation of all pairwise differences between annual mean wave power values.

Accordingly, we have updated the Methods, Results, and Discussion sections, as well as Figure 5, to reflect the revised mean-based approach.

Methods:

L649-654: *"Interannual variability was calculated as the Gini mean difference (GMD)[99] of annual mean $P_{wp}$ values over the 45-year detrended period, normalized by the study period's mean value. The GMD corresponds to the average of all pairwise absolute differences among annual means, providing a representative measure of typical interannual fluctuations. Additionally, the normalized standard deviation of all pairwise absolute differences was computed as an indicator of the variability that may be introduced by interannual fluctuations."*

Results:

L275-284: *"Seasonal variability is the dominant timescale influencing wave power variations across the study coast. The magnitude of seasonal variations (normalized by the mean) varies significantly along the Pacific coast of the Americas, ranging from a minimum of 0-10% along the northernmost locations analyzed to a maximum of 25-30% along the Central American coast. Interannual variability ranks second in importance, with magnitudes generally between 10% and 20%, reaching their highest values along the coasts of North America. However, it is important to note that interannual variability itself exhibits a wide range, with the normalized standard deviation consistently exceeding 10% along both the Central American and North American coasts (Figure SM7).  Finally, the least pronounced variations correspond to long-term trends, which show results comparable to interannual variability in magnitude but remaining below 10% in the northernmost coastal sector."*

Discussion:

L327-345: *"This study provides a deeper understanding of when these events are most likely to occur and how their characteristics vary across different time scales. After analyzing the seasonal, interannual, and long-term variations in event power, the seasonal variability was identified as the time scale driving the most intense variations over the 45-year analyzed period. Seasonal changes in event power, show normalized mean values reaching 25–30% along the Central American coast. Not only do the characteristics of the events vary throughout the year, but their frequency of occurrence also differs markedly by season. Potentially hazardous SO swells are more likely to occur during austral winter, which coincides with boreal summer, which is the peak season for coastal recreational use in the NH, increasing exposure of coastal populations and infrastructure to wave impacts. The probability of occurrence also remains high in spring and autumn, with the lowest likelihood during boreal winter.*

*Interannual variations also exert a great influence on wave power variations. These interannual variations are closely linked to the influence of the SAM teleconnection pattern, with more intense events expected to occur during its positive phase. The analysis revealed that within different years during the study period, the mean relative differences in the annual mean power of these events reached 10–15% in South and Central America and exceeded 15% in North America (Figure 5), with standard deviations of the same order of magnitude, underscoring the high dispersion of interannual fluctuations in these regions. Therefore, the modulation of these events by large-scale climate patterns such as the SAM suggests that even in areas where SO swells are not the dominant system, climate variability can enhance their hazard potential."*

The authors acknowledge the importance of both the Southern Annular Mode (SAM) and the El Niño Southern Oscillation (ENSO) because of their correlation to wave power. This analysis could be potentially replicated but considering the interaction of these two climate modes during the same period. Positive SAM and negative ENSO are known to increase wave heights in the SE Pacific, as acknowledged by the authors. Have the authors considered doing so? This could be done with an interactive term and could potentially increase the correlation or reveal new findings regarding different combinations of phases.

We thank the reviewer for this constructive suggestion. To evaluate the potential combined influence of ENSO and SAM, we systematically tested linear regression models linking annual wave power to each climate index individually, as well as to both indices together. For clarity, here we present detailed results for point P10, where the sensitivity to both modes is particularly clear. We emphasize, however, that the overall behavior is consistent at other locations.

We first fitted univariate regression models, where annual mean wave power of the 20 most powerful swell events is expressed as a linear function of a single climate index:

$$P_y = \alpha + \beta X_y + \varepsilon_y$$

In this setting, the regression slope β quantifies the direction and strength of the relationship, while the variance explained is measured by the coefficient of determination $R^2$, which represents the fraction of variance in annual wave power captured by the fitted model. In the univariate case, $R^2$ is mathematically equivalent to the square of Pearson's correlation coefficient, and the associated p-value corresponds to the significance test for the slope β.

For ENSO alone, the model explains about 18% of the interannual variance at P10 ($R^2 = 0.18$). In contrast, SAM alone explains about half of the variance ($R^2=0.50$), confirming it as the dominant driver.

We then extended the analysis to a multiple regression model including both indices additively:

$$P_y = \alpha + \beta_1 ENSO_y + \beta_2 SAM_y + \varepsilon_y$$

This model explains slightly more variance than the SAM-only model, with $R^2 \approx 0.54$.

Finally, we tested a model including an interaction term:

$$P_y = \alpha + \beta_1 ENSO_y + \beta_2 SAM_y + \beta_{12}(ENSO_y \cdot SAM_{y}) + \varepsilon_y$$

The interaction term was not statistically significant and the model did not improve the explained variance (R2≈0.54, virtually identical to the additive case).

All analyses were carried out using ENSO-year definitions (May–April) for both ENSO and SAM, to ensure consistency between indices. Importantly, SAM results remain essentially unchanged under this adjustment.

In summary, at P10—and consistently across other locations—the results show that SAM alone already captures about half of the interannual variance in swell energy, ENSO alone explains only a modest fraction, and the additive model provides a very small improvement over SAM-only. The interaction term is not significant and does not improve model skill.

Given these outcomes, we do not consider the combined ENSO–SAM analysis to add substantially to the main conclusions of the manuscript. In the revised version, we will therefore include only a brief statement in the Results noting that combined models were tested and did not improve upon the SAM-only case, and that the interaction term was not significant. We believe that presenting detailed results would add unnecessary complexity distracting from the central findings. A more thorough exploration of joint ENSO–SAM effects on wave climate, while of interest, would require a dedicated study and falls beyond the scope of the present manuscript.

L241-243: *"Combined models including ENSO and SAM were also tested. The additive formulation produced only a minimal improvement over the SAM-only model, while the interaction term was not significant and did not enhance model performance (not shown)."*

The discussion and conclusions section could be strengthened if the authors highlight the implications of overlooking potentially hazardous secondary events, such as SO swells, in the context of changing frequency, especially because some locations do show significant increases. This is particularly evident from lines 287 to 290. This could also be strengthened if events of different frequencies were considered in the definition of this indicator; as previously mentioned, rarer or more frequent events could yield different results in terms of changes in the frequency of occurrence than the 20th-ranked event/98th percentile. In this section, findings related to changes in swell indicators associated with SAM in the NH could help emphasize the importance of large-scale climate patterns for projecting potential hazards, especially in the NH where the SO swells do not dominate the wave climate.

We thank the reviewer for this valuable comment. The discussion has been revised accordingly, as suggested by the reviewer:

L319-326: *"However, overlooking potentially hazardous secondary events, such as SO swells, could have severe consequences. Although they may appear as secondary components in the wave climate of certain regions, particularly in the NH, disregarding them could lead to an underestimation of coastal risk. These swells possess substantial energy or may exhibit notable directional deviations from dominant conditions, increasing the potential for significant impacts if adequate protective measures are not in place. This highlights the need for a comprehensive characterization of the wave climate that goes beyond traditional approaches based on integrated parameters, as these often mask the presence of remote swells[67]."*

L343-345: *"Therefore, the modulation of these events by large-scale climate patterns such as the SAM suggests that even in areas where SO swells are not the dominant system, climate variability can enhance their hazard potential."*

L367-371: *"Our findings also show that the frequency of energetic SO swells has significantly increased in several regions, which underlines their relevance as a hazard. Future work could extend this analysis to explore how different thresholds of event frequency, from more common to rarer extremes, alter the detected trends in occurrence, thereby providing a more complete picture of evolving coastal hazards."*

Regarding swell indicators, has the historical variability in other metrics, like variance and standard deviation, other than the mean been explored? The mean could be not experiencing significant trends, but other metrics could, and this would strengthen the characterization of the historical variability of SO swells.

We thank the reviewer for this insightful comment. Following the reviewer's suggestion, we have analyzed the trends in the annual standard deviation of event power, as well as of Hs and Tp. However, we did not obtain notable results: in all three cases, the fraction of grid points showing statistically significant trends remained below 20%. We acknowledge that a different methodological approach might be required to better capture variability between events, which is beyond the scope of the present study.

Trend calculation, page 12 – have the authors considered using a more robust method for assessing linear trends? Like Sen's Slope estimator, which is known to be more robust to outliers and non-normality in the data, which should be expected with the datasets used.

Following the reviewer's recommendation, we have slightly modified the methodology used to calculate the trends. Specifically, the trend magnitude is in the new version

estimated using the Sen slope estimator, which is less affected by outliers than a standard least-squares regression. Given the intrinsic noise in the data, we consider this a more robust choice for characterizing the underlying trend than previous methods based on ordinary least squares (OLS) regression.

For the assessment of statistical significance, we continue to rely on the Mann–Kendall test, as this binomial approach (Mann–Kendall for detection and Sen slope for magnitude) is widely adopted in the scientific literature. This combination provides a non-parametric and robust framework that is particularly well suited to environmental and climatic data.

Some relevant references that employ this approach are:

Erikson, L., Morim, J., Hemer, M., Young, I., Wang, X. L., Mentaschi, L., ... & Webb, A. (2022). Global ocean wave fields show consistent regional trends between 1980 and 2014 in a multi-product ensemble. Communications Earth & Environment, 3(1), 320.

Kliengchuay, W., Mingkhwan, R., Kiangkoo, N., Suwanmanee, S., Sahanavin, N., Kongpran, J., ... & Tantrakarnapa, K. (2024). Analyzing temperature, humidity, and precipitation trends in six regions of Thailand using innovative trend analysis. Scientific Reports, 14(1), 7800.

García-García, A., Cuesta-Valero, F. J., Miralles, D. G., Mahecha, M. D., Quaas, J., Reichstein, M., ... & Peng, J. (2023). Soil heat extremes can outpace air temperature extremes. Nature Climate Change, 13(11), 1237-1241.

We have revised the manuscript to adopt this new approach, incorporated the indicated references, and updated the figures accordingly.

L633-641: *"Trends in the different indicators were estimated using the non-parametric Sen's slope estimator to minimize the influence of outliers on the results. The statistical significance of the trend was assessed with the Mann-Kendall trend test (MK)* [92,93]*, which evaluates the presence of a monotonic upward or downward trend over time by testing if the slope of the estimated regression line differs from zero. The Sen–MK combination has been widely applied in the climate science literature, as it provides a robust, distribution-free framework for detecting and quantifying monotonic trends in environmental and climatic time series*[70,94,95] *where the assumptions of parametric methods are often not met. A trend was considered statistically robust when MK confirmed a significance level below 0.1, a threshold commonly used in climate studies*[94,96,97] *and consistent with conventions adopted in IPCC assessments*[98]*."*

The discussion and conclusions would benefit from incorporating a more explicit discussion of the implications of this study in a broader context. While the novelty of the work is clear, the manuscript frames the significance of results on a more regional basis does this have implications for other areas across the globe?

Thank you very much for the comment. We have revised the introduction and the discussion to clearly emphasize the global scope of the problem addressed in the article:

L77-90: *"Southern Ocean swells can affect both insular and continental coastlines across all ocean basins. They propagate thousands of kilometers to reach distant shores in the Pacific, Indian, and Atlantic Oceans, with the capacity to generate impacts in locations as diverse as low-lying atolls or continental margins. In island nations, a single event can produce widespread flooding[43,44], lagoon submersions[45,46], ecosystem damage[45,47], and economic losses equivalent to a large fraction of the annual Gross Domestic Product (GDP)[44,48], sometimes accompanied by community displacement and severe disruptions to livelihoods[43,48]. The existential nature of these impacts is expected to worsen with rising sea levels, threatening the long-term habitability of atoll islands[49]. In contrast, in continental margins impacts are generally not existential but translate into recurrent coastal erosion[2,28,29], flooding of communities[30,31], casualties, damages to infrastructure and disruption of maritime activities[1]. In particular, the Pacific coast of the Americas is one of the continental coastal regions in the world most affected by these events[1,50]. The western coasts of the American continents are fully exposed to energetic swells from the SO[16,23], which can be generated either in the Pacific or eastern Indian regions of the SO (Figure 1). These coasts will be the study area for this research."*

The discussion has also been modified to highlight the global nature of the problem:
L425-436: *"The findings of this study underscore the current and future importance of SO swell events along the entire Pacific coast of the Americas, as well as their variability. Furthermore, it anticipates that this hazard also poses similar risks in other coastal regions worldwide, affecting both continental margins and low-lying islands, where exposure to SO swells can be especially critical when combined with sea-level rise[49]. This broader perspective highlights that the conclusions drawn here are not limited to the Americas but may be relevant to other regions where SO swells contribute significantly to coastal hazards.*

*Building on this broader outlook, this study represents an initial step toward a deeper understanding of these events, addressing the analysis from a regional perspective. From this standpoint, there is a significant path forward in two key directions: first, expanding the analysis to a larger scale to identify the global extent of the affected coast; and second, conducting studies at the local scale to investigate the compound effects of these swells*

*combined with other dynamics that pose the highest risk to coastal areas, as well as modeling their resulting impacts."*

Line-by-line Comments

• First paragraph is really well written and very easy to understand.

Thank you for the comment.

• Line 39: what does "moderated at long distances" mean?

We intended to indicate that waves decrease in height after propagating over long distances, and that extremely large waves are not observed once they have traveled very far from their generation area. We have rewritten the sentence to improve its clarity.

L42-44: *"As a result of the dispersion and dissipation of swells, those reaching very distant coasts are characterized by attenuated wave heights and very long periods."*

• Lines 62 – 64: There are a bunch of publications that look at the combination of different sea level components, especially on the US West Coast. I was surprised they weren't cited here. You don't have to cite them, but could strengthen this part. They are listed below.

Serafin, K. A., Ruggiero, P., & Stockdon, H. F. (2017). The relative contribution of waves, tides, and nontidal residuals to extreme total water levels on US West Coast sandy beaches. Geophysical Research Letters, 44(4), 1839-1847.

Melet, A., Meyssignac, B., Almar, R., & Le Cozannet, G. (2018). Under-estimated wave contribution to coastal sea-level rise. Nature Climate Change, 8(3), 234-239.

Melet, A., Almar, R., Hemer, M., Le Cozannet, G., Meyssignac, B., & Ruggiero, P. (2020). Contribution of wave setup to projected coastal sea level changes. Journal of Geophysical Research: Oceans, 125(8), e2020JC016078.

Marcos, M., Rohmer, J., Vousdoukas, M. I., Mentaschi, L., Le Cozannet, G., & Amores, A. (2019). Increased extreme coastal water levels due to the combined action of storm surges and wind waves. Geophysical Research Letters, 46(8), 4356-4364.

Thank you very much for the references provided. All of them have been added to the manuscript.

L74-77: *"Different total sea level components, such as those induced by astronomical tides[32], interannual sea level oscillations (e.g., due to ENSO[33,34]), storm surges[35,36] and projected mean sea level rise[37,38], can occur in combination with large wave setup[39–42]."*

• Line 73: "This area" will be the study for the research – it's not clear what this area pertains to. The SO? The pacific or eastern Indian ocean? Please clarify.

Thank you for the comment. We were actually referring to the western coast of the American continent as the study area. We have modified the text to make this clearer, as it could be misinterpreted in the current wording.

L89-90: *"These coasts will be the study area for this research."*

• Line 105: if the wave dataset is a hindcast, it should be called that here, rather than just in the methods, so there is no mistaking it for observations.

Thank you for the comment. We have modified the sentence according to the reviewer's suggestion.

L123-124: *"Southern Ocean swells were analyzed using a 45-year (1979-2023) wave hindcast with an hourly temporal resolution."*

• ~Line 180 Seasonal variability – it might be interesting to bring in the publication below, which found off the California coast the southern hemisphere swell events were happening during daily weather types occurring in austral winter

Rueda, A., Hegermiller, C. A., Antolinez, J. A., Camus, P., Vitousek, S., Ruggiero, P., ... & Mendez, F. J. (2017). Multiscale climate emulator of multimodal wave spectra: MUSCLE-spectra. Journal of Geophysical Research: Oceans, 122(2), 1400-1415.

Thank you for the suggestion. We have included the reference in the section on seasonal variability.

L207-208: *"This pattern is consistent with previous studies showing the arrival of SO swells at the California coast during the austral winter[58]."*

• Lines 211-213 – it doesn't seem that correlation values were below 0.4 at all locations. From Figure SM6, it actually seems like values are higher than 0.4 in points 9, 11, 13, and 15 – 18.

Thank you for the comment. Following a previous remark, we have updated the figure (now Figure 3) with the newly calculated correlations and rewritten the text accordingly to reflect the new values.

L234-238: *"In contrast, the correlations with ENSO are weaker. Correlations with the negative phase of this climatic mode (the Niño phase) were identified, but the correlation values remained below 0.6 across all analyzed locations (Figure 3), with 78% of them being statistically significant at the 90% confidence level. The most robust results can be observed along the coasts from Peru to Mexico, with values ranging between 0.4 and 0.6."*

• Lines 274 and 279 – why only flooding is accounted for? What about the potential for coastal erosion? Wave setup is generally more related to flooding, but runup has been associated strongly with erosion.

Thank you for the insightful comment. We agree that the potential for coastal erosion should also be acknowledged, as wave runup has been strongly associated with erosion processes. We have revised the text to clarify that, in addition to flooding, large SO swells may also contribute to coastal erosion.

L302-305: *"The combination of relatively high wave heights and long periods can make these swells hazardous, as their long periods enhance shoaling and run-up while their typically narrow spectra promote the generation of infragravity waves. Therefore, these events can drive significant water level increases at the coast, leading to coastal flooding and severe erosion (Table 1)."*

L308-311: *"Moreover, while this study does not delve into the interactions of these events with other dynamics, it is likely that wave-induced surges may combine with other phenomena, such as sea level anomalies driven by atmospheric conditions, ENSO-related sea level variations, and astronomical tides, to trigger extreme flood and erosion events."*

• Line 303: increasing exposure to what?

Thank you for the observation. We have revised the sentence to clarify that the increased exposure refers to coastal populations and infrastructure to wave impacts.

L332-335: *"Potentially hazardous SO swells are more likely to occur during austral winter, which coincides with boreal summer, which is the peak season for coastal recreational use in the NH, increasing exposure of coastal populations and infrastructure to wave impacts."*

• Paragraph from lines 318 – 324: are there any studies in the literature that show the compounding effects of SLR + increases in swells + SAM? The interaction of more than the two dynamics mentioned, but there are potentially more processes involved in this interaction, so it would be beneficial to the manuscript to acknowledge this.

Thank you for the comment. This paragraph is intended to address long-term trends, not short-term variability. Among the interacting components, sea level rise is by far the most robust trend, which is why we highlighted it explicitly. Our aim was not to imply that the interaction occurs exclusively with SLR, but rather to emphasize that the increasing severity of SO swells coincides with a well-established positive trend in sea level rise, for which we have now incorporated additional references. We also note that the interaction of SO swells with other dynamics (e.g., ENSO-related anomalies, atmospheric variability, tides) was already discussed earlier in the manuscript, at the beginning of the Discussion section. We did not find robust evidence in the literature supporting long-term changes in ENSO that would justify including it here. With respect to the SAM, however, robust trends have been reported, and we have incorporated this aspect in the text as a possible explanation for the observed changes in SO swells.

L308-311: *"Moreover, while this study does not delve into the interactions of these events with other dynamics, it is likely that wave-induced surges may combine with other phenomena, such as sea level anomalies driven by atmospheric conditions, ENSO-related sea level variations, and astronomical tides, to trigger extreme flood and erosion events."*

L362-364: *"These trends are consistent with increases in wind speed in the SO over recent decades[69,76], possible increases in storm activity[71], and a positive trend in the SAM[77]."*

*L372-379: "Although long-term trends represent, in relative terms, the timescale that has contributed the smallest variations in wave power compared with the mean value during the study period, its implications for coastal impacts remain significant. The presence of robust long-term trends indicates a growing severity of SO events over recent decades, which is likely to intensify further over time. Moreover, the increasing severity of these swells is compounded by rising mean sea levels[37,38]. As a result, extreme coastal water levels associated with these swells are likely to exhibit an accelerating positive trend due to the interaction of these two dynamics, thereby increasing the hazard for coastal communities and infrastructure."*

• Lines 417 to 419: results for trends are based on individual partitions, right? Not on total integrated wave parameters? It's not clear from the writing

Thank you for the comment. We agree that the original wording could lead to ambiguity. In the revised text, we now make explicit that all analyses specifically concerning SO swells are based on partitioned integrated wave parameters, while total integrated parameters are used only when explicitly indicated to assess the contribution of SO swells to the overall wave climate. This clarification complements the earlier explanation in the *Methods* section, where we describe the distinction between total and partitioned parameters.

L483-487: *"Throughout the article, total integrated wave parameters refer to values derived from the full wave spectrum, whereas partitioned integrated wave parameters refer to those obtained from individual partitions. All analyses that specifically concern SO swells were based on partitioned integrated wave parameters, whereas total integrated parameters were used only when explicitly stated to assess the relative importance of SO swells within the overall wave climate."*

• The framework steps listed from 455 to 485 are somewhat difficult to follow, especially from steps 1 to 4. If some of these steps can be shown with examples in Figure SM2, that would be helpful.

Thank you for the comment. We have generated a figure with four panels, one for each step from 1 to 4, and added it to the Supplementary Material.

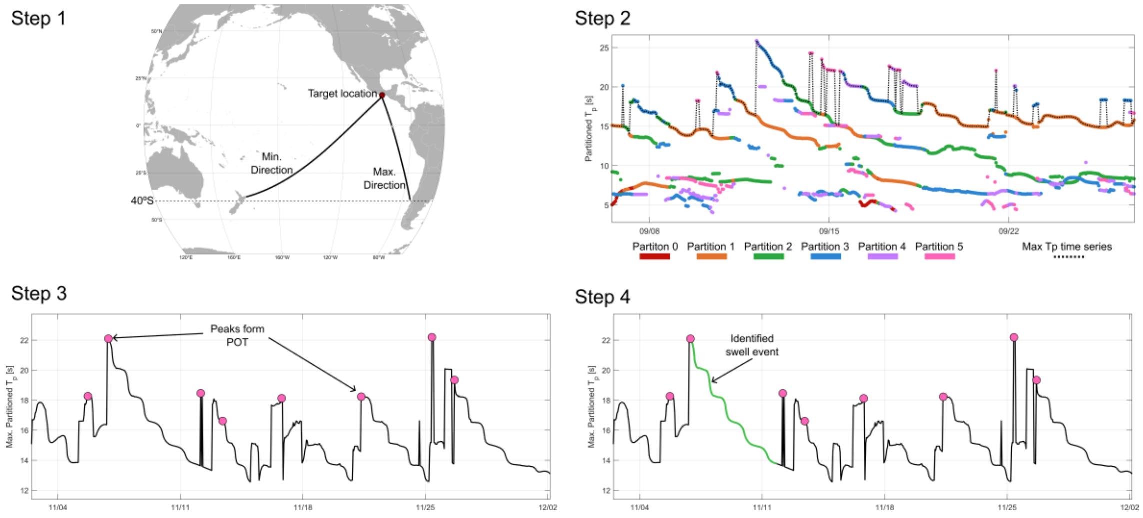


***Figure SM1**: Methodology for swell identification. Each panel illustrates one step of the procedure, from Step 1 to Step 4.*

• Page 10, Step 6 – why 48h? Is this related to the duration of ET/storms?

Thank you for the comment. This choice was made to ensure that enough data points were available to obtain a robust fit of the Tp slope. Previous applications of the algorithm used a 24-hour window, but those studies relied on total integrated parameters, where overlap between events cannot occur and the continuity of the Tp slope is therefore shorter. In our case, this limitation does not apply, and we found that 48 hours provided a more reliable fit. We have clarified this explanation in the revised manuscript.

L544-547: *"<u>Event origin calculation</u>: The origin coordinates are estimated by fitting a line from the event's start to either the maximum $H_s$, minimum $T_p$, or the event's end, whichever occurs first, provided that the selected event period lasts at least 48 hours to ensure a robust fit. The origin uncertainty assessed through confidence intervals of the fitted slope."*

• Page 10, Steps 3-9 - Why 100 events? A bit confused where this number comes from.

Thank you for the comment. The choice of 100 events was a purely empirical criterion to ensure the robustness of the method; another number could have worked as well. The rationale was to use a sufficiently large sample to overcome potential difficulties of the algorithm in tracking swells. For instance, at the early stages of a swell, the algorithm may lose continuity and fail to capture the complete event. This can occur because the initial behavior of the swell is unstable, or because the partitions only capture it intermittently when its energy is very low—below partition 6—and it only becomes visible once the other partitions have less energy. In such cases, even if the swell eventually develops into a powerful system, it is only properly identified and tracked in later iterations, once the unstable initial records are discarded, as described in the Methods. In addition, some swells had to be excluded from the analysis. Considering all this, we regarded 100 as an adequate number to reliably capture the 20 most energetic events per year. We have added an explanation in the Methods clarifying the reason for selecting 100, following this discussion.

L531-536: *"<u>Peak Over Threshold (POT) method</u>: The POT method[86] is applied to the $T_p$ time series from step 2, using a threshold that results in 100 exceedance peaks. The choice of 100 events was made to ensure a sufficiently large sample and to avoid potential issues of the algorithm in reliably tracking swells, especially during the early stages of a swell, when they may not be continuously defined due to their low energy. This criterion ensures that the 20 most energetic events per year are consistently captured."*

• Line 552 – the term “climate variability” is brought up here for the first time, what does this mean? Does it include all the variabilities listed below?

Thank you for the comment. We agree that the term “climate variability” had not been properly introduced. We have now defined it in the Introduction section.

L117-119: *“Their main properties, such as wave height, period, and direction, are examined. Additionally, this study focuses on providing a comprehensive understanding of their climate variability across different time scales, with particular emphasis on long-term trends.”*

• Lines 554-555: has seasonal variability been assessed for each season separately OR the maximum relates to winter and the minimum to summer? This is unclear.

Thank you for the comment. We considered all SO swells throughout the study period falling within each season, calculated the seasonal means, and then defined seasonality as the difference between the maximum and minimum seasonal values. We did not restrict the analysis to winter and summer only. We have rewritten this part of the Methods to improve clarity.

L646-648: *“Seasonal variability was computed as the mean of the relative difference between the maximum and minimum seasonal means* $P_{wp}$ *for the study period across the four seasons, normalized by the study period’s mean value.”*

• Figure SM2: where are the partitions T0, T1,…T6 in this figure? Is each dot a partition? Or each partitions have its own time series in this plot with a sequence of dots? It might be helpful to highlight how the partitions are represented in these plots so that the readers can better understand the explanation of the algorithm steps and visualize the independence of swell events. Maybe highlight which is the corresponding Tp and direction to the peak Hs used to derive all stats from the analyses.

Thank you for the comment. In Figure SM2 all partitions are mixed, and the gray dots may correspond to any of the six considered. Since the relative energy between wave systems varies at each time step, a given partition (e.g., partition 1) does not always represent the same wave system. For example, at the beginning of its lifetime a wave system may be represented by partition 3 because its energy is low compared with the total, later shift to partition 1 as it becomes dominant, and then change again. This makes the figure very

chaotic and difficult to interpret if all partitions are displayed simultaneously in different colors, which is why we chose to plot everything in gray. However, we have incorporated into the figure caption a more detailed explanation of what the gray dots represent. This has also been illustrated in the methodology figure (Figure SM14; see comments above). In addition, we have added to the figure the peak Hs of the event together with the associated Tp and Dirp.

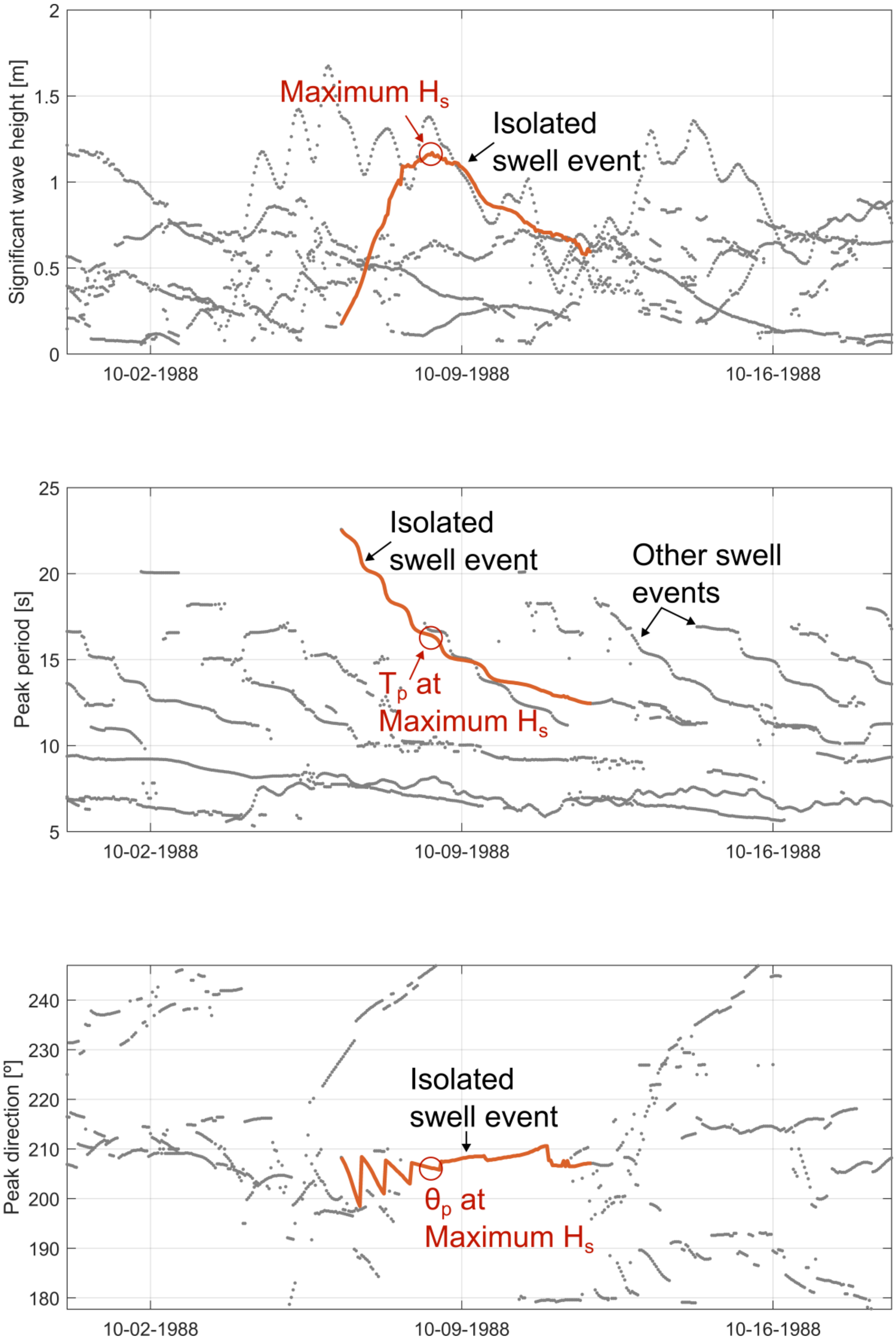

Significant wave height [m]
Maximum $H_s$
Isolated swell event
10-02-1988
10-09-1988
10-16-1988
Peak period [s]
Isolated swell event
Other swell events
$T_p$ at Maximum $H_s$
Peak direction [°]
Isolated swell event
$\theta_p$ at Maximum $H_s$

*Figure SM2: Isolated swell event within the time series of spectral partitions. Gray dots represent the combined partitioned data from the six partitions considered in the study. The event is highlighted in orange. The top panel shows the significant wave height, the middle panel shows the peak period, and the bottom panel displays the peak direction. The maximum significant wave height of the event is highlighted in red, along with the associated peak period and peak direction.*

• Figure SM4: it is very challenging to visualize the gray arrows, they should be thicker and perhaps represented by a different color. Another suggestion is to consider using dashed gray lines, this would be interesting because they would overlap with black arrows where the direction does not change as much and would highlight even more the different toward the north.

Thank you for the suggestion. We have applied the suggested changes and regenerated the figure. We believe it can now be appreciated much better.

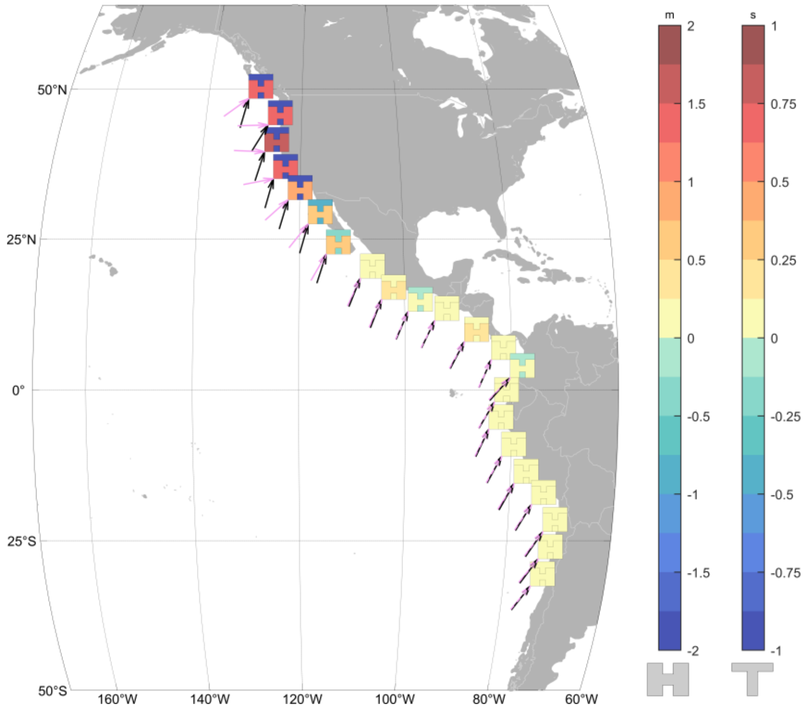


***Figure SM3**: Differences in the mean significant wave height (H within the square), peak period (T within the square), and peak direction (arrows) of the SO swell events compared to the overall conditions at the time of the arrival of the events. Pink arrows indicate the directions associated with total wave conditions; black arrows indicate the directions associated with SO swells.*

Reviewer #3 (Remarks to the Author):

Major comments:

The analyses and findings presented in this study are good and well worth reporting to the scientific community. The authors present a robust methodology for studying Southern Ocean generated swells that reach the west coast of the American continent, with a focus on the 20 most intense events per year (one every ~2.5 weeks on average). While not entirely novel, there are interesting (and potentially important) results regarding seasonal variability and influence of climate oscillations (ENSO and SAM), alongside an assessment of long-term trends. The separation of the swell wave extremes' variance into seasonal, interannual and long-term trends, utilising swell identification and tracking, is an excellent contribution.

We sincerely thank Reviewer #3 for the constructive and insightful comments. We appreciate the positive evaluation of the manuscript and the helpful suggestions that have contributed to improving its clarity, rigor, and overall quality. In the revised version, we have carefully addressed the major and minor points raised, clarifying definitions, expanding methodological explanations, and refining the discussion where needed. These revisions have strengthened the robustness and readability of the manuscript, and we are grateful to the reviewer for the thoughtful feedback that has been instrumental in enhancing this work.

• However, in my opinion, the manuscript suffers from several significant flaws which need to be ameliorated before publication. Considering its focus on coastal damage and concomitant risk associated with swell events, the assertion that "Specifically, the Pacific coast of the Americas is one of the coastal regions in the world most affected by these events" (lines 70-71) needs to be better unpacked and put into context. For instance, over the last decade, an increasing number of journal articles have identified and discussed the impacts of distant-source swell events (with Southern Ocean sources in many cases), modulated by tides and sea level, to Pacific and Indian Ocean Island nations. Only one of the oldest of these papers (Hoeke et al. 2013) is cited but not really discussed. A very non-comprehensive list of more recent publications includes:

Wadey, et al. (2017). Coastal flooding in the Maldives: an assessment of historic events and their implications. Nat. Hazards 89, 131–159. doi: 10.1007/s11069-017-2957-5

Ford, et al. (2018). Inundation of a low-lying Urban atoll island: Majuro, Marshall Islands. Nat. Hazards 91, 1273–1297. doi: 10.1007/s11069-018-3183-5.

Canavesio, R. (2019). Distant swells and their impacts on atolls and tropical coastlines. The example of submersions produced by lagoon water filling and flushing currents in French Polynesia during 1996 and 2011 mega swells. Global and Planetary Change, 177, 116-126.

Wandres, et al. (2020). Distant-source swells cause coastal inundation on Fiji's coral coast. Front. Mar. Sci. 7:546. doi: 10.3389/fmars.2020.00546.

Andréfouët, et al. (2023). Hydrodynamical impact of the July 2022 'Code Red'distant mega-swell on Apataki Atoll, Tuamotu Archipelago. Global and Planetary Change, 228, 104194.

Wandres, et al. (2023). Wave climate variability and trends in Tuvalu based on a 44-year high-resolution wave hindcast. Journal of Geophysical Research: Oceans, 128(4), e2022JC019523.

As outlined in Storlazzi, et al. (2018) and other papers, such events represent an increasingly existential threat (when coupled with sea level rise) to many such oceanic communities. This manuscript should therefore compare and contrast the Pacific coast of the Americas with other areas of the world at risk of such swell-driven coastal impacts as reported in these and other studies, including (where applicable) other studies' findings on Southern Ocean swell extreme event trends and variability. For instance, a single event may result in damages/loss equivalent to a substantial portion of annual GDP at many oceanic island locations, which undoubtably suffer the highest relative impacts and future risk; the "Pacific coast of the Americas", on the other hand, likely suffer significantly higher overall gross level of impacts in terms of total economic costs (owing to their much larger size and populations). This is just an example - to reiterate my overall view - particularly given the general audience of Nature Communications - a better framing of the context of the findings of this study against other climate-related hazards studies, especially ones with similar motivations/underlying coastal hazard processes, is required.

Storlazzi, Curt D., et al. "Most atolls will be uninhabitable by the mid-21st century because of sea-level rise exacerbating wave-driven flooding." Science advances 4.4 (2018): eaap9741.

We thank the reviewer for this insightful comment. We fully agree with the points raised in the discussion. We believe that incorporating the global nature of the problem, rather than restricting the focus to a regional scale such as the Pacific coast of the Americas, greatly enhances the reach of the article. We are also grateful for the references provided, all of which have been incorporated into the revised manuscript. Following the reviewer's advice, and always keeping in mind the space limitations of this type of journal, we have added a new paragraph highlighting the global scope of the problem. Furthermore, we

now explicitly distinguish between the effects observed in low-lying islands and atolls, and those occurring along continental margins, as kindly suggested by the reviewer.

Below we include the modification introduced in the revised version:

L23-30: "*A remarkable ocean wave event in early May 2015 impacted a large portion of the Pacific South and Central American coasts. Its consequences were devastating, not only because of its intrinsic energy content but also due to its interaction with large spring tidal amplitudes, leading to severe flooding. This combination resulted in extensive erosion; damage to property, infrastructure and ecosystems; and human loss of life, with dozens of fatalities reported[1,2]. This event was not an isolated occurrence. Similarly, in May 2007, a highly energetic wave event generated south of South Africa propagated more than 5,000 km across the Indian Ocean, flooding 88 islands across 18 atolls of the Maldives. The episode displaced more than 1,600 people, damaged over 500 houses, and disrupted critical infrastructure[3]. "*

L72-90: *"Owing to the considerable amount of energy carried by swell events that propagate away from the SO, when they reach land, they can cause considerable damage to the coast (e.g., erosion[26–29]) or substantial water level elevations induced by wave setup[3,30,31], which can induce coastal flooding. Different total sea level components, such as those induced by astronomical tides[32], interannual sea level oscillations (e.g., due to ENSO[33,34]), storm surges[35,36] and projected mean sea level rise[37,38], can occur in combination with large wave setup[39–42]. Southern Ocean swells can affect both insular and continental coastlines across all ocean basins. They propagate thousands of kilometers to reach distant shores in the Pacific, Indian, and Atlantic Oceans, with the capacity to generate impacts in locations as diverse as low-lying atolls or continental margins. In island nations, a single event can produce widespread flooding[43,44], lagoon submersions[45,46], ecosystem damage[45,47], and economic losses equivalent to a large fraction of the annual Gross Domestic Product (GDP)[44,48], sometimes accompanied by community displacement and severe disruptions to livelihoods[43,48]. The existential nature of these impacts is expected to worsen with rising sea levels, threatening the long-term habitability of atoll islands[49]. In contrast, in continental margins impacts are generally not existential but translate into recurrent coastal erosion[2,28,29], flooding of communities[30,31], casualties, damages to infrastructure and disruption of maritime activities[1]. In particular, the Pacific coast of the Americas is one of the continental coastal regions in the world most affected by these events[1,50]. The western coasts of the American continents are fully exposed to energetic swells from the SO[16,23], which can be generated either in the Pacific or eastern Indian regions of the SO (Figure 1). These coasts will be the study area for this research."*

The discussion has also been modified to highlight the global nature of the problem: L425-436: *"The findings of this study underscore the current and future importance of SO swell events along the entire Pacific coast of the Americas, as well as their variability. Furthermore, it anticipates that this hazard also poses similar risks in other coastal regions worldwide, affecting both continental margins and low-lying islands, where exposure to SO swells can be especially critical when combined with sea-level rise[49]. This broader perspective highlights that the conclusions drawn here are not limited to the Americas but may be relevant to other regions where SO swells contribute significantly to coastal hazards.*

*Building on this broader outlook, this study represents an initial step toward a deeper understanding of these events, addressing the analysis from a regional perspective. From this standpoint, there is a significant path forward in two key directions: first, expanding the analysis to a larger scale to identify the global extent of the affected coast; and second, conducting studies at the local scale to investigate the compound effects of these swells combined with other dynamics that pose the highest risk to coastal areas, as well as modeling their resulting impacts."*

Other areas that need to be improved:

• The reported research is (apparently) based on a (new) global wave hindcast developed specifically for this analysis? Building a global implementation of WAVEWATCH III, with tuning and verification, is not a trivial exercise … why weren't existing published, well verified wave hindcasts such as the CAWCR (Durrant et al. 2014/ Smith et al. 2021) or GOW2 (Perez et al. 2017) used? As far as I know, these existing wave hindcasts (and undoubtably others) make available the same spectral partitioning variables used in this study. They also offer extensive global verification reports from satellite and from buoys, enabling understanding of strengths and weaknesses of these extant hindcasts to different wave conditions. Verification of the wave hindcast presented here is (apparently) limited to significant wave height (and not other bulk or partitioned wave parameters) at five coastal wave buoys offshore of central and southern California (Figure SM3/Table SM2), thus representing only a small portion of the study region, near its northernmost limit, raising questions about the hindcasts' overall veracity. Both better justification of why extant, published wave hindcasts were not used and much better verification/assurances of the wave hindcast that was used is needed.

We thank the reviewer for this valuable comment. We fully agree that a thorough validation of the hindcast is essential to provide robustness to the analysis. The hindcast employed in this study is indeed a new global product developed at IHCantabria, forced

with ERA5. This product has been extensively calibrated against satellite data following the methodology of Stopa (2018), and subsequently validated using a global database of buoys and altimetry. A dedicated article describing the full validation is currently in preparation. To ensure transparency in this work, we have included in the Supplementary Material: (i) a detailed description of the model configuration, (ii) the calibration procedure, and (iii) the validation results, with a focus on the Pacific Ocean. Validation results show small biases, low RMSE, and high correlations, comparable to or improving upon those reported in existing hindcasts, thus supporting the reliability of this product.

The reason for using this product rather than GOW2 is primarily the availability of wave partitions (1 sea + 5 swells), information not provided by GOW2, which only distributes full spectra but not the partitioned parameters required in this study. The reason for not using CAWCR or other products is that by developing our own hindcast we ensure full control over the data, configuration, and validation.

Beyond the global validation, we also included a specific validation of Southern Ocean swells. This process is inherently complex, as it requires spectral data of sufficient length and quality. We explored both the NOAA and CDIP databases, and selected CDIP spectra as they provided higher quality for this analysis. We made extensive attempts to include NOAA buoys in the validation, since a few of them provide data along other parts of the American coast. However, the results were very inconclusive, most likely due to issues related to the spectral reconstruction of these buoys. At first, one might attribute these discrepancies to model errors, but this was ruled out after confirming that NOAA buoys located near CDIP stations, which otherwise yielded very good results, still showed noisy and inconclusive comparisons. For this reason, we ultimately relied on CDIP spectra.

Among these, we selected five buoys with sufficiently long records in affected regions, although most were clustered in a single area. No buoys in the Southern Hemisphere outside of generation zones met the required standards. Moreover, the swell identification algorithm requires clean and continuous spectral data, a condition often not met by buoy spectra. Therefore, applying the algorithm directly to buoy-derived partitions did not yield interpretable results. Instead, we adopted an alternative validation approach based on equivalent significant wave heights reconstructed from the directional and frequency sectors associated with Southern Ocean swells at the selected buoys. This approach, while indirect, provides meaningful validation of the hindcast skill in capturing SO swell signals at the coast, especially considering the complexity of the comparison.

• Regarding the wave partition tracking algorithm (“Isolation of swells”) upon which this study relies, there appears to be the assertion that the “algorithm has been previously

applied using total integrated wave parameters". However, there is a nearly 30-year history development of space-time tracking of wave spectral partition algorithms: see Delpey, et al. (2010) and citations therein for a good review of its history and use up to that point, including a more complex adaptation of the swell travel distance equation than the Eq. 1 used in this paper. While the partition tracking algorithms used here may be different and improved from previous, it is not clear what makes them "novel" as stated in the abstract and discussion. More discussion and justification in this regard is needed.

Delpey, M. T., Ardhuin, F., Collard, F., & Chapron, B. (2010). Space-time structure of long ocean swell fields. Journal of Geophysical Research: Oceans, 115(C12).

We thank the reviewer for raising this point and for drawing our attention to the long history of space–time tracking of spectral partitions (e.g., Delpey et al., 2010, and references therein). We agree that acknowledging this background strengthens the manuscript, and we have revised the text accordingly. Our algorithm builds on the spectral partitioning framework of Portilla et al. (2009), which is already cited in the manuscript, and introduces a novel aspect by adapting it to the identification of swell events from spectral partition time series at specific coastal locations. In contrast to studies such as Delpey et al. (2010), which reconstruct basin-scale swell fields using spatially continuous spectral data, our methodology operates directly on partitioned time series at a single location. Specifically, we (i) filter partitions to ensure a Southern Ocean origin, (ii) construct a Tp-based series, (iii) identify peaks with a POT method, and (iv) connect successive points using a weighted Euclidean distance in Tp–Hs–time space under strict continuity constraints (ΔTp, ΔHs, Δθ). This procedure enables the construction of a homogeneous catalogue of energetic swell events at the coast over 45 years. We would also like to clarify that our initial use of the term "novel methodology" was not intended to overlook previous developments, but rather to emphasize this adaptation of Portilla's framework to coastal time series of spectral partitions. Following the reviewer's suggestion, we have softened this wording in the revised manuscript, now referring to it as an "adapted methodology," while explicitly citing Delpey et al. (2010) to situate our work within the broader development of swell tracking techniques.

We have modified the introduction:

L115-117: *"Therefore, to analyze these swell events in detail, a methodology based on spectral data is proposed and applied, enabling, for the first time, their individual identification and characterization along the entire coast of the Americas."*

Discussion:

L422-424: *"This approach has enabled a detailed and individualized study of large SO swells for the first time, which would have been difficult to achieve using more traditional approaches on the basis of total integrated parameters."*

Methods:

L507-509: *"The approach for isolating swells is based on previous works [53–55]. Unlike space–time tracking methods developed to reconstruct basin-scale swell fields [85], it is applied here to identify and characterize swell events directly from time series at individual coastal locations."*

Technical aspects that could be improved/better discussed:

• The correlations between swell mean wave power and the SAM and ENSO indices are presented, but the authors do not indicate whether these correlations are statistically significant at the 95% confidence level. That might be because they compare annual values, what would happen if a higher temporal resolution were used?

Thank you for the comment. We have analyzed the statistical significance of the correlations and included this information in both the figures and the results description.

L224-240: *"The results indicate a strong correlation between the annual mean wave power of the selected events and the positive phase of the SAM, with all analyzed locations showing significant correlations at the 90% confidence level (Figure 3). During this phase, westerly winds in the Southern Hemisphere (SH) intensify and shift poleward, along with extratropical cyclones generated in this region[60,61], which likely contribute to increased wave power in events generated south of 40°S. These findings align with previous studies showing a strong correlation between the positive phase of this index and both mean and extreme wave heights in the SO and eastern Pacific Ocean[62–65]. The observed correlations along the Pacific coast of the Americas are particularly relevant north of the equator, consistently exceeding 0.5. The maximum values are found along the coast of Guatemala and the Southernmost coast of Mexico, ranging from 0.6 to 0.7. South of 5°S, the correlation values fall below 0.4 at most locations.*

*In contrast, the correlations with ENSO are weaker. Correlations with the negative phase of this climatic mode (the Niño phase) were identified, but the correlation values remained below 0.6 across all analyzed locations (Figure 3), with 78% of them being statistically significant at the 90% confidence level. The most robust results can be observed along the coasts from Peru to Mexico, with values ranging between 0.4 and 0.6. These findings align with previous research[63,64] indicating a correlation between the negative phase of ENSO*

*and the positive phase of the SAM, leading to increased wave heights in the southeastern Pacific during negative ENSO phases."*

The reason for using annual values is that the study is based on a sample of 20 individual events per year. As discussed in the manuscript, this selection and aggregation were designed to obtain robust metrics that compensate for the noise associated with spectral partitioning and the complexity of the swell identification. Given this framework, and the fact that individual events do not provide information for every month (with some months repeated and others missing), we decided to work with annual values.

L619-625: *"The correlation between changes in wave power and different teleconnection patterns was calculated using linear regression models (Eq. 4), which in the univariate case are mathematically equivalent to Pearson's linear correlation[87]. Annual indices were used to explore interannual variability through aggregated annual metrics. For SAM, we used the annual observation-based Marshall index[59]. For ENSO, we obtained annual values from the monthly MEI index using an enso-year definition (May–April)[88–91], ensuring that the full evolution of individual ENSO episodes (El Niño/La Niña) is contained within a single year, while keeping the main wave season (JJA) intact and not split across two different years."*

• The authors do show statistical significance in the trends, but only at the 90% confidence level. What would happen if the 95% confidence level is chosen instead? Are the trends statistically significant then?

Thank you for the comment. We adopted a 90% confidence level for trend detection to remain consistent with much of the climate literature and with IPCC reporting conventions. Given the inherent variability and noise in climate data, a 90% threshold is commonly used to assess whether trends are robust. Following other reviewers' suggestions, we also revised our trend methodology to use Sen's slope for magnitudes and the Mann–Kendall test for significance—both non-parametric and widely used approaches that reduce sensitivity to outliers and distributional assumptions, thereby improving robustness.

Regarding the sensitivity to the confidence level, increasing the threshold to 95% reduces the percentage of significant grid points as follows: Hs from 77% → 64%, Pw from 82% → 73%, and event frequency from 68% → 59%. Even at 95%, the proportion of significant points remains ≥ ~60% across all variables, supporting the robustness of the results. We have added this explanation and the methodological details in the revised manuscript.

L633-641: *"Trends in the different indicators were estimated using the non-parametric Sen's slope estimator to minimize the influence of outliers on the results. The statistical*

*significance of the trend was assessed with the Mann-Kendall trend test (MK)* [92,93]*, which evaluates the presence of a monotonic upward or downward trend over time by testing if the slope of the estimated regression line differs from zero. The Sen–MK combination has been widely applied in the climate science literature, as it provides a robust, distribution-free framework for detecting and quantifying monotonic trends in environmental and climatic time series*[70,94,95] *where the assumptions of parametric methods are often not met. A trend was considered statistically robust when MK confirmed a significance level below 0.1, a threshold commonly used in climate studies*[94,96,97] *and consistent with conventions adopted in IPCC assessments*[98]*."*

• Trend magnitude vs. model uncertainty: the reported trend in wave height (~0.3 cm/year, yielding ~13.5 cm over 45 years) is very close to the model's root-mean-squared error (~11 cm, from Figure SM3). Is there scope to discuss this?

We thank the reviewer for this thoughtful comment. We report that the long-term trends in significant wave height are relatively small ~0.3 cm $yr^{-1}$ (~13.5 cm over 45 years). Regarding the comparison with model errors, we would like to stress that the validation was purposely designed to be more robust than applying the swell-identification algorithm directly to buoy records, given the noisy nature of spectral observations. For this reason, we used an equivalent Hs associated with Southern Ocean energy, which can in some cases be slightly different from the isolated swell events. This approach provides a stable and reliable validation metric that fulfills its purpose of giving confidence in the hindcast. However, it is not intended for a direct comparison with the magnitude of the reported long-term trends, which are based on event-scale metrics over 45 years and across the full study domain. The trends we report are statistically significant and spatially coherent, which supports their robustness.

• The manuscript does not have an in-depth discussion on the reason for the observed trends. Can they be linked to or compared against, for example, the results presented in Young and Ribal (2019), or the trends in the Southern Annular Mode during the historical hindcast?

Young, I. R., & Ribal, A. (2019). Multiplatform evaluation of global trends in wind speed and wave height. Science, 364(6440), 548-552.

We thank the reviewer for the comment. We have added a discussion comparing our results with previous studies based on historical data, and also analyzing their relationship with projection-based studies that provide consistent findings:

L347-364: *"However, outside the generation regions, studies relying solely on integrated parameters may mask the signals of SO swells*[67]*. This occurs because the change signal in the integrated parameter also combines the contributions of all individual systems. Consequently, along much of the studied coastline, where these swells are often not the dominant system, their change signal is not captured by such studies, making it difficult to assess their past or future behavior. This study takes a step farther by examining the historical trend of these swells outside their generation zones. A statistically robust positive historical trend along nearly the entire coast of the Americas has been observed, affecting several indicators, including wave height, period and power. Considering the origin of these swells, our results are consistent with previous studies based on altimetry*[68,69] *and hindcast*[70,71] *data, which reported increases in mean and high-percentile $H_s$ in the SO. Moreover, the agreement in the sign of change with projected changes in $H_s$ reported in earlier works*[72–75] *in this ocean region suggest that a continued increase in the future is likely. Most importantly, these findings are also in line with prior conclusions drawn from wave spectra in climate projections*[24,67]*, which showed a robust increase in SO swell energy along all points where these swells propagate including the west coast of the Americas by the end of the century. These trends are consistent with increases in wind speed in the SO over recent decades*[69,76]*, possible increases in storm activity*[71]*, and a positive trend in the SAM*[77]*."*

Minor notes:

- Line 14: "characterizing their primary characteristics" might need a rephrasing.

Thank you for the comment. We have rephrased the sentence.

- Line 25: "For that matter…" consider a different turn of phrase. It doesn't really make sense.

Thank you for the comment. We have replaced this connector with a more appropriate phrasing.

- "destructive" … "damage" … this is an oversimplification. Wave power (the ability to do work) is roughly linearly related to breaking wave height, wave setup and wave overtopping, etc… and likely more correlated with erosion, although that is more complex and depends on many other factors.

Thank you for the comment. We agree that the original phrasing was an oversimplification. We have revised the paragraph.

L44-53: *"Note that the power (energy flux) of waves is a function of the wave height and the wave period to the first and second orders, respectively[6]. Hence, despite wave heights being moderated at long distances, the combination with large periods allows a significant amount of energy to reach the coastline. Additionally, waves with longer periods experience more shoaling than shorter waves transporting a similar amount of energy, resulting in a higher wave height at breaking and a larger release of energy upon dissipation. Moreover, distant long mature swells usually exhibit narrower spectra, which enhances the generation of infragravity waves. The height of these infragravity waves, released through the bound-wave mechanism, and for which a correlation with the product of the wave height and the squared period of the generating waves has been found[12,13], may also be relevant and capable of damaging coastal zones[14]."*

- Introduction, first paragraph really could/should be better written, also recommend reorganising the Introduction – e.g. lead with the discussion of May 2015 and link it with other event-driven damaging/inundation event recorded in earlier research, as suggested above.

Thank you for the comment. We have revised the Introduction accordingly: it now begins by discussing relevant damaging events, including the May 2015 episode. The original first paragraph has been moved to the third position and modified. We would also like to note that, while some explanations of wave-related technical concepts may appear simplified from an expert perspective, this was an intentional choice to ensure that the discussion remains accessible to the journal's multidisciplinary audience.

- Lines 34-35: "Swells [..] follow great circles paths, with minor deviations due to ocean currents". Ocean currents can have a massive impact on ocean swells, please see Gallet and Young (2014).

Gallet, Basile, and William R. Young. 2014. "Refraction of swell by surface currents." Journal of Marine Research 72, (2).

Thank you for the comment. The phrasing in the original manuscript followed Ardhuin et al. (2009), which described swell propagation as geodesics with minor deviations due to currents. We agree, however, that more recent studies, such as Gallet and Young (2014), have shown that surface currents can, under certain conditions, have a significant impact

on swell refraction. We have revised the sentence and added this reference to reflect both perspectives.

L41-42: *"Swells can travel thousands of kilometers across entire ocean basins, following great circle paths[7,8], with deviations due to ocean currents and marginal energy dissipation[9–11]."*

References:

- Ardhuin, F., Chapron, B., & Collard, F. (2009). Observation of swell dissipation across oceans. Geophysical Research Letters, 36(6).

- More rigorous definition of Southern Ocean is needed, i.e. "we define it as south of the *** parallel"

Thank you for the comment. We have clarified the definition of the Southern Ocean in the revised manuscript by explicitly setting its northern boundary at 40°S.

L55-57: *"The SO is the southernmost body of water; it surrounds Antarctica and connects the three main ocean basins. For practical reasons, in the present study, we consider the southernmost parts of the Atlantic, Pacific, and Indian Oceans as part of the SO, defining its limit at 40°S latitude[4]."*

- Lines 40-42: "Additionally, the height of the infragravity wave induced by long mature swells, which has been found to be correlated with the product between the wave height and the squared period of the generating waves7,8" … this statement, and the articles referenced, refer to the bound wave release mechanism of coastal infragravity wave generation. What about the breakpoint mechanism (which has a different, less consistent relationship with wave period)? Consider whether this needs to be re-phrased to include this mechanism or omitted.

Thank you for the comment. The statement and references indeed refer to the bound-wave release mechanism, and we have clarified this in the revised text. We chose not to expand further into other mechanisms at this stage, as the purpose of the introduction is to provide a broad and accessible description of the problem for a multidisciplinary readership, in line with the scope of the journal. Some simplifications are therefore intentional to maintain readability.

L49-53: *"Moreover, distant long mature swells usually exhibit narrower spectra, which enhances the generation of infragravity waves. The height of these infragravity waves,*

*released through the bound-wave mechanism, and for which a correlation with the product of the wave height and the squared period of the generating waves has been found[12,13], may also be relevant and capable of damaging coastal zones[14].”*

- Figure 1: How were the mean wave energy gain and losses calculated? Provide an explanation for the observed patterns of gains and losses of wave energy.

Thank you for the comment. The gains and losses were calculated following the approach described in Pérez et al. (2014). In a polar grid, the gain or loss of energy in each cell is computed as the difference between the incoming and outgoing flux along the great circle. The reference provided below contains the full details of the methodology. We have also added further explanation in the manuscript to facilitate understanding.

L65-68: *“The colors in the figure represent the mean gain (brown) and loss (blue) of the wave energy propagating away from any point in the SO to the target location from 1979 to 2023, calculated as the difference between the incoming and outgoing energy flux along the great circle[23].”*

References:

Pérez, J., Méndez, F. J., Menéndez, M., & Losada, I. J. (2014). ESTELA: a method for evaluating the source and travel time of the wave energy reaching a local area. Ocean Dynamics, 64(8), 1181-1191.

- Lines 136-138: why is this approach taken (i.e. to characterize the peak of the event as “corresponding to the peak spectral energy density of the event, i.e., when $Hs$ is at its maximum.”), when in the introduction section, the importance of wave power (energy flux) is discussed and is a stated integrated wave parameter used?

Thank you for the comment. In the analysis we considered both options. We ultimately chose to use Hs because wave power could only be estimated indirectly, given the lack of mean or energy period data. Although wave power would be the most direct measure of the energy carried by waves, we preferred to use a directly observed parameter for the analysis, and Hs provides that robustness.

- Figures 3 and SM6: It might be worth marking the points that show a statistically significant correlation.

Thank you for the comment. We have incorporated this change into the figure.

- Figure 3: Why not putting the left panel of Figure 3 to the right of Figure 2, and combine the right panel of Figure 3 (correlations with the Antarctic Oscillation Index) with Figure SM6 (correlations with the Multivariate ENSO Index)?

Thank you for the suggestion. We have implemented these changes and modified Figures 2 and 3 accordingly.

- Line 126: Is there any sensitivity to selecting more events per year?

Thank you for the comment. The choice of 20 events per year was made to obtain a sufficiently large sample to work with aggregated metrics that could compensate for the uncertainty associated with the complexity of identifying Southern Ocean swells from spectral partitions. We considered using only the single most energetic event, as well as samples of 5, 10, 20, or 50 events. Using only one event resulted in excessive noise, making the results difficult to interpret. As the sample size increased, the noise decreased and the robustness of the results improved. The outcomes for 10 and 20 events were very similar, although 20 provided slightly more stability. A sample of 50 was discarded because it departs too far from the extreme tail of the distribution.

As an example, results for trends and correlations with the SAM are shown using 10, 20, and 50 annual events. The outcomes display consistency and stability regardless of the number of events selected per year.

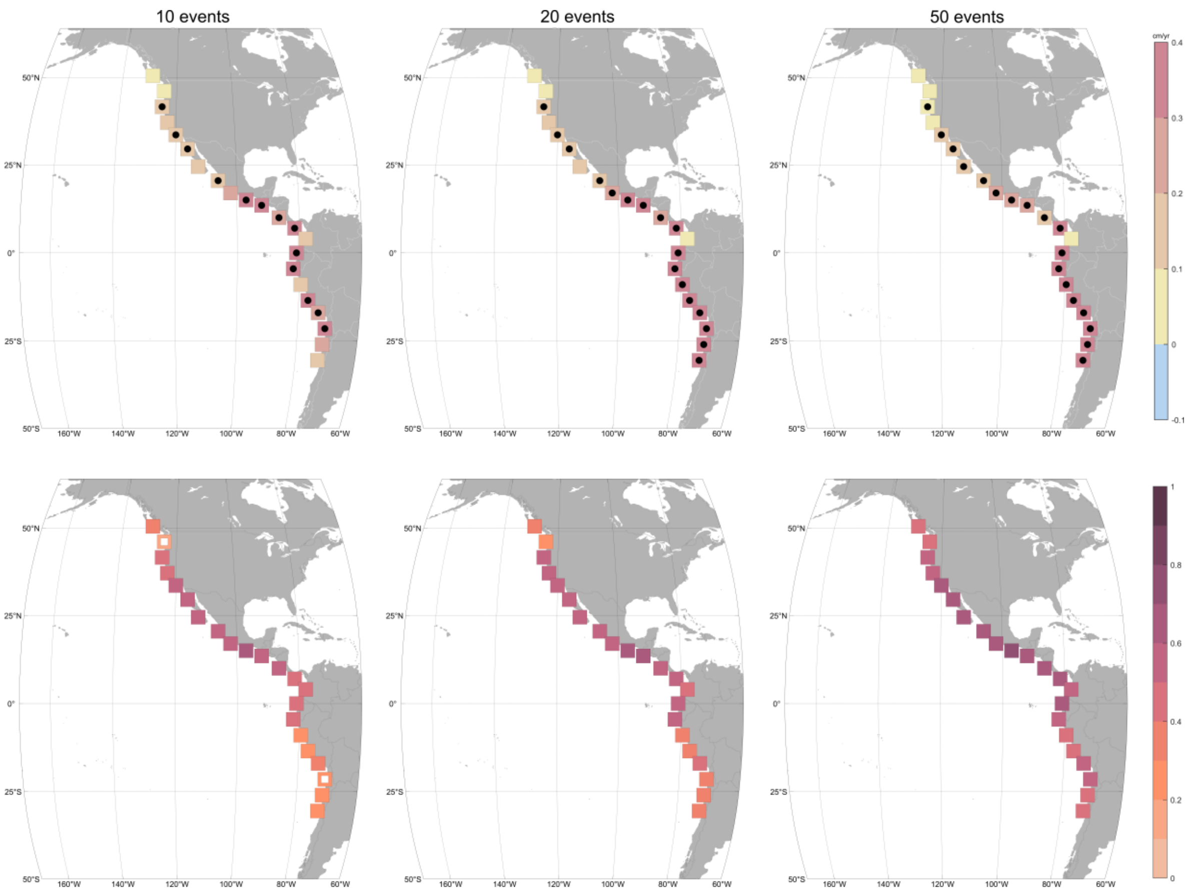


We have modified the methods section in the new version of the manuscript:
L594-597: *"For the total calculation, the mean was derived from the 900 recorded events, which were based on the 20 most powerful events per year over a 45-year period. The sensitivity was analyzed using sample sizes of 10 and 50 events, and consistent results were obtained across all cases, supporting the robustness of the selected 20 events."*

- Figure SM1: The southernmost point the authors defined is at 30.5°S latitude. Can this be extended further South? With the current configuration the authors are missing the area from Chiloé Island to La Serena, which have a considerable population density (https://sedac.ciesin.columbia.edu/downloads/maps/grump-v1/grump-v1-population-density/chldens.jpg). Viña del Mar, a very popular tourist destination which has been affected by extreme wave events in the past (e.g.,: https://www.infobae.com/america/america-latina/2024/12/29/un-fuerte-oleaje-sorprendio-a-los-turistas-en-vina-del-mar-y-las-autoridades-chilenas-activaron-medidas-de-seguridad/) is at 33°S and therefore not included in this analysis.

Thank you for the comment. We deliberately limited the analysis to north of 30° S to avoid entering generation regions. Indeed, the southernmost points already show that many of

the metrics analyzed become less clear. This is mainly because these areas lie within swell generation regions, where multiple events occur simultaneously, often overlapping and merging into a single system. As a result, conditions are not as well defined and the algorithm performs less reliably there. Extending the analysis further south would likely exacerbate these limitations and yield less robust results, even though we acknowledge the relevance of the reviewer's suggestion regarding the densely populated Chilean coast.

- Figure SM3: Please mention what the grey and red points mean in the caption. These results show a clear positive bias (overestimation) by the model, which could be discussed.

Thank you for the comment. We have revised the figure and the figure caption and added a discussion of the bias to the manuscript.

*L168:173: "The results show strong correlations (mean value > 0.84), moderate root mean square errors (RMSEs; mean value < 0.11 m) and slight positive biases (mean value < 0.03 m) for all buoys when daily incoming wave energy throughout the study period is compared (gray dots in Figure SM3). Model output performance improves further when focusing specifically on the daily energy recorded during the selected SO swell events at the buoy locations (red dots in Figure SM3)."*

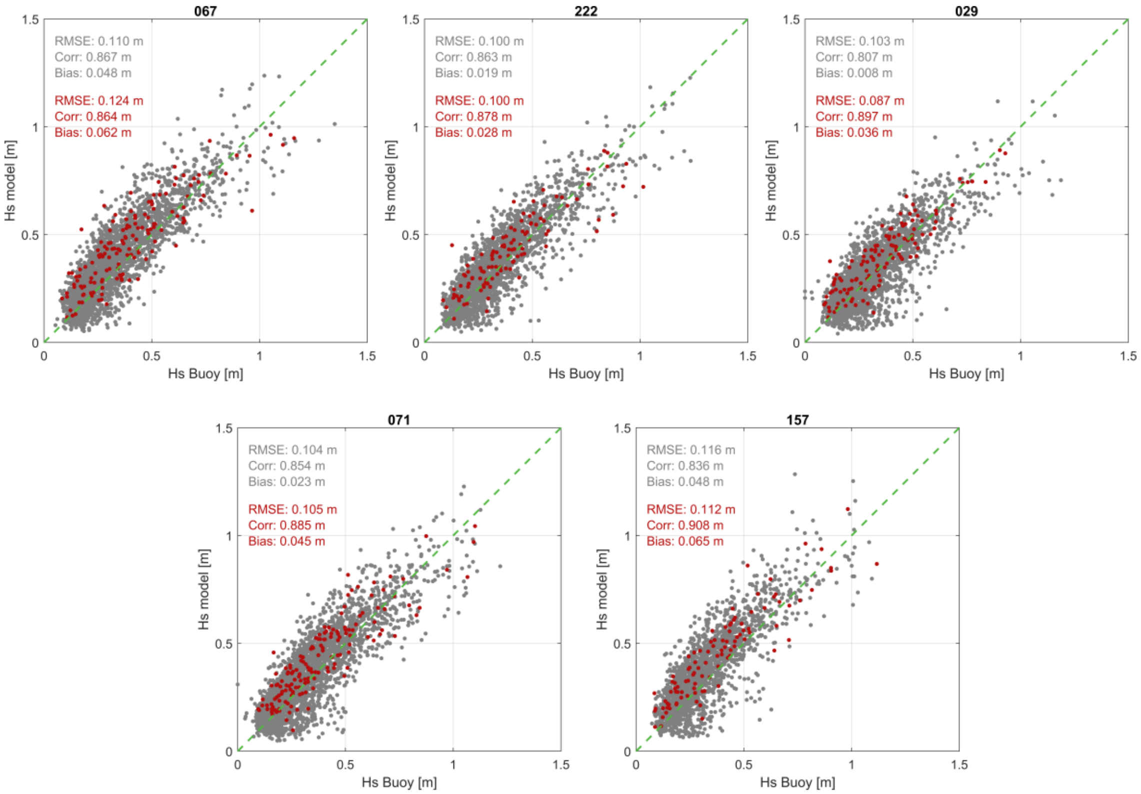

***Figure SM4****: Validation of wave energy from the Southern Ocean for five buoys managed by CDIP. Gray dots show the daily equivalent significant wave height associated with Southern Ocean energy. Red dots show the daily equivalent significant wave height recorded during the selected SO swell events at the buoy locations.*

- Lines 206-208: The pioneering study of Hemer et al. (2010) should also be included as a reference here.

Hemer, M. A., Church, J. A., & Hunter, J. R. (2010). Variability and trends in the directional wave climate of the Southern Hemisphere. International Journal of Climatology: A Journal of the Royal Meteorological Society, 30(4), 475-491.

Thank you very much for the suggestion. We have included this important reference in the manuscript.

- Figure SM4: The caption needs fixing; the grey and black arrows represent different things.

Thank you for the comment. We have corrected the figure caption.

***Figure SM5****: Differences in the mean significant wave height (H within the square), peak period (T within the square), and peak direction (arrows) of the SO swell events compared to the overall conditions at the time of the arrival of the events. Pink arrows indicate the directions associated with total wave conditions; black arrows indicate the directions associated with SO swells.*

- Line 222: Why not using the 95% confidence interval as it's usually done? How would the results change?

Thank you for the comment. This point has already been addressed in a previous response.

- Lines 226-228: "Trends in wave period" -> Where are they? Can they be added to Figure 4 even if only statistically significant at 55% of the locations?

Thank you for the comment. We have added this figure to the Supplementary Material. In response to another reviewer's suggestion, we also updated the method used to calculate the slopes, which improved robustness. As a result, 64% of the points now show a significant change. However, the slopes for Tp remain mild, with maximum values of about 0.1 s per decade.

L254-256: *"In contrast, trends in wave period, although always positive, are smaller and statistically significant at fewer locations, with statistical significance at the 90% level reached at 64% of them (Figure SM6)."*

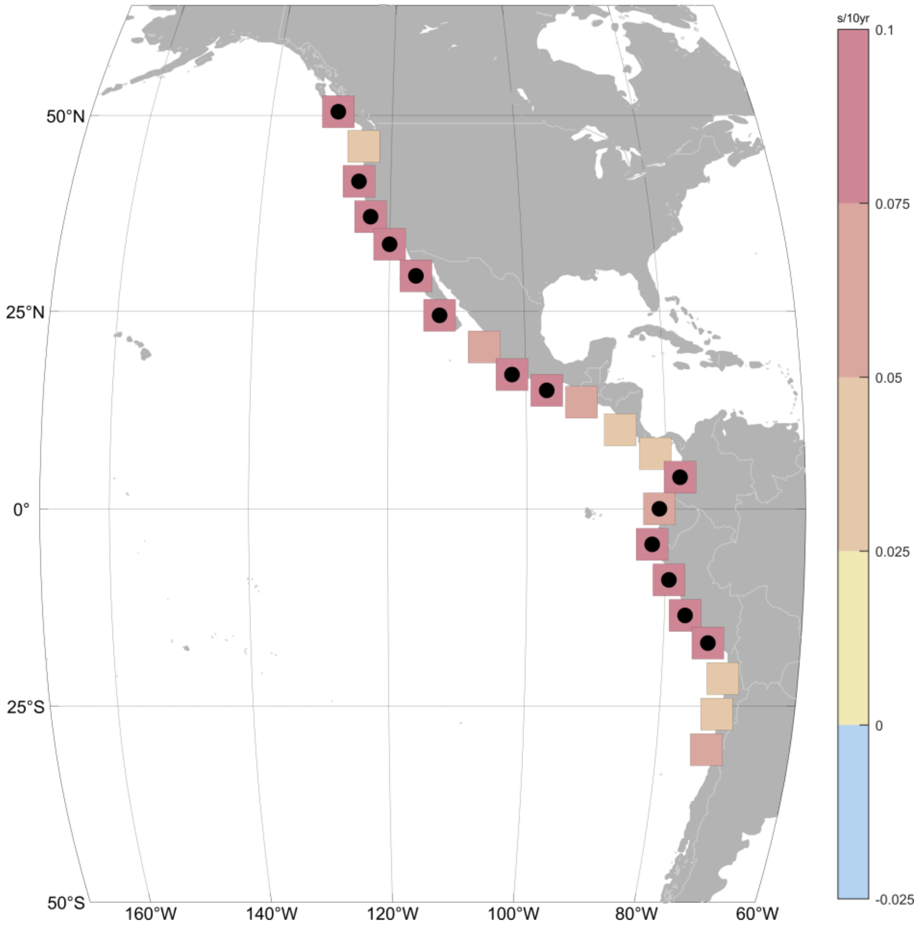


***Figure SM6**: Annual trend in peak period expressed per decade. The black dots indicate statistical significance at the 90% confidence level.*

- Lines 323-324: "As a result, coastal water levels in areas affected by these swells are likely to show an accelerating positive trend due to the interaction of these two dynamics."… I don't follow this logic.

Thank you for the comment. The logic behind this sentence is that in areas affected by these swells, extreme coastal water levels associated with their arrival are not only expected to increase due to the long-term trend in swell intensity, but will also be compounded by the concurrent mean sea level rise.

We have revised the sentence:

L376-379: *"As a result, extreme coastal water levels associated with these swells are likely to exhibit an accelerating positive trend due to the interaction of these two dynamics, thereby increasing the hazard for coastal communities and infrastructure."*

- Lines 405 and 407: I suggest changing "corresponding to" to "associated with".

Thank you for the comment. We have changed the sentence accordingly.

- Line 420: in the employed numerical modelling -> change to "in the employed numerical model"

Thank you for the comment. Done.

- Line 429: remove the word "secondary"

Thank you for the comment. Done.

- In the Supplementary Material, in the "Swell identification algorithm" section, consider incorporating a formula for the weighted Euclidean distance, it might help improve the flow of the reading (see for example Jiang, 2019)

Jiang, H. (2019). Spatially tracking wave events in partitioned numerical wave model outputs. Journal of Atmospheric and Oceanic Technology, 36(10), 1933-1944.

Thank you for the comment. We have added the formula for the weighted Euclidean distance to the method description in the Supplementary Material.

*"Event identification: Each selected peak is used as the starting point for the algorithm. The algorithm identifies the next most probable data point from all available options, step by step, defining the descending $T_p$ line that characterizes the swell event. The next most likely data point is sought within the next 8 hours, as discontinuities may occur. The next point is selected using a weighted Euclidean distance approach as the closest one in a three-dimensional $T_p$-$H_s$-$time$ space. Distances are calculated using normalized $H_s$ and $T_p$ variables and time in hours. The weight assigned to the $T_p$ variable is 1 in all cases, while the weight for the $H_s$ variable is always 0.1 (its purpose is simply to distinguish the closest cases). Lastly, the weight associated with the time variable is determined as the linear slope formed by the previously selected points (with a minimum weight value of 0.01). The*

*main goal is to assign equal weight to the time and $T_p$ variables. This means that if the slope is steeper, the weight assigned to time will be greater, and vice versa.*

$$d = F_1 \cdot T_p + F_2 \cdot H_s + F_3 \cdot time \qquad \text{Eq. SM1}$$

*where $F_1$ = 1, $F_2$ = 0.1 and $F_3$= [0.01, Inf].”*

REVIEWER COMMENTS

**Reviewer #1** (Remarks to the Author):

In this new version, the authors seriously addressed the comments raised by the two reviewers and their paper improved substantially. I only have a few last minor suggestions (please note that I refer to line numbers in the clean revised version):

We thank Reviewer #1 for the thorough and constructive assessment of the manuscript. We particularly appreciate the detailed comments and suggestions, which helped us to substantially improve the clarity, robustness, and overall presentation of the study. The reviewer's input has been instrumental in refining both the methodological description and the discussion of the results.

Despite the absence of specific comments in this review, and in line with an editorial request and comments from the previous review, we wish to emphasize that the article title has been modified to more faithfully reflect its content: *"Four decades of intensifying Southern Ocean swells along the Pacific coast of the Americas."*

-in their rebuttal, the authors state that no wave buoy data in the Southern hemisphere meet with their quality requirement (« a minimum temporal coverage of 10 years and a maximum time gap of 40% »). This is not true, for instance data from the buoy « Stratus » (WMO 32012, off the Chile/Peru border, easily accessible through the Copernicus portail https://marineinsitu.eu/dashboard/) are available from 2007 to 2018 and has less than 10% of gaps. Furthermore, this data includes several events with Hm0 reaching 5 to 6 m, including the event of May 2015 discussed by the authors in their introduction. Next I found on figure SM10 that this buoy as well as WMO 32302 were apparently included in their validation procedure : please check and clarify. Could you show how the event of May 2015 s reproduced in your hindcast ?

Thank you for the comments. With these statements we referred to the fact that no buoys were available in the Southern Hemisphere with sufficiently detailed spectral information for a robust validation of Southern Ocean swells, as described in *Methods/Validation* section. Nevertheless, we have re-examined the use of data from buoy 32012 for this purpose and have finally been able to include it, together with three additional spectral buoys.

The issue with this buoy, and with the rest of the spectral buoys operated by the NDBC network, is that the provided spectral data include only the first two non-zero terms of the Fourier harmonic expansion of the directional spectrum (i.e., the harmonics of order n = 1 y n = 2). This truncation of the series limits the angular resolution of the spectrum and leads, after reconstruction, to unrealistically broad directional spreading. Even for mature swell systems, the reconstructed directional width is excessively large.

When applying the proposed validation method and comparing the energy coming from the Southern Ocean with that of the hindcast, a large portion of the energy spread outside the directional sectors corresponding to the Southern Ocean as a consequence of this artificial over-spreading. Therefore, the comparison with the buoy was not entirely "fair," since the

reconstruction of the buoy spectrum could not be performed rigorously with the limited information provided.

To address this, we computed from the model directional spectra the first and second harmonic Fourier coefficients for each frequency $f$:

$$a_n(f) = \int_0^{2\pi} D(f,\theta)\cos(n\theta)\,d\theta,$$

$$b_n(f) = \int_0^{2\pi} D(f,\theta)\sin(n\theta)\,d\theta,$$

with n = 1,2, where $D(f,\theta)$ is the normalized directional spectrum.

From these coefficients, we derived the equivalent parameters published by NOAA, following the formulation of Longuet-Higgins et al. (1963) as adopted by the NDBC:

$$R_n(f) = \frac{\sqrt{a_n(f)^2 + b_n(f)^2}}{a_0(f)},$$

$$\alpha_1(f) = 270 - \frac{1}{n} atan2(b_1(f), a_1(f))$$

$$\alpha_2(f) = 270 - (\frac{1}{2} atan2\big(b_2(f), a_2(f)\big) + \{0\ or\ 180\})$$

where $a_0(f)$ is the zeroth-order moment.

Using these parameters ($R_1, R_2, \alpha_1, \alpha_2$), we reconstructed a model spectrum equivalent to the buoy spectrum by applying the exact NOAA reconstruction equation (see https://www.ndbc.noaa.gov/faq/measdes.shtml). In this way, both datasets (buoy and model) share the same directional-spreading limitations, ensuring a more consistent and equitable comparison.

As a result, the validation exhibits a marked improvement and a much more coherent agreement between the Southern Ocean energy identified in both sources. In addition to buoy 32012, three other spectral buoys were included, providing broader coverage of the eastern Pacific, as illustrated in the following figure. Figure SM3 has been updated accordingly.

We also note that buoy 32302 was indeed used in the validation of significant wave height.

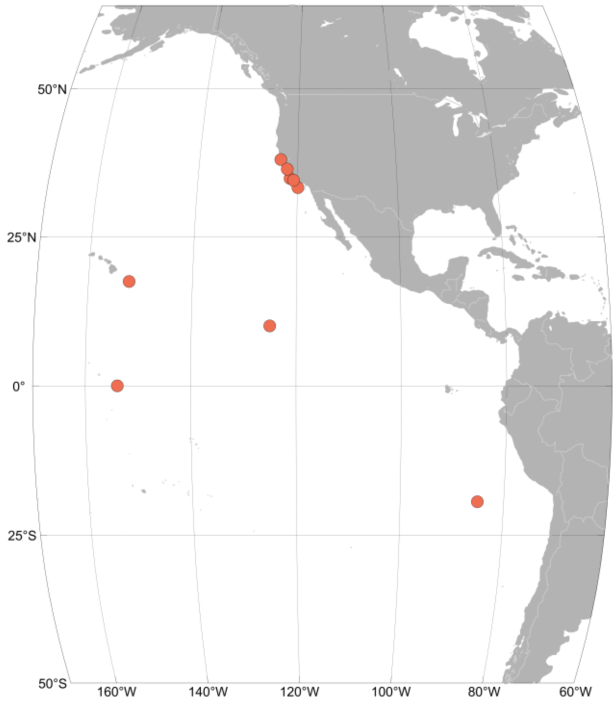


Regarding the 2015 event, the following figure shows the time series of significant wave height from both the model and the buoy at the 32012 buoy location. The results show a good agreement between the two sources.

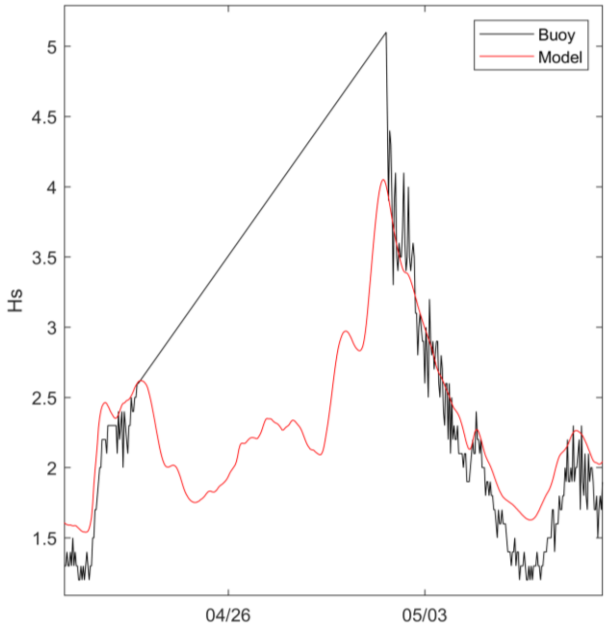


-L25 : « interaction » is not fully appropriate in this context, do you mean « coincidence » ?

Thank you for the comment. We have corrected the sentence accordingly.

-L36 : please rephrase « waves …with waves »

Thank you for the comment. We have corrected the sentence accordingly:

L37-40: *"For example, waves arriving at the coast of California can be generated by low-pressure systems in the middle of the North Pacific Ocean, with waves reaching the shore as well-organized waves, or by a land-falling tropical storm, with waves impacting the coast in a convoluted and chaotic manner[4]."*

-L204 : how do you explain that a highest proportion of SO swells reach the southern coast of Mexico and Guatemala in MAM and not JJA as in other locations ? Could it be a matter of wave direction, e.g. coming more from the south in MAM compared to JJA ?

Thank you for this comment. We note first that Figure 2 does not show a clear dominance of MAM over JJA along the southern coast of Mexico and Guatemala; rather, the contributions during MAM and JJA are comparable. We have revised the text accordingly to avoid overstating this difference.

A possible interpretation of this regional behavior, beyond the scope of the present study, is related to the seasonal and longitudinal variability of Southern Ocean cyclone activity. In particular, previous work by Lixin and Ting (2016) shows that during austral autumn (MAM), explosive cyclones in the South Pacific tend to be preferentially located further east. Swells generated in these eastern sectors can reach Central America but are geometrically constrained from propagating toward higher latitudes along the North American coast, which may explain why MAM contributions appear locally comparable in this region but not further north.

During austral winter (JJA), Southern Ocean cyclones are both more energetic and more favorably distributed longitudinally, allowing swells to impact a broader range of latitudes and leading to a clear winter dominance along much of the coast. At southern latitudes, closer to the generation region, the seasonal cycle of Southern Ocean wave activity is more directly reflected in coastal wave conditions.

A robust assessment of this hypothesis would require a dedicated analysis explicitly linking the seasonal and spatial variability of Southern Ocean cyclone activity with swell generation, propagation pathways, and coastal wave impacts. Such an integrated wave–cyclone analysis, including a more exhaustive seasonal characterization, is beyond the scope of the present study, which focuses on the identification and characterization of Southern Ocean swell events at the coast.

L211-214: *"The highest proportion of events is observed during the austral winter (June to August: JJA) at most locations, the only exception being the southern coast of Mexico and Guatemala, where the proportion during the austral autumn (from March to May: MAM) is comparable."*

-caption figure SM3 : the variable displayed is apparently Hs and not wave energy.

Thank you for the comment. We have modified the figure caption to more accurately reflect the validation, as well as the labels of the figure panels.

***"Figure SM1**: Validation of wave energy, measured through the parameter $H_s^e$ (Eq. 2, main text), from the Southern Ocean for five buoys managed by CDIP. Gray dots show the daily equivalent significant wave height associated with Southern Ocean energy. Red dots show the*

*daily equivalent significant wave height recorded during the selected SO swell events at the buoy locations."*

**Reviewer #2** (Remarks to the Author):

We thank Reviewer #2 for the thoughtful and insightful comments. We appreciate the reviewer's careful reading of the manuscript and the suggestions aimed at improving transparency, consistency, and interpretation, which have helped to clarify key aspects of the analysis and strengthen the overall narrative of the paper.

In line with an editorial request and comments from the previous review, we wish to emphasize that the article title has been modified to more faithfully reflect its content: *"Four decades of intensifying Southern Ocean swells along the Pacific coast of the Americas."*

The authors have made substantial progress since the first round of reviews, including:

• Terminology and definitions have been clarified. For instance, authors now better distinguish between "potential for hazards" and "hazardous events" consistently across the manuscript.

• Revisions improved clarity and transparency on assumptions and methodological choices, such as thresholds and event selection criteria.

• The shift from calendar to ENSO-year definitions appropriately captures full ENSO phase transitions within the same analysis period.

• Authors now use a more robust approach to assess interannual variability, replacing the previous end-point method, which was pointed out as potentially problematic. This corrected the earlier overemphasis on interannual variability, and seasonal variability emerged as the dominant mode of variability instead of interannual variability.

Remaining Concerns and Suggestions

• If Table 1 was used to help develop the algorithm and validate impactful events, then it would be useful if it included the wave characteristics (Hs, Tp, and Dp) associated with the Southern Ocean (SO) swells events. Suggesting that authors indicate which events were used in this validation and to potentially add extra columns with the average ± standard deviation of wave height, wave period, and wave direction; this would make the table more than introductory, but also a methodological and interpretive reference point.

We thank the reviewer for this thoughtful suggestion. We clarify that Table 1 only provides the timing, location, and documented impacts of reported events, which are the sole pieces of information used in the comparison with the algorithm output. The wave characteristics associated with these events (e.g., wave height, period, and direction) are not used as inputs to the method. As described in the manuscript, available wave data (buoys and hindcasts) were examined independently and solely to confirm the Southern Ocean origin of the reported events in those cases where the origin was not explicitly stated in the original reports, prior to their inclusion in Table 1. The table is then used to assess whether the algorithm independently identifies these events based only on spectral information, thereby avoiding any form of circular validation.

We considered the possibility of including event-specific wave characteristics in Table 1, as suggested by the reviewer. However, we ultimately chose not to do so, as such information is not used in the event selection or validation step, and its inclusion could inadvertently suggest that these wave properties were available a priori or informed the algorithmic identification.

Instead, we now include in Table SM2 the swell characteristics associated with the events listed in Table 1, as obtained from the algorithm. These event-specific characteristics are provided as descriptive information and are referenced in the main text in the "*Main swell characteristics"* section to complement the aggregated statistics shown in Figure 2.

L207-208: *"For completeness, the individual swell characteristics associated with the events listed in Table 1 are provided in Table SM2"*

• Some of the sources aren't actually sources. For example, patch.com is a general community-specific news site across the United States. There over 30,000 community-level patch news providers (or so the website says). Can the authors put the actual reference link for where this was found? This goes for the other generic references in this table too.

We thank the reviewer for this comment. We agree that citing generic platforms (e.g., Patch.com) does not provide sufficient traceability. In the revised version of Table 1, we have replaced all generic platform references with the full and explicit links to the specific news articles or official reports where each event was documented. This ensures that every entry in the table is directly traceable.

• L135–136: The language used here suggests that all the points are deep enough to avoid shoaling processes, even though the rebuttal (pg 17) states that may not be the case for two nodes, which may or may not show variation outside of the trends seen across the broader region. So, while it's true that the majority of locations (and those two locations) are at a depth that is "sufficient to avoid substantial shoaling processes" an acknowledgement of the two locations that may effected by shoaling processes would be more transparent as the text says the avoid them, and because on average these waves are 15 s, which means a good amount of them have longer periods than that too.

We thank the reviewer for this comment. We agree that the original wording could be interpreted as implying that all locations are entirely unaffected by shoaling processes. We have therefore revised the manuscript to clarify this point.

Specifically, we now describe in the Methods section the selection of the 22 offshore nodes used in the hindcast analysis and explicitly identify the two locations where some shoaling influence may occur, particularly for longer-period waves. This information has been added for transparency. In the main text, the wording has been adjusted to state more generally that the influence of shoaling is limited at the selected locations.

L138-141: *"These points are representative of the entire coastal area while ensuring sufficient distance from wave-breaking shallow water zones. At the same time, the points are located at depths sufficient to minimize, even for long-period waves ($T_p$ longer than 14 s), any pronounced shoaling processes."*

L528-532: *"Wave parameters were extracted at 22 coastal target points (**Figure SM1**). Most locations are situated at depths sufficient to avoid substantial shoaling effects for the dominant wave periods considered (**Table SM1**). Two points (P2 and P17), however, are located at shallower depths where some limited effects may occur, particularly for longer-period waves.*

*Importantly, the inclusion of these nodes does not affect the regional-scale patterns discussed in this study."*

• L185 – 188: The way this line is written, it's unclear whether SO swell wave height or total wave height is larger. It's unclear from SM4 too, whether SO swell wave height is being subtracted from the total wave height, or vice versa.

We thank the reviewer for pointing this out. We agree that the original wording was unclear and could lead to ambiguity regarding the definition of the difference shown in the text and in Figure SM4. The original formulation implicitly assumed that the total wave height would always be larger than the Southern Ocean swell wave height, but we agree that this assumption was not made explicit and that the revised wording is clearer. We have therefore modified the text and the figure caption accordingly to explicitly clarify that the difference refers to the total wave height minus the Southern Ocean swell wave height.

L190-206: *"The difference between the total wave height and the SO swell wave height at the moment these events reach the coast can be as high as 2 m at the northernmost locations (**Figure SM4**). However, in areas closer to the SO, where these events often dominate the wave climate, this difference is considerably lower (< 0.25 m). Note that the differences shown in **Figure SM4** are computed relative to the total wave conditions at the time of SO swell arrival and do not necessarily represent the contrast with local maximum wave conditions.*

*Unlike wave height, the period of these swells increases northward, as only the longest-period swells can travel the greatest distances. Along the Pacific coasts of the Americas, characteristic $T_p$ values generally exceed 15 s, with minor variations along the coastline, primarily following a south-to-north increasing gradient. Similar to wave height, the differences between the total wave periods and the SO swell periods at the moment of SO swell arrival are more pronounced along the coast of North America. In this region, the total $T_p$ tends to be significantly shorter (by more than 3 s) than those associated with SO swells.*

*Although the swell incoming direction is predominantly from the southwest at all locations, the southern component becomes increasingly prominent relative to the western component with distance northward. The directional offset of SO swells relative to the total incoming wave direction at the time of SO swell arrival becomes more pronounced as the relative energetic contribution of these swells decreases. This offset exceeds 45° along the coast of California, whereas it is negligible along the coast of Chile."*

*"Figure SM4: Differences in the mean significant wave height (H within the square), peak period (T within the square), and peak direction (arrows) of the SO swell events compared to the overall conditions at the time of the arrival of the events, computed as the total wave parameters minus the SO swell–associated parameters. Pink arrows indicate the directions associated with total wave conditions; black arrows indicate the directions associated with SO swells."*

• L216 and L625: If I'm understanding correctly, the annual values of the MEI index was calculated based on an ENSO year definition of May-April, but the annual wave conditions were computed over the calendar year. Could this potentially be a reason for less strong

correlation? If waves were recalculated to reflect the same time period ENSO is being defined, would the correlation change?

We thank the reviewer for this comment. The reviewer is correct in noting that the original analysis compared ENSO indices defined using an ENSO-year framework (May–April) with wave metrics aggregated over calendar years, which introduced an inconsistency in the temporal definition. We have revised the analysis to ensure full consistency between the ENSO index and the wave-event metrics, by aggregating both using the same May–April (ENSO-year) definition.

This revision leads to an overall improvement in the results. Specifically, correlations increase at 12 of the 22 analyzed locations, while they decrease at the remaining 10 locations. Importantly, the magnitude of the improvements at the former locations is substantially larger than the magnitude of the decreases observed at the latter, resulting in a net strengthening of the ENSO–wave relationship.

The manuscript has been updated accordingly, including revisions to the relevant text and figures, to reflect this consistent temporal framework and the updated results.

• L586: The section here should explicitly state that these methods are performed by individual sites. This is particularly important for the Frequency of Occurrence threshold.

We thank the reviewer for this comment. We agree that it should be stated explicitly that all indicators are computed independently at each coastal target point.

L614-616: *"To analyze swell events from the SO, a series of related indicators were used, all of which were computed independently at each coastal target point, both to study the properties of the events and to assess their variability at different time scales."*

• The discussion in L225–241 may needs some revision. It is inconsistent to describe correlations of 0.6–0.7 (maximum correlations observed) with SAM as "high" but correlations < 0.6 with ENSO as "weak," when the threshold values overlap. The interpretation should be consistent. For example, potentially point out lower correlation values for ENSO and less significance, and highlight the locations that have a strong correlation too.

We thank the reviewer for this comment. We agree that the qualitative interpretation of correlation strength should be presented consistently. To avoid any potential ambiguity, we have revised the text by removing qualitative descriptors for ENSO and focusing on a purely quantitative description of the correlation values, their statistical significance, and their regional distribution. This ensures a consistent and transparent comparison with the SAM-related results.

L231-246: *"The results indicate a strong correlation between the annual mean wave power of the selected events and the positive phase of the SAM, with all analyzed locations showing significant correlations at the 90% confidence level (**Figure 3**). During this phase, westerly winds in the Southern Hemisphere (SH) intensify and shift poleward, along with extratropical cyclones generated in this region[62,63], which likely contribute to increased wave power in events generated south of 40°S. These findings align with previous studies showing a strong correlation between the positive phase of this index and both mean and extreme wave heights*

*in the SO and eastern Pacific Ocean[64–67]. The observed correlations along the Pacific coast of the Americas are particularly relevant north of the equator, consistently exceeding 0.5. The maximum values are found along the coast of Guatemala and the Southernmost coast of Mexico, ranging from 0.6 to 0.7. South of 5°S, the correlation values fall below 0.4 at most locations.*

*Correlations with the negative phase of ENSO (the Niño phase) were also identified. Correlation values remain below 0.6 across all analyzed locations (**Figure 3**), with 82% of them being statistically significant at the 90% confidence level. The most robust results can be observed along the coasts from Peru to Mexico, with values ranging between 0.35 and 0.65. These findings align with previous research[65,66] indicating a correlation between the negative phase of ENSO and the positive phase of the SAM, leading to increased wave heights in the southeastern Pacific during negative ENSO phases."*

• Lines 313 – 327: One thing to note here, especially in relation to the NH. The largest waves (height and period) from a hazard perspective in Washington, Oregon, and Northern California occur in December, January, February, when there are very few SO swells occurring (Figure 2 right). These waves seem much larger than those from SO swells, and likely what are used for coastal hazards protection/design. So disregarding them SO swells in analysis potentially won't lead to an underestimation of coastal risk in design in this location. I don't think Figure 4SM would bring up the true difference between waves from DJF compared to the SO swell, because they're not really occurring at the same time. See some citations below with general wave climatology for the region.

García-Medina, G., Özkan-Haller, H. T., & Ruggiero, P. (2014). Wave resource assessment in Oregon and southwest Washington, USA. Renewable Energy, 64, 203-214.

Allan, J. C., & Komar, P. D. (2006). Climate controls on US West Coast erosion processes. Journal of coastal research, 22(3), 511-529.

We thank the reviewer for this insightful comment. We agree that Figure SM4 was not well aligned with the original formulation of this paragraph. That figure is intended to illustrate wave conditions occurring simultaneously at the time of Southern Ocean (SO) swell arrival, whereas the purpose of the paragraph is to discuss broader, climatological aspects of wave-system dominance and the implications for coastal risk. We have therefore revised the text to remove any ambiguity and to avoid using Figure SM4 in support of an argument it was not designed to address.

We also clarify that when referring to SO swells as "secondary," we do not mean secondary swell systems in the sense of concurrent swell components (often referred to as swell-2), but rather secondary in a climatological sense, i.e., not dominant in the long-term wave climate at a given location. This distinction is now made explicit in the revised text.

Importantly, we do not intend to suggest that SO swells control design-level wave conditions in the Northern Hemisphere, where boreal winter North Pacific storms dominate coastal design considerations. Instead, the revised discussion emphasizes that SO swells represent a non-dominant but potentially hazardous wave system, particularly outside the climatologically dominant wave season (e.g., during boreal summer), when exposure is higher and hazard

perception is reduced. These events are therefore not readily captured by approaches based solely on integrated wave parameters or seasonal design conditions, and their identification requires a spectral-based framework such as the one proposed here.

We believe that the revised text more accurately reflects this intent and addresses the reviewer's concern.

L317-333: *"While SO swells dominate the wave climate in the SH[16], their relative importance is greatly reduced in the NH, where North Pacific-generated waves are the dominant wave systems[68,69]. This decrease is evident even when comparing total wave conditions with SO swell characteristics at the time of arrival (**Figure SM4**). The spatial contrast in relative importance influences the regional perception of SO swells and their potential hazards, increasing the likelihood of being overlooked, particularly when they arrive in seasons that do not coincide with those associated with the dominant wave systems. Typically, the wave climate at a given location consists of waves generated in different ocean regions[54,70]. In most cases, one or two primary wave systems are well known and studied, and coastal risk management strategies are typically developed accordingly. However, overlooking potentially hazardous non-dominant systems, such as SO swells, even when they occur outside the climatologically dominant wave season, could have severe consequences. Although appearing as secondary components of the wave climate in certain regions, particularly in the NH, their disregard could lead to an underestimation of coastal risk, primarily due to increased and insufficiently accounted-for exposure. These swells possess substantial energy or may exhibit notable directional deviations from dominant conditions, increasing the potential for significant impacts when their occurrence is not anticipated. This highlights the need for a comprehensive characterization of the wave climate that goes beyond traditional approaches based on integrated parameters, as these often mask the presence of remote swells[71]."*

• SO swell impact to hazards would maybe increase them at times of the year there wouldn't be as likely to have hazards. I wonder, for example, the role they play in sneaker type waves (e.g., Garcia-Medina et al., 2018)

García-Medina, G., Özkan-Haller, H. T., Ruggiero, P., Holman, R. A., & Nicolini, T. (2018). Analysis and catalogue of sneaker waves in the US Pacific Northwest between 2005 and 2017. Natural Hazards, 94(2), 583-603.

We thank the reviewer for this interesting suggestion. While it cannot be ruled out that some hazardous wave episodes, such as sneaker-type waves, may occasionally be associated with Southern Ocean swells, establishing such a link would require a dedicated analysis that is beyond the scope of the present study. Based on the information provided by García-Medina et al. (2018), including the documented timing of sneaker wave events, we do not consider there to be sufficient evidence to establish a causal relationship between these phenomena and SO swell activity. Nevertheless, we agree that exploring potential connections between remote swell systems and sneaker wave occurrence is an interesting topic and would merit further investigation in future studies.

• Christie Hegermiller has a few papers that seem to back up some of these points for Southern California – that SH generated swell can contribute a lot to local waves, especially in

JJA. (Figure 2 in the GRL paper referenced below has a nice diagram of this). Figure 1 shows how the contribution can vary at a high resolution locally as well – which might be a nice point to bring up based on the coarseness of the results presented within this manuscript.

Hegermiller, C. A., Rueda, A., Erikson, L. H., Barnard, P. L., Antolinez, J. A. A., & Mendez, F. J. (2017). Controls of multimodal wave conditions in a complex coastal setting. Geophysical Research Letters, 44(24), 12-315.

Hegermiller, C. A., Antolinez, J. A., Rueda, A., Camus, P., Perez, J., Erikson, L. H., ... & Mendez, F. J. (2017). A multimodal wave spectrum–based approach for statistical downscaling of local wave climate. Journal of Physical Oceanography, 47(2), 375-386.

We thank the reviewer for highlighting these studies. We agree that the work by Hegermiller et al. (2017a, b) is highly relevant and provides a rigorous local-scale perspective on the contribution of Southern Hemisphere–generated swell to the wave climate along the Southern California coast, particularly during boreal summer. These studies offer strong support for the broader discussion presented in our manuscript regarding the presence and relevance of Southern Ocean swells in the Northern Hemisphere.

In particular, the high-resolution analyses and multimodal spectral framework adopted by Hegermiller et al. demonstrate how remote swell systems can make a substantial and spatially variable contribution to local wave conditions, reinforcing the physical basis of our basin-scale findings. While our study does not aim to resolve such fine-scale coastal variability, these works provide valuable complementary evidence that adds rigor to the discussion of SO swell influence in the NH.

• L362–364: Specify the period over which the positive SAM trend is observed to better clarify why it may relate to increased SO swell energy.

We thank the reviewer for this comment. We have revised the text to explicitly specify the period over which the positive trend of the SAM is discussed. The manuscript now clarifies that the increase in SO swell energy is considered in the context of the positive SAM trend observed during the latter half of the twentieth century, as documented by King et al. (2023).

L371-374: *"These observed increases in SO swell characteristics are consistent with increases in wind speed in the SO over recent decades[73,80], suggested increases in storm activity[75], and a positive trend in the SAM in the latter half of the twentieth century[81]."*

• L646–648: The explanation of how seasonal variability is computed could be clarified — especially how seasonal means are derived. Are seasonal means calculated per year and then averaged, yielding four mean values (Winter, Spring, Summer, Fall)?

We thank the reviewer for this comment. Seasonal variability is computed by grouping the identified events according to the season in which they occur and then calculating the corresponding mean values across all events within each season. Seasonal means are therefore not computed on a year-by-year basis and subsequently averaged, but rather derived directly from the full set of events assigned to each season. We have clarified this point in the text to avoid potential ambiguity.

L677-680: *"Seasonal variability was computed as the mean of the relative difference between the maximum and minimum seasonal means* $P_{wp}$ *for the study period across the four seasons, normalized by the study period's mean value. Seasonal means were calculated by grouping all identified events according to the season in which they occur and averaging* $P_{wp}$ *across events within each season."*

• Figures in general: Labeling panels (e.g., a, b) would be better than indicating location (e.g., left, right), especially when the number of panels increases (e.g., Figure 4). Having to keep track of top-left,top-right,bottom-left becomes slightly less easy to read.

We thank the reviewer for this suggestion. We agree that labeling panels using letters improves clarity, particularly for multi-panel figures. All figures have been revised accordingly to include panel labels (e.g., a, b, c), replacing references based on panel position.

• Figure 2 – text cut off from right panel figure.

We thank the reviewer for pointing this out. The text cut off in the right panel of Figure 2 has been corrected in the revised manuscript.

• Data Availability statement information about access to the dataset analyzed in the study is provided, but information about access to the data generated during the study are not provided.

We thank the reviewer for this comment. We have revised the Data Availability statement to explicitly specify access to the datasets generated during this study. The statement now clarifies that these derived datasets are available from the corresponding author upon reasonable request.

*"The data that support the findings of this study, including both the datasets analyzed and those generated during the study, were produced at IHCantabria and are available for research use upon reasonable request."*

**Reviewer #3** (Remarks to the Author):

I think the manuscript is substantially improved, particularly the introduction and overall presentation of the context of this study and implications. Also, I thank the authors for the thorough response to reviewers' comments.

We thank Reviewer #3 for the constructive and technically detailed feedback. The reviewer's comments helped us to better frame the scope of the study, clarify methodological choices, and place our results in the context of previous work, ultimately strengthening the rigor and positioning of the manuscript.

In line with an editorial request and comments from the previous review, we wish to emphasize that the article title has been modified to more faithfully reflect its content: *"Four decades of intensifying Southern Ocean swells along the Pacific coast of the Americas."*

There still could be better links between the why/what of the events of May 2015/May 2007 and the following paragraphs, but that is purely for readability and should be no barrier to publication.

Thank you for the comment. We have revised the introduction to improve the connection between the opening paragraph and the subsequent ones.

In my view, there are still a few, primarily technical, issues that need to either be addressed or that at least more thoroughly explained for the article to be considered rigorous.

Key among these is that the wave hindcast verification/validation is still insufficient. It would be much preferable if the "dedicated article describing the full validation" described in the response to reviewers was sufficiently complete to cite in this article. I understand sometimes logistics are such that papers are published out of preferred sequence, and that additional validation figures have been added to the supplementary materials, but it still doesn't answer questions about how this wave hindcst(the source data central to the findings of this study) compares to other contemporary efforts. Most important though, is the (continued) reliance on five buoys, clustered in a small region of the very large study area, for verification of spectral partitioning and swell tracking central to this analysis – the assertion in the response to the reviewers that other (e.g. NOAA) buoy did not "meet the required standards" is difficult to follow. What are the "required standards"? Jiang et al. (2016) applied a very similar swell tracking algorithm to multiple NDBC (NOAA) buoys to study swell arrival time, so it can definitely be done. Also, Portilla et al. (2009) –cited in this sudy - use a convolutional filter for noise removal. Perhaps these might help? More concise discussion on what the authors' issues around applying the spectral partition/swell tracking algorithm, and ideally broader validation with additional buoys would greatly improve the rigour of this paper.

We thank the reviewer for raising this point regarding the validation of the wave hindcast and its comparison with other contemporary global wave hindcast efforts. We agree that establishing such context is important, while also noting that a strict, metric-by-metric intercomparison across global hindcasts is not straightforward, as validation strategies, reported metrics (absolute versus normalized), spatial aggregation, and temporal coverage differ substantially among studies, and the underlying collocated datasets required to harmonize these metrics are not publicly available.

The wave hindcast used in this study has been validated against satellite altimeter observations at the global scale over the period 1991–2018, as well as against in-situ buoy measurements across the Pacific basin, as documented in the Supplementary Material. Global altimetry-based validation yields a global RMSE of approximately 0.3 m and negligible bias (on the order of a few millimeters), corresponding to a normalized RMSE of about 10%. These values are directly comparable to those reported by Liu et al. (2021) for a representative year (2011), who report an almost unbiased global significant wave height and an RMSE of about 0.3 m when validated against multi-mission satellite altimetry, as well as to the normalized error levels reported by Gaffet et al. (2025), who find global NRMSE values on the order of 10–15% with very small normalized biases.

L418-431: *"Additionally, it must be acknowledged that the hindcast used in this study, forced with ERA5, has been carefully calibrated and validated against both in-situ (buoys) and altimetry data. Altimetry-based validation indicates that the hindcast exhibits small overall errors, with absolute and normalized global metrics comparable to those reported in recent ERA5-based global wave hindcast studies, which typically report global errors on the order of a few tenths of a meter or normalized errors ranging between 10–15%, with small biases[82–84]. In addition, a dedicated validation of SO swell energy reaching the Pacific coast has been conducted using buoy spectral data. Nevertheless, despite the overall satisfactory performance, the use of this atmospheric forcing may still lead to some underestimation of wave conditions associated with extreme winds[83,84]. While this could affect the absolute magnitudes of the events, it should not compromise the main conclusions of this study. The applied methodology, which uses a variable threshold to identify events until a fixed number is reached, ensures that events are consistently detected even if their intensities are slightly underestimated. Together with the consideration of a sufficiently large sample of events each year, it provides robustness against systematic biases, ensuring that the detection of trends, interannual variability, and seasonality remains reliable."*

Regarding the specific validation of SO energy, in response to the reviewer's suggestion, we re-examined the available data and were able to include four additional spectral buoys for the validation of Southern Ocean swells.

The issue with the spectral buoys operated by the NDBC network, is that the provided spectral data include only the first two non-zero terms of the Fourier harmonic expansion of the directional spectrum (i.e., the harmonics of order n = 1 y n = 2). This truncation of the series limits the angular resolution of the spectrum and leads, after reconstruction, to unrealistically broad directional spreading. Even for mature swell systems, the reconstructed directional width is excessively large.

When applying the proposed validation method and comparing the energy coming from the Southern Ocean with that of the hindcast, a large portion of the energy spread outside the directional sectors corresponding to the Southern Ocean as a consequence of this artificial over-spreading. Therefore, the comparison with the buoy was not entirely "fair," since the reconstruction of the buoy spectrum could not be performed rigorously with the limited information provided.

To address this, we computed from the model directional spectra the first and second harmonic Fourier coefficients for each frequency $f$:

$$a_n(f) = \int_0^{2\pi} D(f,\theta)\cos(n\theta)\,d\theta,$$

$$b_n(f) = \int_0^{2\pi} D(f,\theta)\sin(n\theta)\,d\theta,$$

with n = 1,2, where $D(f,\theta)$ is the normalized directional spectrum.

From these coefficients, we derived the equivalent parameters published by NOAA, following the formulation of Longuet-Higgins et al. (1963) as adopted by the NDBC:

$$R_n(f) = \frac{\sqrt{a_n(f)^2+b_n(f)^2}}{a_0(f)},$$

$$\alpha_1(f) = 270 - \frac{1}{n}atan2(b_1(f), a_1(f))$$

$$\alpha_2(f) = 270 - (\frac{1}{2}atan2\big(b_2(f), a_2(f)\big) + \{0\ or\ 180\})$$

where $a_0(f)$ is the zeroth-order moment.

Using these parameters $(R_1, R_2, \alpha_1, \alpha_2)$, we reconstructed a model spectrum equivalent to the buoy spectrum by applying the exact NOAA reconstruction equation (see https://www.ndbc.noaa.gov/faq/measdes.shtml). In this way, both datasets (buoy and model) share the same directional-spreading limitations, ensuring a more consistent and equitable comparison.

As a result, the validation exhibits a marked improvement and a much more coherent agreement between the Southern Ocean energy identified in both sources. Figure SM3 has been updated accordingly.

In "Trend calculation": not sure that …" consistent with conventions adopted in IPCC" reference is appropriate. To my knowledge IPCC conventions are not that straightforward and are in any case, not that clearly stated in the AR6/Working Group 1 report. But this is a very minor comment and addressing it (or not) should not be any barrier to publication.

Thank you for the comment. We agree that the wording referring explicitly to IPCC conventions was unnecessarily strong. We have revised the text to remove this reference, while keeping the methodology unchanged.

L670-672: *"A trend was considered statistically robust when MK confirmed a significance level below 0.1, a threshold commonly used in climate studies[99,101,102]."*

Finally, I'm slightly uncomfortable with the assertion in the discussion and conclusion that "This approach has enabled a detailed and individualized study of large SO swells for the first time, which would have been difficult to achieve using more traditional approaches on the basis of total integrated parameters." (I have added the italics).

Algorithms for spectral partition analysis and tracking of swells (not just the space-time methods outlined by Delpey et al, 2010), including those with generation zones in the SO, can be widely found in the literature. For example, a quick search turns up:

Zheng, Zhenjun, et al. "Investigation of multimodal wave climate using spectral partitioning and wave system tracking algorithms." Ocean Modelling 188 (2024): 102327.

Algorithms presented in this manucript's study may differ/improve upon in details of previous studies' algorithms, and it is likely the first time swell partition and tracking has been used to identify extremes over such a long coastline at climate timescales (for which I applaud the authors), but (if I am to be pedantic) it seems a very large stretch to say the authors' approach has enabled a detailed and individualized study of large SO swells for the first time.

Thank you for this detailed comment. We agree that the original wording in the Discussion and Conclusions could be interpreted as implying a claim of absolute methodological novelty, which was not our intention. Our goal was not to suggest that spectral partitioning or swell tracking algorithms, including those applied to Southern Ocean swells, have not been previously developed or used, as we fully acknowledge the existence of such approaches in the literature.

Instead, the intended emphasis was on the added value of using spectral wave information, rather than total integrated parameters, to isolate specific wave systems and avoid the masking of secondary swell components, particularly in regions where Southern Ocean swells are not the dominant wave system. This capability enables a more detailed and individualized analysis of large Southern Ocean swell events along the entire Pacific coast of the Americas and at climate timescales, which would be difficult to achieve using traditional integrated-wave approaches alone.

To avoid any potential misunderstanding, we have revised the text accordingly by removing the "for the first time" wording and clarifying that the contribution of this study lies in the scope and scale of the application and in the use of spectral data as input, rather than in the development of new algorithms.

L432-440: *"This study demonstrates that the use of spectral data and the isolation of specific wave systems represent crucial next steps in future impact assessments. Spectral wave information, which is becoming increasingly available, allows for a more precise description of the waves impacting particular coastal areas. The use of spectral data helps prevent unnecessary masking of secondary system behaviors, often overlooked in integrated assessments, such as long-period wave energy, which contributes to significant infragravity wave heights, and long-term trends in key wave properties. These data have enabled a detailed and individualized study of large SO swells along the entire coast of the Americas, which would have been challenging to achieve using more traditional approaches on the basis of total integrated parameters."*
